# FBXO9 Mediates the Cancer-Promoting Effects of ZNF143 by Degrading FBXW7 and Facilitates Drug Resistance in Hepatocellular Carcinoma

**DOI:** 10.3389/fonc.2022.930220

**Published:** 2022-06-30

**Authors:** Zhenyu Wang, Xiaoxia Chen, Lianer Zhou, Xinge Zhao, Chao Ge, Fangyu Zhao, Haiyang Xie, Taoyang Chen, Hua Tian, Hong Li, Jinjun Li

**Affiliations:** ^1^ State Key Laboratory of Oncogenes and Related Genes, Shanghai Cancer Institute, Renji Hospital, Shanghai Jiao Tong University School of Medicine, Shanghai, China; ^2^ State Key Laboratory of Oncogenes and Related Genes, Shanghai Cancer Institute, Renji Hospital, School of Biomedical Engineering, Shanghai Jiao Tong University, Shanghai, China; ^3^ Department of General Surgery, The First Affiliated Hospital, School of Medicine, Zhejiang University, Hangzhou, China; ^4^ Department of Pathology, Qi Dong Liver Cancer Institute, Qidong, China

**Keywords:** hepatocellular carcinoma, FBXO9, ZNF143, FBXW7, ubiquitin-proteasome mediated degradation, Lenvatinib, drug resistance

## Abstract

F-box proteins are critical for malignancy because they control the turnover of key proteins that govern multiple cellular processes. F-box protein 9 (FBXO9) belongs to the F-box protein family and exhibits oncogenic properties in hematological malignancies. However, the function and molecular mechanism of FBXO9 in hepatocellular carcinoma (HCC) remain unclear. Here, we report that FBXO9 was remarkably overexpressed in HCC. Loss- and gain-of-function experiments showed that FBXO9 facilitates HCC cell proliferation and metastasis both *in vitro* and *in vivo*. Mechanistically, as a direct upstream transcription factor, FBXO9 is regulated by zinc finger protein 143 (ZNF143) and accelerates tumor growth and metastasis by targeting the F-box and WD repeat domain containing 7 (FBXW7) for ubiquitination and degradation. Additionally, we found that with FBXO9 knockdown, HCC cells were more sensitive to treatment with lenvatinib and sorafenib. In summary, our results demonstrate that a ZNF143-FBXO9-FBXW7 signaling regulatory axis may be involved in tumor progression in HCC, and suggest that FBXO9 could be a potential biomarker and therapeutic target for HCC.

## Introduction

Liver cancer is the fourth leading cause of cancer-associated decease worldwide (1, 2). Hepatocellular carcinoma (HCC) accounts for approximately four-fifths of primary liver malignancies, imposing a heavy disease burden worldwide (3, 4). Despite considerable progress in treatments for HCC, the limited effect of diagnosis and therapy remains to lead to an inferior prognosis in HCC patients (2, 5). Thus, it is imperative to find more effective diagnostic and treatment strategies through a deeper understanding of the molecular mechanisms underlying HCC progression.

Ubiquitin-mediated proteasomal degradation of the target protein is a critical step in various cellular processes that feature selective protein turnover (6). Dysfunction of the ubiquitin-proteasome system (UPS) usually leads to abnormal levels of oncoproteins or tumor suppressor proteins, in turn leading to carcinogenesis (6, 7). The UPS has three main enzymatic reactions to exert its biological functions, in which E3 ubiquitin ligases are crucial for the specific identification of substrates for ubiquitylation. Among the over 600 supposed E3 ubiquitin ligases in humans, the SKP1–cullin 1–F-box protein (SCF) complex is the most well-characterized (8, 9).

As a target-recognition subunit of SCF complexes, an F-box protein includes at least two primary domains: the F-box domain and the variable domain that bind to specific substrates (10). According to the variable domain, F-box proteins can be divided into three families: FBXO, FBXL, and FBXW (11). In recent years, a series of studies have demonstrated that the expression of F-box proteins can be closely correlated with the occurrence, development, and drug resistance of tumors (12, 13). For example, FBXL1 promotes tumorigenesis by facilitating the degradation of a series of tumor-suppressor proteins, such as such as p21 and p27 (12); FBXO22 degrades nuclear PTEN to promote tumorigenesis (10); and FBXW7 plays a tumor-suppressive role by degrading various cancer-promoting proteins such as mTOR, cyclin E, MCL-1, N-Myc, and SOX9 (12, 14, 15).

However, less attention has been paid to FBXO9 (16). To date, several substrates of FBXO9 have been identified, including Neurog2 (17), PRMT4 (18), p53 (19), PPARγ, BK channel β1 subunit, TEL2, and TTI1 (17, 20). FBXO9 participates in several cellular processes by degrading these substrates. However, the functions and underlying mechanisms of action of FBXO9 in HCC remain unclear. Here, we report that FBXO9, under the control of ZNF143, ubiquitylates and degrades FBXW7 and facilitates HCC proliferation, metastasis, and drug resistance. Our results uncover the pro-oncogenic effect of FBXO9 in HCC and demonstrate that the ZNF143–FBXO9–FBXW7 regulatory pathway is a prospective target for anti-HCC treatment.

## Materials and Methods

### Database Analysis

The data from The Cancer Genome Atlas (TCGA) (https://tcga-data.nci.nih.gov/tcga/), Gene Expression Omnibus (GEO) (https://www.ncbi.nlm.nih.gov/geo/, accession codes GSE14520 and GSE76427), and Gene Expression Profiling Interactive Analysis (GEPIA) (http://gepia.cancer-pku.cn) databases were downloaded from their website. The transcription factor-binding sites of FBXO9 were predicted using the UCSC Genome Browser (http://genome.ucsc.edu/index.html) and the JASPAR database (http://jaspar.genereg.net/). Protein interactions were predicted using STRING database (version 11.0b, https://version-11-0b.string-db.org/).

### Human Specimens

Human HCC specimens and paired adjacent nontumorous liver tissues were obtained from Zhejiang University (Hangzhou, China), Guangxi Cancer Institute (Guangxi, China), and Qidong Liver Cancer Institute (Qidong, China) with informed consent. None of the patients had received chemotherapy or radiation therapy. Clinical characteristics of tissue donors are shown in **Supplementary Table S1**. The Research Ethics Committee of Renji Hospital reviewed and approved all the protocols involving human tissues used in this study.

### Cells and Cell Culture

The HCC-LY10 and HCC-LY5 cell lines were established in our laboratory. THLE-3, MIHA, and L02 cells were provided by the Cell Bank of the Institute of Biochemistry and Cell Biology (Shanghai, China). Li7 was purchased from BioLeaf (Beijing, China). HEK-293T, PLC/PRF/5, and Hep3B cells were purchased from the American Type Culture Collection. Huh7 cells were purchased from the Riken Cell Bank (Tsukuba, Japan). MHCC-LM3, MHCC-97H, and MHCC-97L cells were kindly provided by Zhongshan Hospital (Shanghai, China). Cells were cultured at 37°C in a humidified atmosphere containing 5% CO_2_ using complete Dulbecco’s modified Eagle medium (DMEM, #11965092; Gibco, Grand Island, NY, USA) containing 10% fetal bovine serum (FBS, #10100147C; Gibco) and 1% penicillin/streptomycin (#15070063, Gibco). The supplier authenticated and characterized the cells within the previous six months. All cells were free of mycoplasma contamination and tested using PCR.

### Plasmid Constructs, Lentivirus Production, and Cell Transduction

Short hairpin RNA (shRNA) targeting FBXO9 (shFBXO9#1/shFBXO9#2), FBXW7 (shFBXW7#1/shFBXW7#2), and their negative control (shNC) were purchased from GeneCopoeia (Rockville, USA). Human FBXO9 cDNA (NM_033480.2) and FBXW7 cDNA (NM_033632.3) were subcloned into Lv201 or Lv105 vector (GeneCopoeia). The ZNF143 related plasmids (ZNF143/shZNF143#1/shZNF143#2) are as described in our previous study (21). All the shRNA target sequences are listed in **Supplementary Table S2**. Lentivirus production and cell transfection were performed as previously described (21, 22).

### Quantitative Real-Time PCR

TRIzol reagent (15596026, Invitrogen, Carlsbad, CA, USA) and the PrimeScript RT Reagent Kit (RR037A, TaKaRa Bio, Japan) were used to obtain cDNA. TB Green Premix Ex Taq II (RR820A, TaKaRa Bio, Japan) and an Applied Biosystems 7500 real-time PCR platform (Thermo Fisher Scientific, USA) were used for qPCR assays, following the manufacturer’s instructions. Relative gene expression was quantified using the ΔΔCt method and was normalized to that of GAPDH. The primer sequences are listed in **Supplementary Table S3**.

### Western Blotting

Cells or tissues were lysed using radio-immunoprecipitation assay (RIPA) buffer (#89901, Thermo Scientific, Waltham, MA, USA) containing a phosphatase inhibitor (#04906837001; Roche, Basel, Switzerland) and protease inhibitor cocktail (#04693132001; Roche). After denaturing and SDS-PAGE, the proteins were transferred to polyvinylidene difluoride membranes (#IPVH00010; Millipore, Bedford, MA, USA). The membranes were blocked and incubated with primary antibodies at 4°C overnight. HRP-conjugated secondary antibodies were used to probe the proteins, and the results were visualized with a chemiluminescent substrate (#34580; Thermo Scientific) using a chemiluminescence analyzer (Bio-Rad, Hercules, CA, USA). Antibody information is listed in **Supplementary Table S4**.

### Cell Counting Kit-8 Assay

Cells were seeded in 96-well plates, and cell viability was measured using the CCK-8 assay (#B34304; Bimake, Houston, TX, USA) at the designated times.

### Colony Formation Assay

For the colony formation assay, cells were seeded in 6-well plates and incubated for approximately 2 weeks. Colonies were fixed and stained using Giemsa stain (#G5637-5G; Sigma-Aldrich, St. Louis, MO, USA).

### Drug Sensitivity Assay

For short-term viability assays, serially increasing dilution concentrations of lenvatinib (#S1164; Selleck, Houston, USA) and sorafenib (#S7397; Selleck) were prepared. The cells were seeded in 96-well plates and exposed to these drugs for 72 h. Cell viability was quantified using a CCK-8 assay, and the relative cell viability and IC_50_ values were calculated. For long-term clonogenic assays, cells were seeded in 6-well plates and exposed to increasing concentrations of lenvatinib and sorafenib for 14 days. The colonies were then fixed and stained with Giemsa.

### Flow Cytometry Analysis

Cells were seeded in 6-well plates. After adherence, the cells were treated with 10 μM lenvatinib for 24 h. The cells were digested with EDTA-free trypsin and stained using an eBioscienceTM Annexin V Apoptosis Detection Kit (#88-8007, Thermo Scientific) according to the manufacturer’s instructions. Then, Flow cytometry analysis was performed within 4 h.

### EdU Incorporation Experiment and Annexin V Staining

EdU incorporation experiment and Annexin V staining were performed using the EdU-555 cell proliferation kit (#C0075S; Beyotime, Shanghai, China) and Annexin V-mCherry apoptosis detection kit (#C1069S, Beyotime), respectively, by following the manufacturer’s instructions. The images were captured using an Olympus CKX53 microscope.

### *In Vitro* Transwell Assays

Transwell assays were performed to examine *in vitro* invasion and migration. Cells were seeded in the upper chamber of the transwell apparatus (8 μm, MCEP24H48, Sigma-Aldrich) with or without Matrigel coating (#354230; BD Biosciences, San Jose, CA, USA), and cultured in serum-free DMEM. Complete DMEM was added to the lower side of the chamber as a chemoattractant. The non-invaded or non-migrated cells were removed from the upper chamber after 24–48 hours of incubation. After fixing, crystal violet (#E607309-0100; Sangon Biotech, Shanghai, China) was used to stain the remaining cells, which were then counted in three randomly selected visual fields.

### Mice

Five- to seven-week-old male BALB/c nude mice were purchased and housed in the SPF animal facility at our institute. For the mouse liver orthotopic transplantation assay, 2 × 10^6^ Li7 cells infected with FBXO9/vector, and 2 × 10^6^ MHCC-LM3 cells infected with shFBXO9#1/shFBXO9#2/shNC in 20 μl serum-free DMEM were mixed with 20 μl Matrigel in an ice bath. Under anesthesia, the cells were injected into the murine liver as quickly as possible. Mice were sacrificed after 6–8 weeks of housing and liver weights (including tumor weights) and tumor sizes were measured. Lung and liver tissues were collected from the mice and fixed for histological examination. For mouse subcutaneous tumor xenograft assays, 3 × 10^6^ MHCC-LM3 cells infected with shFBXO9#1/shNC were resuspended in 200 μl serum-free DMEM and injected into the left axillary fossa. Following tumor formation, the mice were randomly allocated and treated with 10 mg/kg lenvatinib orally every alternate day. The tumor size was measured during lenvatinib treatment. After three weeks of lenvatinib treatment, the mice were sacrificed, and tumors were removed and weighed. All experimental animal protocols were approved by the Animal Care and Use Committee of Renji Hospital and performed according to their guidelines.

### Chromatin Immunoprecipitation Assay

The EZ-Magna ChIP assay kit (MAGNA0017, Millipore) was used for the ChIP assay. Briefly, PLC/PRF/5 and HCC-LY10 cells were cross-linked and lysed. The cell lysates were sonicated to shear the DNA to fragments of 500–1,000 bp. The DNA-containing complexes were immunoprecipitated using an anti-ZNF143 (#16618-1-AP; Proteintech, Rosemont, IL, USA) antibody or IgG (Millipore). After reverse crosslinking, qPCR was performed to probe the ZNF143-binding site in the FBXO9 promoter region. The primer sequences are listed in **Supplementary Table S3** and the antibody information is listed in **Supplementary Table S5**.

### Dual-Luciferase Assay

The FBXO9 promoter (bp -1150/+200 relative to the ATG start codon) and two 5’-truncated sequences of the FBXO9 promoter were cloned into the dual-luciferase reporter gene vector pEZX-FR01 (GeneCopoeia). Lipofectamine 2000 (11668030, Invitrogen) was used to transfect the corresponding dual-luciferase reporter along with ZNF143 or the vector plasmid into Li7 and HCC-LY10 cells. Two days later, luciferase activity of the cells was detected using a dual-luciferase reporter gene assay system (E1980, Promega, Madison, WI, USA).

### Coimmunoprecipitation (Co-IP) Assay

Cells were harvested using RIPA lysis buffer, and cell lysates were immunoprecipitated with protein A/G agarose beads (#IP05-1.5ML; Sigma-Aldrich) and antibodies overnight with rotation at 4°C. The beads were then washed, and the immunoprecipitates were subjected to western blotting. Details of the antibodies used are listed in **Supplementary Table S5**.

### Statistical Analysis

GraphPad Prism 7 software and ImageJ software were used for statistical analyses. Statistical comparisons of the data were performed using a two-tailed Student’s *t*-test for two-group comparisons or one-way ANOVA for multiple comparisons. Kaplan–Meier method was used to generate survival curves, and the log-rank test was used for comparison. The correlation between the two molecules was analyzed using Pearson correlation. All data are shown as mean ± SD. Statistical significance was set at P < 0.05.

## Results

### FBXO9 Is Upregulated in HCC

**Figure 1:**
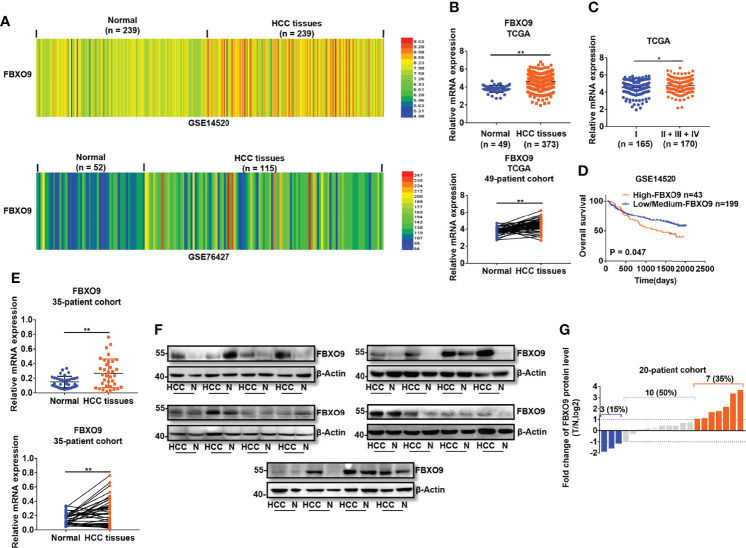
FBXO9 is upregulated in HCC. **(A, B)** The expression of FBXO9 in HCC tissues and noncancerous liver tissues in GSE14520, GSE76427, and TCGA database. **(C)** Expression of FBXO9 in HCC tissues of early pathological stage (Stage I) and advanced pathological stage (Stage II-IV) in TCGA database. **(D)** Kaplan-Meier analysis of overall survival in patients with low/medium expression or high expression of FBXO9 in GSE14520 dataset. **(E)** qPCR analysis (above, two-sided unpaired *t*-test; below, two-sided paired *t*-test) of FBXO9 transcription in 35 paired HCC tissues and paired nontumorous tissues in our lab. **(F)** Western blot of FBXO9 expression in 20 paired HCC tissues (HCC) and normal (N) samples in our lab. **(G)** Waterfall plot showing the protein level of FBXO9 in HCC tissues compared with adjacent noncancerous tissues from 20 patients, as determined by western blotting. Red histogram, FBXO9 overexpressed more than two times in HCC tissues compared with normal tissues; blue histogram, FBXO9 decreased more than two times; grey histograms, FBXO9 overexpressed or decreased by no more than twice. The data are presented as mean ± SD, *, P < 0.05, **, P < 0.01 by two-tailed Student's *t*-test.

To study the functions of FBXO9 in HCC, we first investigated the mRNA transcription of FBXO9 in human primary HCC using the GEO and TCGA databases. The transcription of FBXO9 was remarkably increased in HCC tissues compared to that in nontumorous liver tissues (**Figures 1A, B**). Moreover, we found higher expression of FBXO9 in the cancer tissues of patients in the advanced pathological stages than in those in early pathological stages (**Figure 1C**). Next, we analyzed the relationship between FBXO9 expression and overall survival in HCC patients using GSE14520 dataset. The data showed that patients with FBXO9 overexpression exhibited worse overall survival (**Figure 1D**). We then evaluated the expression of FBXO9 in human HCC tissues and paired liver tissues from our laboratory. Aligned with the data from the databases, we found that human primary HCC tissues upregulated FBXO9 expression compared with noncancerous liver tissues (**Figures 1E–G**). Thus, these findings reveal that FBXO9 is overexpressed in HCC.

### FBXO9 Accelerates HCC Cell Growth

**Figure 2:**
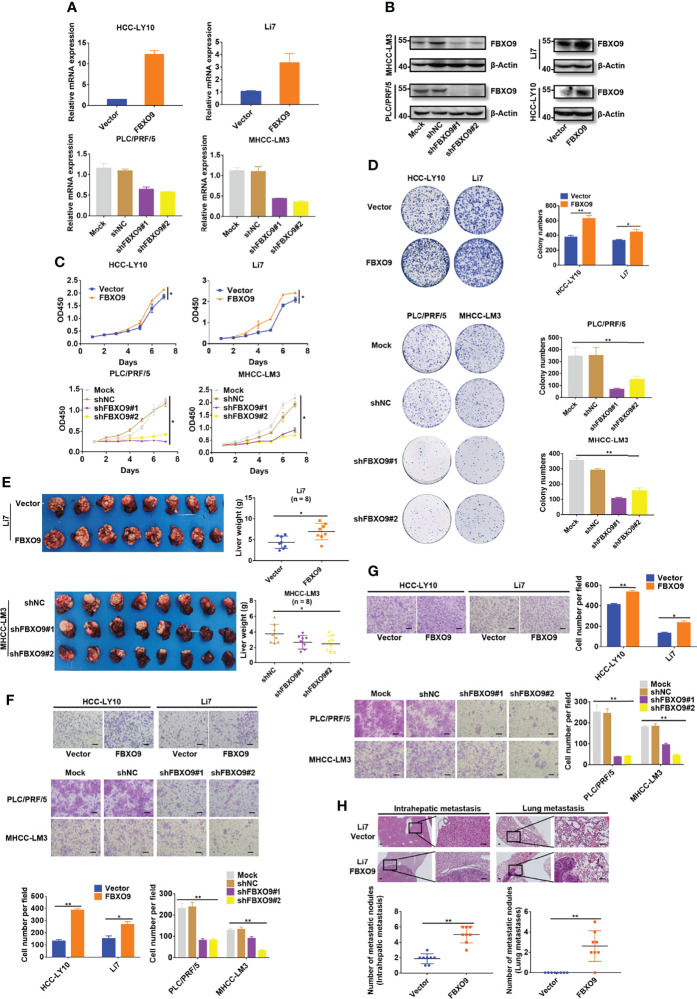
FBXO9 increases HCC cell growth and mobility. **(A, B)** qPCR and western blotting of FBXO9 expression in FBXO9-overexpressing and FBXO9-knockdown HCC cells. **(C)** The growth curve of FBXO9 overexpressed or knocked down HCC cells was generated by CCK-8 assays. **(D)** Representative images and quantitative data of the colony formation of FBXO9 overexpressed or knock down HCC cells. **(E)** Liver tissues from xenograft-bearing animals. Quantitative analysis of liver weight was shown in the scatter plot graphs (n = 8). **(F, G)** Representative images of the transwell assays of FBXO9 function in HCC cell migration and invasion. Scatter plot graphs show the quantitative data. **(H)** H&E staining images of intrahepatic and lung metastatic nodules of the vector or FBXO9 overexpression groups. The numbers of metastatic nodules per mouse are presented in the scatter plot graphs (n = 8). Data are shown as the mean ± S.D, n = 3. Scale bars: 100 μm. *, P < 0.05, **, P < 0.01 by two-tailed Student’s *t*-test or one-way ANOVA.

To further reveal how FBXO9 affects HCC cell growth, we examined FBXO9 expression in a series of HCC cell lines and immortalized hepatocyte cell lines using qPCR and western blotting. HCC cells were selected for gain-of-function or loss-of-function experiments because of their high or low FBXO9 levels (**Supplementary Figures S1A, B**). Based on this, we established stable FBXO9-overexpressing (FBXO9/Vector) cell lines with HCC-LY10 and Li7 cells and FBXO9 knockdown (Mock/shNC/shFBXO9#1/shFBXO9#2) cell lines with PLC/PRF/5 and MHCC-LM3 cells *via* lentiviral transfection. The expression of FBXO9 in FBXO9-knockdown or overexpressing HCC cells was verified using qPCR and western blotting (**Figures 2A, B**). As shown in CCK-8 and colony formation assays, enhanced growth of HCC cells with FBXO9 overexpression and reduced growth of HCC cells with FBXO9 knockdown were observed (**Figures 2C, D**). Next, we performed EdU incorporation and annexin V staining (**Supplementary Figure S2**) to investigate whether FBXO9 accelerated HCC cell growth by promoting cell proliferation or inhibiting cell death. The results showed that FBXO9 markedly inhibited cell death and promoted cell proliferation. Next, we explored the effects of FBXO9 on HCC *in vivo*, using an orthotopic mouse xenograft model. In line with the *in vitro* results, the size and weight (liver with xenograft) of tumors derived from FBXO9-overexpressing Li7 cells were significantly increased compared with those of tumors derived from negative control cells. In contrast, tumors transfected with shFBXO9#1 or shFBXO9#2 showed lower weights and smaller sizes than tumors transfected with shNC in MHCC-LM3 cells (**Figure 2E**, **Supplementary Figure S3**). Taken together, our findings suggest that FBXO9 promotes HCC cell growth both *in vitro* and *in vivo*.

### FBXO9 Promotes HCC Metastasis

Next, we investigated whether FBXO9 affected HCC cell migration and invasion *in vitro*. The results of *in vitro* cell mobility (transwell) experiments (with or without Matrigel) showed that the overexpression of FBXO9 promoted the migration and invasion of HCC cells *in vitro*, whereas its knockdown suppressed HCC cell migration and invasion *in vitro* (**Figures 2F, G**).

To explore the effects of FBXO9 on HCC metastasis *in vivo*, paraffin sections of liver and lung tissues with xenograft and/or metastatic nodules were prepared, and hematoxylin-eosin staining was performed. Mice overexpressing FBXO9 exhibited more metastatic nodules in the liver and lungs than the control mice (**Figure 2H**). Collectively, our findings suggest that FBXO9 markedly facilitates HCC metastasis both *in vitro* and *in vivo*.

### FBXO9 Is a Direct Downstream Target Gene of ZNF143

**Figure 3:**
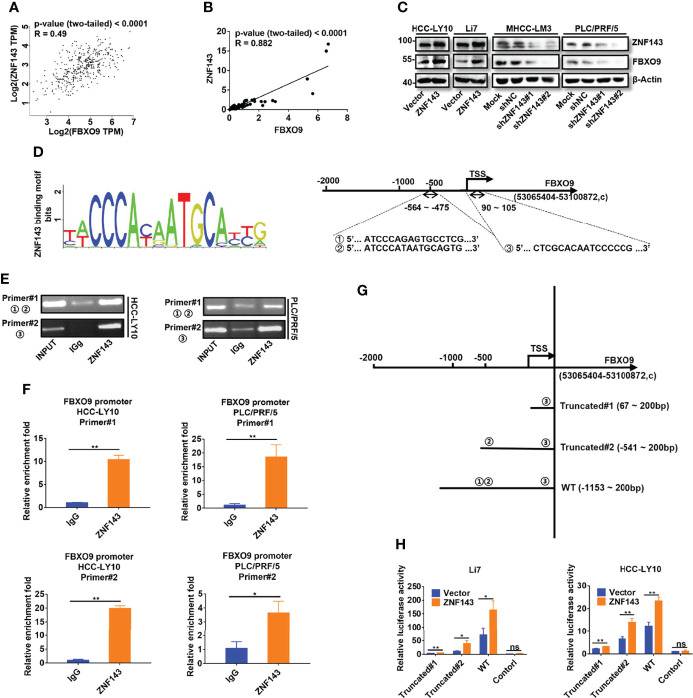
ZNF143 binds to the FBXO9 promoter and promotes FBXO9 expression. **(A)** Verification of the correlation of ZNF143 and FBXO9 using the GEPIA database. **(B)** Scatter plots of the correlation between ZNF143 mRNA expression and FBXO9 mRNA expression in 64 HCC tissues from HCC patients. **(C)** The expression level of ZNF143 and FBXO9 as detected by western blotting in HCC cells after knockdown or overexpression of ZNF143. **(D)** Left, ZNF143 binding motif. Right, JASPAR analysis showed three potential ZNF143-binding sites of the promoter region of FBXO9. **(E)** Agarose gel electrophoresis for CHIP analysis of ZNF143 binding to the FBXO9 promoter in HCC-LY10 and PLC/PRF/5 cells. **(F)** CHIP-qPCR analysis of ZNF143 binding to the FBXO9 promoter in PLC/PRF/5 and HCC-LY10 cells. **(G)** Different luciferase reporter vectors containing truncation mutants of FBXO9 were constructed and transfected into Li7 and HCC-LY10 cells. **(H)** The corresponding luciferase activities were determined using dual-luciferase reporter assays. Error bars represent mean ± S.D, n = 3. R, Pearson correlation coefficient. *, P < 0.05; **, P < 0.01; ns, no significance by two-tailed Student’s *t*-test or one-way ANOVA.

To further understand the role of FBXO9 in HCC, we attempted to identify the potential transcription factors of FBXO9 using the UCSC Genome Browser and JASPAR databases. As shown in **Supplementary Figure S4**, 19 common transcription factors were predicted by two databases, and only six transcription factors (SP1, CREB1, ZNF143, NRF1, RFX1, and YY1), which have a Pearson correlation coefficient (Pearson-CC) greater than 0.45 with FBXO9 in TCGA. Among these transcription factors, ZNF143 attracted our attention. The abnormal expression of ZNF143 is associated with tumor progression, as reported in our previous study (21) and other studies (23, 24). However, the relationship between ZNF143 and SCF complex has not been fully elucidated. Thus, we first investigated ZNF143 and FBXO9 expressions in HCC and matched non-tumorous liver tissues using the GEPIA database and our own samples. The transcription level of FBXO9 was positively correlated with ZNF143 expression (**Figures 3A, B**). We also found that the mRNA transcription of FBXO9 and ZNF143 was positively correlated in a series of HCC and immortalized liver cell lines (**Supplementary Figures S1A**, **S5**). We then established cell lines with stable ZNF143 overexpression (ZNF143/Vector) using Li7 and HCC-LY10 cells and stable cell lines with ZNF143 knockdown (Mock/shNC/shZNF143#1/shZNF143#2) in PLC/PRF/5 and MHCC-LM3 cells. Western blotting demonstrated that the overexpression of ZNF143 promoted the expression of FBXO9, whereas knockdown of ZNF143 inhibited the expression of FBXO9 (**Figure 3C**). Next, we analyzed the possible ZNF143 binding sites in the FBXO9 promoter using the JASPAR database. The data showed three putative binding sites (represented by ①, ②, and ③, respectively) located at -564 to -549 bp (①), -490 to -475bp (②), and +90 to +105 bp (③) relative to the TSS (**Figure 3D**). A ChIP assay demonstrated that ZNF143 could directly bind to the promoter of FBXO9 in HCC cells (**Figures 3E, F**). Furthermore, we constructed three different dual-luciferase reporter plasmids using a 1350 bp FBXO9 promoter region (−1150 to +200 bp relative to the TSS) or two truncated clones of the region (truncated#1:67 to 200 bp relative to the TSS; truncated#2: -541 to 200 bp relative to the TSS; **Figure 3G**). Luciferase assays revealed that compared with the control vector, all the constructs exhibited higher promoter activity in Li7 and HCC-LY10 cells and could be induced by ZNF143 overexpression, mainly in the region of -564 to -549 bp (**Figure 3H**). Taken together, our results indicate that ZNF143 is a transcription factor for FBXO9.

### FBXO9 Mediates the Cancer-Promoting Effect of ZNF143 in HCC

**Figure 4:**
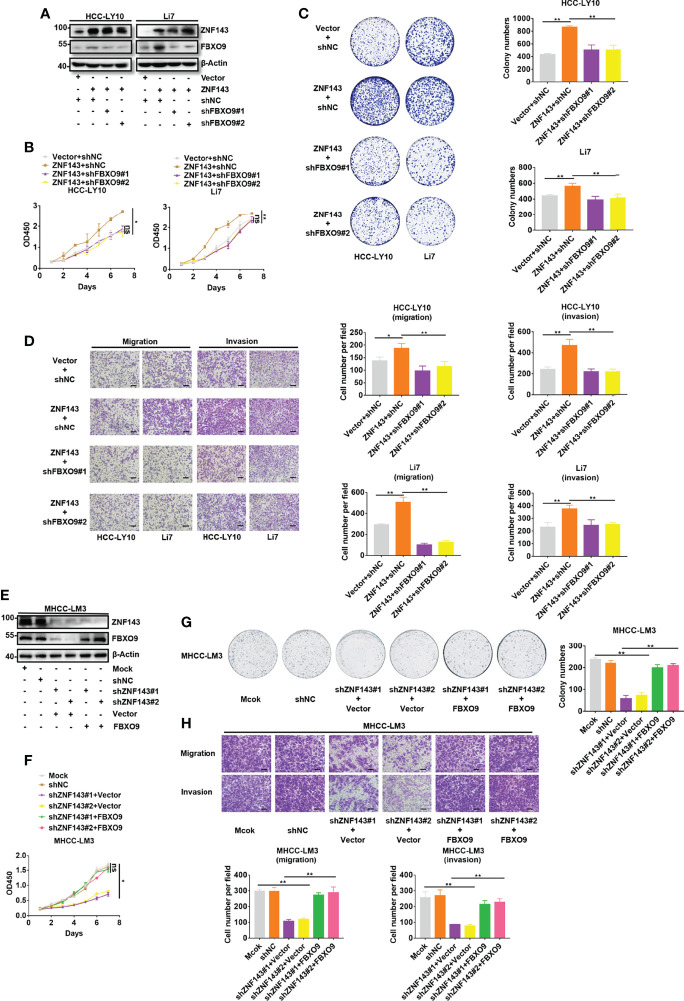
ZNF143 promotes HCC cell growth and mobility by enhancing FBXO9 expression. **(A)** ZNF143-overexpressing HCC cells were transfected with shNC or shFBXO9, and the knockdown efficiency of FBXO9 was tested by western blotting. **(B, C)** The effects of FBXO9 silencing on ZNF143-induced cell growth in Li7 and HCC-LY10 cells were measured by CCK-8 and *in vitro* colony formation assays. **(D)** Transwell assays of cell migration and invasion in ZNF143-overexpressing HCC cells treated with shFBXO9. **(E)** FBXO9 plasmid was transfected into ZNF143 knockdown HCC cells, and the protein expression levels of FBXO9 and ZNF143 were detected using western blotting. **(F, G)** Effects of FBXO9 overexpression on the proliferation of ZNF143 knockdown HCC cells were measured by CCK-8 and colony formation assays. **(H)** Transwell assays of cell migration and invasion in ZNF143 knockdown HCC cells treated with FBXO9 plasmid. Data are shown as mean ± S.D, n = 3. Scale bars: 100 μm. *, P < 0.05; **, P < 0.01; ns, no significance, by two-tailed Student’s *t*-test or one-way ANOVA.

To explore whether the upregulation of FBXO9 expression is a key part of the cancer-promoting effect of ZNF143 in HCC, plasmids with shFBXO9 were transfected into ZNF143-overexpressing cells to silence FBXO9 expression. Western blotting showed that FBXO9 knockdown effectively reversed FBXO9 upregulation induced by the overexpression of ZNF143 (**Figure 4A**). The results of the CCK-8 cell proliferation assay and cell colony formation assay indicated that FBXO9 knockdown significantly reduced HCC cell proliferation induced by ZNF143 overexpression (**Figures 4B, C**). Furthermore, *in vitro* Transwell assays demonstrated that FBXO9 knockdown markedly inhibited the invasion and migration capacities of ZNF143-overexpressing HCC cells (**Figure 4D**). In addition, overexpression of FBXO9 rescued the proliferation and metastatic capacity of the ZNF143-knockdown HCC cells (**Figures 4E–H**). Thus, our results confirm that FBXO9 mediates the cancer-promoting effect of ZNF143 in HCC.

### FBXO9 Promotes HCC Cell Growth and Metastasis by Targeting FBXW7 for Ubiquitination and Degradation

**Figure 5:**
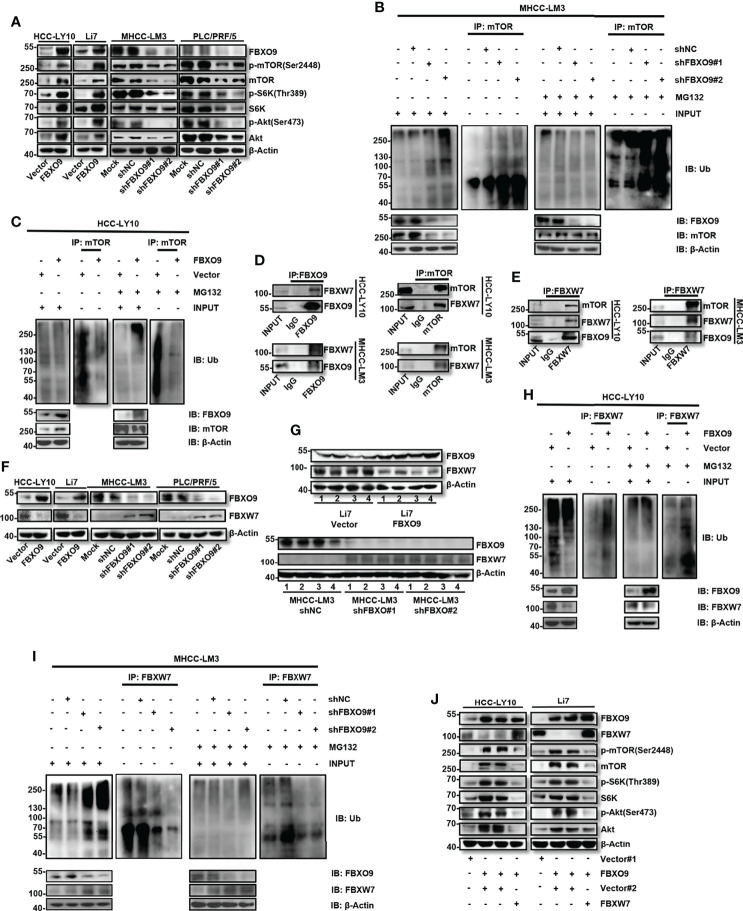
FBXO9 exerts its cancer-promoting effect by interacting with FBXW7 and promoting ubiquitination and degradation of FBXW7 in HCC. **(A)** Western blotting of the levels of p-mTOR, p-S6K, p-Akt, total mTOR, S6K, Akt, and FBXO9 in HCC cells with FBXO9 overexpression or knockdown. **(B, C)** HCC cell with FBXO9-overexpressing or FBXO9-knockdown were treated with 10 μM MG132 or DMSO for 6 h, lysates were immunoprecipitated with an anti-mTOR antibody under native conditions. The resultant immunocomplexes and lysates (Input) were immunoblotted with antibodies against indicated antibodies. **(D, E)** Endogenous FBXO9, mTOR, or FBXW7 were immunoprecipitated with antibodies as indicated. The proteins in immunocomplexes were respectively detected using anti-FBXO9, anti-FBXO9, or anti-FBXW7 antibody by western blotting. **(F, G)** Western blotting of the protein levels of FBXO9 and FBXW7 in FBXO9-overexpressing or knock down HCC cells or xenograft tumors. **(H, I)** HCC cell with FBXO9-overexpressing or FBXO9-knockdown were treated with 10 μM MG132 or DMSO for 6 h, lysates were immunoprecipitated with an anti-FBXW7 antibody under native conditions. The resultant immunocomplexes and lysates (Input) were immunoblotted with antibodies against indicated antibodies. **(J)** Western blotting showed the protein levels of FBXO9, FBXW7, p-mTOR, p-S6K, p-Akt, total mTOR, S6K, and Akt in FBXO9-overexpressing HCC cells after FBXW7-overexpression.

mTOR is a well-known oncogene that is activated in various tumors (25–27). A recent study showed that SCFFbxo9 ubiquitin ligase could regulate mTOR signaling to direct the cellular response to growth factor withdrawal and switch to a state of nutritional saving and maintenance of survival (20). Therefore, we assessed the protein levels of mTOR in FBXO9-knockdown or -overexpressing HCC cells. Our results showed that the overexpression of FBXO9 increased the expression of mTOR and activated the mTOR pathway; conversely, knockdown of FBXO9 decreased mTOR expression and inhibited the mTOR pathway (**Figure 5A**). Next, because FBXO9 is a subunit of the SCF complex, we determined whether the interaction between FBXO9 and mTOR could alter the ubiquitination level of mTOR. Our results showed that ubiquitination of mTOR in HCC cells was inhibited by FBXO9 overexpression, whereas facilitated by FBXO9 knockdown (**Figures 5B, C**). These results indicate that there may be other molecules that can ubiquitinate mTOR were ubiquitinated by FBXO9. To further study the mechanism underlying the function of FBXO9 in HCC, top 10 possible functional partners of FBXO9 were predicted using the STRING database (version 11.0b, **Supplementary Figures S6A, B**). Among these proteins, FBXW7 attracted the most attention (**Supplementary Figure S6C**). It is well known that FBXW7 serves as a tumor suppressor that promotes ubiquitination of a wide range of oncoproteins for proteasomal degradation, including mTOR (14, 15, 28–30). In line with this, our results also showed that FBXW7 overexpression significantly inhibited the proliferation and metastasis of HCC cells *in vitro* (**Supplementary Figure S7**).

To explore the interaction between FBXO9, FBXW7, and mTOR, a Co-IP assay of endogenous proteins in HCC cells was performed. FBXW7 co-precipitated with antibodies targeting FBXO9 or mTOR (**Figure 5D**). FBXO9 and mTOR also co-precipitated with anti-FBXW7 antibodies (**Figure 5E**). We then investigated the protein expression levels of FBXW7 in FBXO9 overexpressing and knock down HCC cells. Western blotting revealed that FBXO9 overexpression decreased the protein level of FBXW7, and FBXO9 knockdown increased the expression of FBXW7, both *in vitro* (**Figure 5F**) and *in vivo* (**Figure 5G**). In addition, we found that the protein expression levels of FBXO9 and FBXW7 were opposite in most HCC cell lines (**Supplementary Figure S8**). Furthermore, we investigated whether FBXO9 regulated the protein level of FBXW7 by inducing FBXW7 ubiquitination and degradation. The ubiquitination of FBXW7 in HCC cells was facilitated by FBXO9 overexpression and inhibited by FBXO9 knockdown (**Figures 5H, I**). Moreover, our results demonstrated that FBXW7 overexpression effectively reversed mTOR upregulation and activation induced by FBXO9 overexpression (**Figure 5J**). These results indicate that FBXO9 can directly interact with FBXW7, leading to the ubiquitination and subsequent degradation of FBXW7.

To further investigate the effects of FBXW7 on FBXO9-promoted HCC cell proliferation and metastasis, we overexpressed FBXW7 under FBXO9 overexpression in HCC cells. We found that FBXW7 overexpression rescued FBXO9 overexpression-induced cell proliferation, migration, and invasion translocation in HCC cells (**Supplementary Figures S9A–C**). Taken together, these results indicated that FBXO9 promotes HCC cell growth and metastasis by targeting FBXW7 for ubiquitination and subsequent degradation.

### Knockdown of FBXO9 Reduces Drug Resistance of HCC Cells

**Figure 6:**
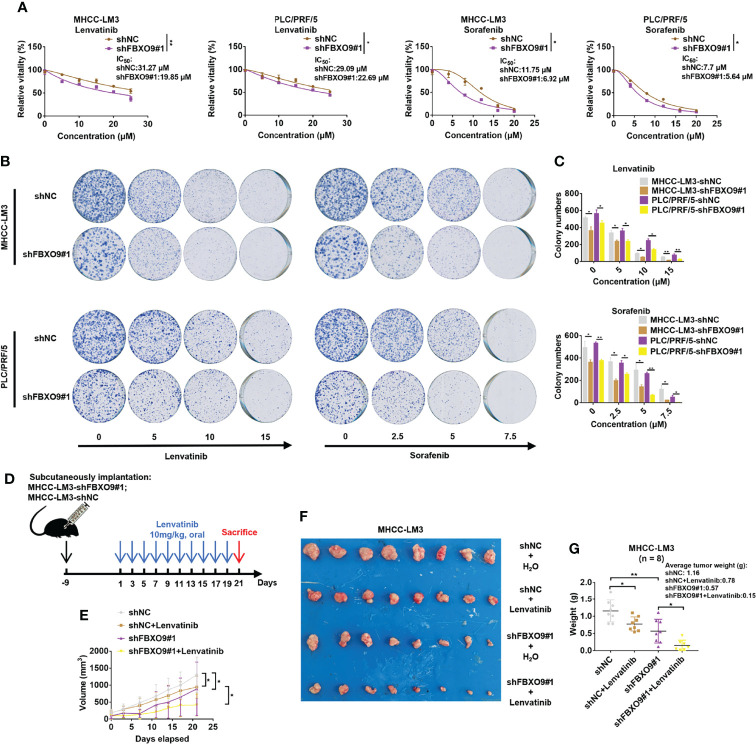
Knockdown of FBXO9 increases chemosensitivity of HCC cells. **(A)** FBXO9 knockdown HCC cells were treated with Lenvatinib (0–25 µM) or sorafenib (0–20 µM) for 72 h and cell viabilities were assayed. **(B)** Effects of lenvatinib and sorafenib on MHCC-LM3 cells with FBXO9 knockdown were analyzed by using long-term colony-formation assays. **(C)** Quantitative analysis of colony-formation assays. **(D, E)** MHCC-LM3-shFBXO9#1/MHC-LM3-shNC cells were subcutaneously injected into nude mice. After 10 days of tumor formation, the mice were treated with 10 mg/kg lenvatinib orally every other day, and the tumor growth curves of mice were analyzed. **(F, G)** Both lenvatinib treatment and FBXO9 knockdown inhibited tumor growth, and FBXO9 knockdown combined with lenvatinib treatment more effectively abolished tumor growth in mice (n = 8). Error bars are shown as the mean ± SD, n = 3. *, P < 0.05; **, P < 0.01 by two-tailed Student *t*-test or one-way ANOVA.

Many studies have shown that F-box proteins play indispensable roles in drug resistance (6, 12). As the target protein of FBXO9, FBXW7 has been reported to increase chemosensitivity of HCC cells (14, 31). Accordingly, we explored the effects of FBXO9 knockdown on the sensitivity of HCC cells to lenvatinib and sorafenib, the two first-line drugs for HCC therapy. HCC cells were exposed to increasing concentrations of lenvatinib or sorafenib, and cell viability was determined after 72 h of exposure. The results were calculated as the dose-dependent inhibition curves and IC_50_ values. As shown in **Figure 6A**, knockdown of FBXO9 reduced the IC_50_ of lenvatinib and sorafenib in HCC cells. Furthermore, flow cytometry analysis indicated that the increase in the percentage of apoptotic cells in FBXO9-knockdown HCC cells after lenvatinib treatment was higher than that in control cells (**Supplementary Figure S10**). Subsequently, we analyzed the effects of FBXO9 knockdown on long-term resistance of HCC cells to lenvatinib and sorafenib using clonogenic assays. HCC cells with FBXO9 knockdown were more vulnerable to the effects of lenvatinib and sorafenib than the corresponding control cells (**Figures 6B, C**).

For further confirmation, MHCC-LM3 cells with a stable knockdown of FBXO9 and their control cells were subcutaneously injected into nude mice. Following tumor formation, mice were randomly allocated and treated with lenvatinib or water (**Figure 6D**). Our results showed that both FBXO9 knockdown and lenvatinib treatment inhibited tumorigenicity, and knockdown of FBXO9 enhanced the tumor growth inhibition of lenvatinib (**Figures 6E–G**). Taken together, these results provide strong evidence that FBXO9 knockdown sensitizes HCC cells to lenvatinib and sorafenib.

### Clinical Correlations Between FBXO9 With ZNF143 and FBXW7

**Figure 7:**
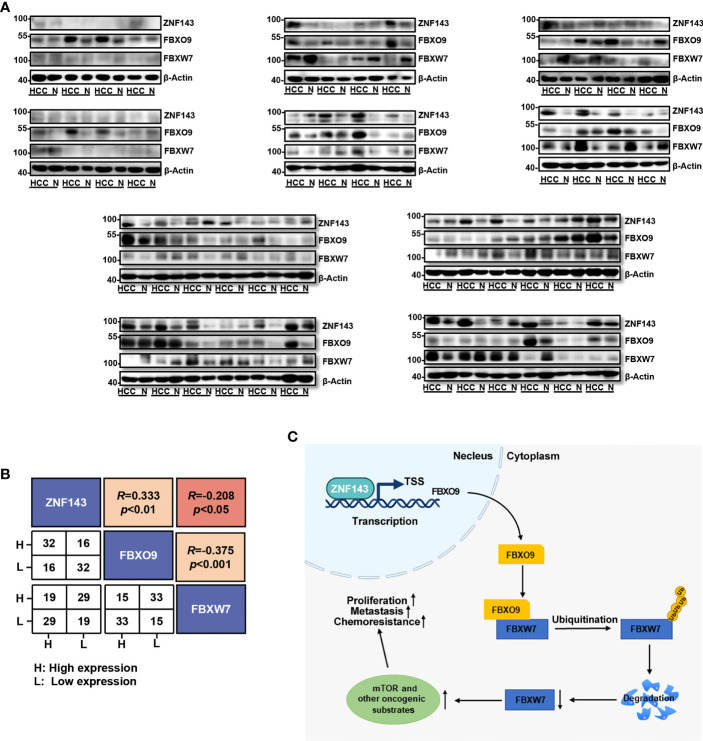
Correlation of ZNF143, FBXO9 and FBXW7 in HCC samples. **(A)** Western blotting analysis of FBXO9, ZNF143, and FBXW7 expression in HCC and paired noncancerous tissues (N). **(B)** Correlation analysis of ZNF143, FBXO9, and FBXW7 expression trends shown in Western blotting analysis of panel **(A)**. **(C)** Pattern diagram of the ZNF143-FBXO9-FBXW7 axis. Under the transcriptional regulation of the ZNF143, FBXO9 directly interacts with FBXW7 and induces ubiquitination and degradation of FBXW7 and thus leads to an increase in oncogenic substrates of FBXW7, which ultimately promotes the progression and drug resistance of HCC. R, Pearson correlation coefficients.

To further understand the critical role of FBXO9, we used western blotting to analyze the protein expression of ZNF143, FBXO9, and FBXW7 in HCC clinical samples and matched non-cancerous liver tissues from 48 patients. Our results revealed that the protein levels of ZNF143 and FBXO9 were higher in most HCC tissues than in their paired noncancerous liver tissues, whereas the expression of FBXW7 was the opposite (**Figure 7A**). Furthermore, the expression trend of FBXO9 positively correlated with ZNF143 expression trend and negatively correlated with FBXW7. Meanwhile, the expression trend of FBXW7 was opposite to that of ZNF143 and FBXO9 (**Figure 7B**).

## Discussion

In recent years, F-box proteins have been identified as essential factors for malignancies *via* target degradation (12, 28). FBXO9 is a mysterious member of the F-box protein family (16), and a few available studies have shown that FBXO9 is involved in the development of the nervous system or adipose tissue (17, 18), in pathological changes in the muscle in diabetes (17) and epithelial damage in respiratory distress syndrome (18), and in the adverse effects of zoledronic acid (19) or as a target of sepsis (18), and participates in disease progression in hematological tumors by regulating the activity of the mTOR pathway (20) and the proteasome (8). However, the biological functions and mechanisms underlying the role of FBXO9 in HCC have not been reported. In this context, we present a novel finding that FBXO9 acts as an oncoprotein in HCC. Here, we investigated the expression of FBXO9 using the databases and human HCC samples from our lab, and found that FBXO9 was upregulated in HCC. Furthermore, our functional research indicated that FBXO9 promotes HCC cell growth and metastasis *in vitro* and *in vivo*.

FBXW7 is a member of the F-box family. It is a well-established tumor inhibitor that targets a series of oncogenic proteins for degradation (14, 15, 32) and plays a powerful tumor suppressor role in a variety of tumors, including HCC (15, 29, 30, 32, 33). Some proteins have been reported to promote the degradation of FBXW7 through auto-ubiquitination, such as Pin1, ERK, PLK2, CSN6, and LSD1 (9, 15, 34). In addition, some studies have indicated that FBXO45-MYCBP2 E3 ubiquitin ligase and Parkin ubiquitin ligase can directly ubiquitinate and degrade FBXW7 (35). In this study, we demonstrated that FBXO9 directly interacted with FBXW7, which resulted in the ubiquitination and proteasome-mediated degradation of FBXW7. Furthermore, FBXO9 exerts a tumor-promoting effect in HCC by downregulating FBXW7, thereby increasing the protein levels of FBXW7 oncogenic substrates, such as mTOR. Therefore, through our work, we uncover the mechanism by which FBXO9 exerts a tumor-promoting effect in HCC and propose a novel FBXO9-involved degradation mechanism of FBXW7. In future studies, we will continue to examine how FBXO9 causes the ubiquitination and degradation of FBXW7.

Chemotherapeutic treatment is an effective form of cancer therapy. However, primary and acquired drug resistance contribute to poor prognosis in cancer patients (12, 26). Recently, F-box protein has been considered to play a pivotal role in the development of drug resistance (12), and previous studies have indicated that FBXW7 increases the chemosensitivity of HCC (14, 31). Therefore, we investigated the effect of FBXO9 knockdown on drug resistance in HCC. The knockdown of FBXO9 impaired the resistance of HCC cells to lenvatinib and sorafenib. These results imply that FBXO9-targeted therapy is synergistic with chemotherapy; thus, FBXO9 inhibitors may be valuable for the treatment of patients with HCC in the future.

Another interesting finding of the present study is that we identified ZNF143 as a transcription factor of FBXO9. Multiple studies have reported that ZNF143 has cancer-promoting capabilities in a variety of tumors (23, 24, 36). Our previous study showed that ZNF143 stimulates proliferation *via* the ZNF143–MDIG–CDC6 regulatory axis in HCC cells (21). Here, we demonstrated that ZNF143 could exert oncogenic functions through the ZNF143-FBXO9-FBXW7 axis. These findings will deepen our understanding of the role of ZNF143 and FBXO9 in HCC.

In conclusion, we have presented a novel finding for the ZNF143–FBXO9–FBXW7 axis (**Figure 7C**). Under the control of ZNF143, FBXO9 facilitates HCC cell proliferation, metastasis, and drug resistance by interacting with FBXW7. To the best of our knowledge, this study is the first to reveal the carcinogenic effect of FBXO9 in HCC, which may provide a novel understanding of the functions of FBXO9 in HCC diagnosis and therapy.

## Data Availability Statement

The original contributions presented in the study are included in the article/**Supplementary Material**. Further inquiries can be directed to the corresponding author.

## Ethics Statement

The studies involving human participants were reviewed and approved by The Research Ethics Committee of Renji Hospital. The patients/participants provided their written informed consent to participate in this study.

## Author Contributions

ZW: Conceptualization, methodology, validation, formal analysis, investigation, writing – original draft. XC, LZ, XZ, CG, FZ, HT, HL: Investigation, methodology. TC, HX: Resources. JL: Funding acquisition, project administration, conceptualization, methodology, investigation, formal analysis, supervision, writing – review & editing. All authors read and approved the final manuscript. All authors contributed to the article and approved the submitted version.

## Funding

This study was supported, in part, by grants from the National Natural Science Foundation of China (82173331, 81972580, 81773152), the National Key Sci-Tech Special Project of China (2018ZX10723204-006) and the SKLORG Research foundation (zz-94-21-02).

## Conflict of Interest

The authors declare that the research was conducted in the absence of any commercial or financial relationships that could be construed as a potential conflict of interest.

## Publisher’s Note

All claims expressed in this article are solely those of the authors and do not necessarily represent those of their affiliated organizations, or those of the publisher, the editors and the reviewers. Any product that may be evaluated in this article, or claim that may be made by its manufacturer, is not guaranteed or endorsed by the publisher.
