# Supplementary material for: FBXO9 Mediates the Cancer-Promoting Effects of ZNF143 by Degrading FBXW7 and Facilitates Drug Resistance in Hepatocellular Carcinoma
Source: Front Oncol. 2022 Jun 30;12:930220. doi: 10.3389/fonc.2022.930220 (PMC9280481; doi:10.3389/fonc.2022.930220)
Supplement: Supplementary file 1 [file DataSheet_1.docx]

Supplementary Material

**Supplementary tables (Table S1-S5)**

| **Supplementary Table S1. Clinical characteristics of tissue donors** | |
| --- | --- |
| **Characteristic** | **No. of Patients (n = 64)** |
| **Gender** |  |
| Male | 43 (67.2%) |
| Female | 21 (32.8%) |
| **Age (years)** |  |
| ≤ 60 | 28 (43.8%) |
| > 60 | 36 (56.2%) |
| **HBV** |  |
| Negative | 26 (40.6%) |
| Positive | 38 (59.4%) |
| **Cirrhosis** |  |
| Negative | 36 (56.2%) |
| Positive | 28 (43.8%) |
| **AFP (ng/ml)** |  |
| ≤ 20 | 29 (45.3%) |
| > 20 | 35 (54.7%) |
| **Main Tumor size (cm)** |  |
| < 3 | 15 (23.4%) |
| 3-5 | 31 (48.4%) |
| > 5 | 18 (28.2%) |
| **Tumor number** |  |
| Single | 60 (93.7%) |
| Multiple | 4 (6.3%) |
| **TNM stage** |  |
| I | 7 (10.9%) |
| II | 45 (70.3%) |
| III | 8 (12.5%) |
| IV | 4 (6.3%) |
| **Edmondson grade** |  |
| I | 3 (4.7%) |
| II | 40 (62.5%) |
| III | 20 (31.3%) |
| IV | 1 (1.5%) |

| **Supplementary Table S2. Details of shRNA target sequences.** | |
| --- | --- |
| Name | Target sequence |
| shZNF143#1 | CACTCTGTTGCTATGGTTA |
| shZNF143#2 | ACACTCATTCCAAACCTTA |
| shFBXO9#1 | GGATACATCATTCTTGTCACA |
| shFBXO9#2 | GGAAACAGCTACATTGAAGAT |
| shNC | TTCTCCGAACGTGTCACGT |

| **Supplementary Table S3. Primers of qPCR used in this study.** | | |
| --- | --- | --- |
| Name | Forward (5’-3’) | Reverse (5’-3’) |
| ZNF143 | CGCAGTCTGACACCATCTTG | CCAATCATTCCAGTACCTGCT |
| FBXO9 | CTCAGTGGATGTTTGAACTTGCT | CCTTTGGTATCTGCCGATGTTTT |
| GAPDH | AGAAGGCTGGGGCTCATTTG | AGGGGCCATCCACAGTCTTC |
| **ChIP-qPCR** | | |
| Primer#1 | GTCTCCACGGCACTTGGGTTTC | GGAGGAAAGAGCCAACGCAAGG |
| Primer#2 | ACCCTTAGCCCTCCACTAGGCA | AGCGTCAGCCCTACTGGAGA |

| **Supplementary Table S4. The antibodies for western blot used in this study** | | | |
| --- | --- | --- | --- |
| Antibody | Catalogue number | Dilution | Company |
| ZNF143 | 16618-1-AP | 1:750 | Proteintech |
| FBXO9 | 11161-1-AP | 1:500 | Proteintech |
| CDC4(FBXW7) | sc-293423 | 1:150 | Santa Cruz |
| mTOR | sc-517464 | 1:200 | Santa Cruz |
| p-mTOR | 5536 | 1:1000 | CST |
| S6K | 9202 | 1:750 | CST |
| p-S6K | 9234 | 1:750 | CST |
| Akt | 4691 | 1:750 | CST |
| p-Akt | 3787 | 1:750 | CST |
| Ubiquitin | 3933 | 1:1000 | CST |
| β-actin | A1978 | 1:10000 | Abcam |
| HRP-anti-Rabbit IgG | A0545 | 1:5000 | Abcam |
| HRP-anti-Mouse IgG | A4416 | 1:5000 | Abcam |

| **Supplementary Table S5. The antibodies for immunoprecipitation used in this study.** | | | |
| --- | --- | --- | --- |
| Antibody | Catalogue number | Dilution | Validation |
| **ChIP** | | | |
| ZNF143 | 16618-1-AP | 1μg | Proteintech |
| **Co-IP** | | | |
| FBXO9 | 11161-1-AP | 1μg/250μg protein | Proteintech |
| CDC4(FBXW7) | sc-293423 | 1μg/250μg protein | Santa Cruz |
| mTOR | sc-517464 | 1μg/250μg protein | Santa Cruz |

**Supplementary figures (Figure S1-S9)**


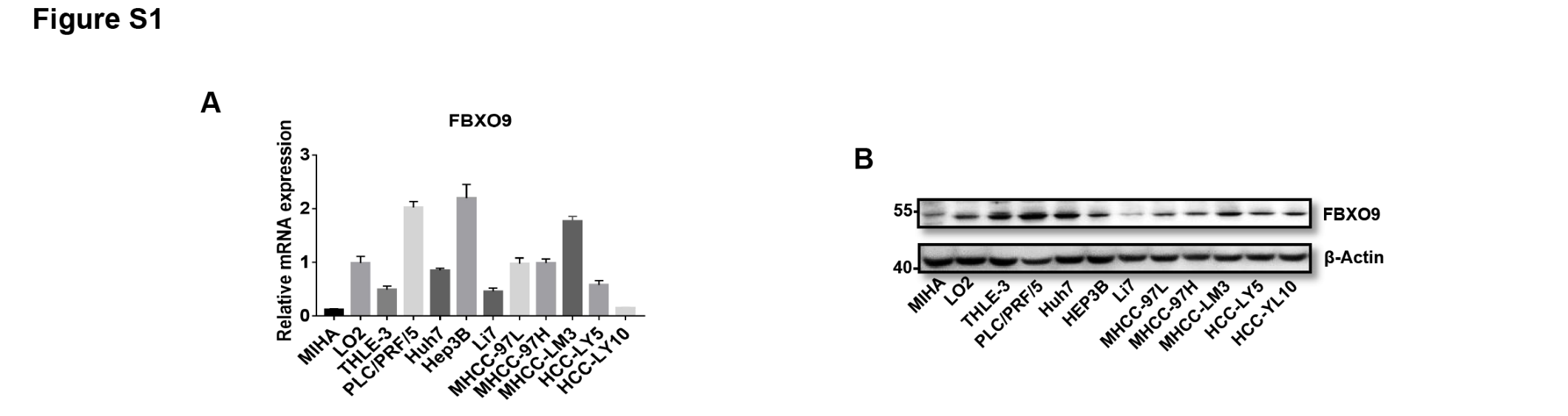


**Supplementary Figure S1.** The expression of FBXO9 was detected by qPCR (A) and Western blotting (B) in a series of HCC cell lines and immortalized liver cell lines. Error bars represent mean ± S.D; n = 3.


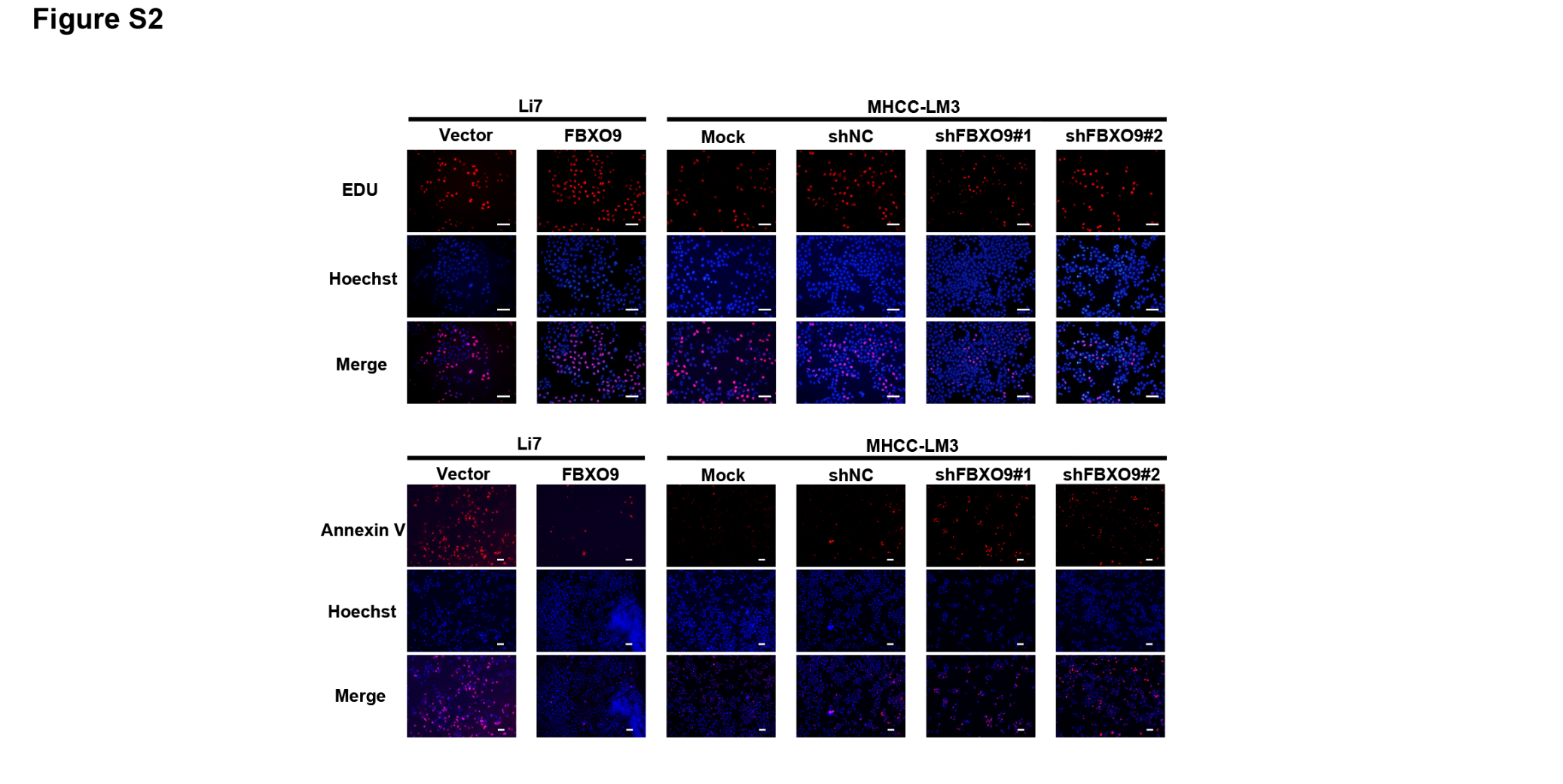


**Supplementary Figure S2.** Staining of cell proliferation and apoptosis. EdU incorporation experiment and the Annexin V staining was used to analyze the cell proliferation and apoptosis of Li7 cells with FBXO9 overexpression and MHCC-LM3 cells after silencing FBXO9 respectively. Scale bars: 100 μm.


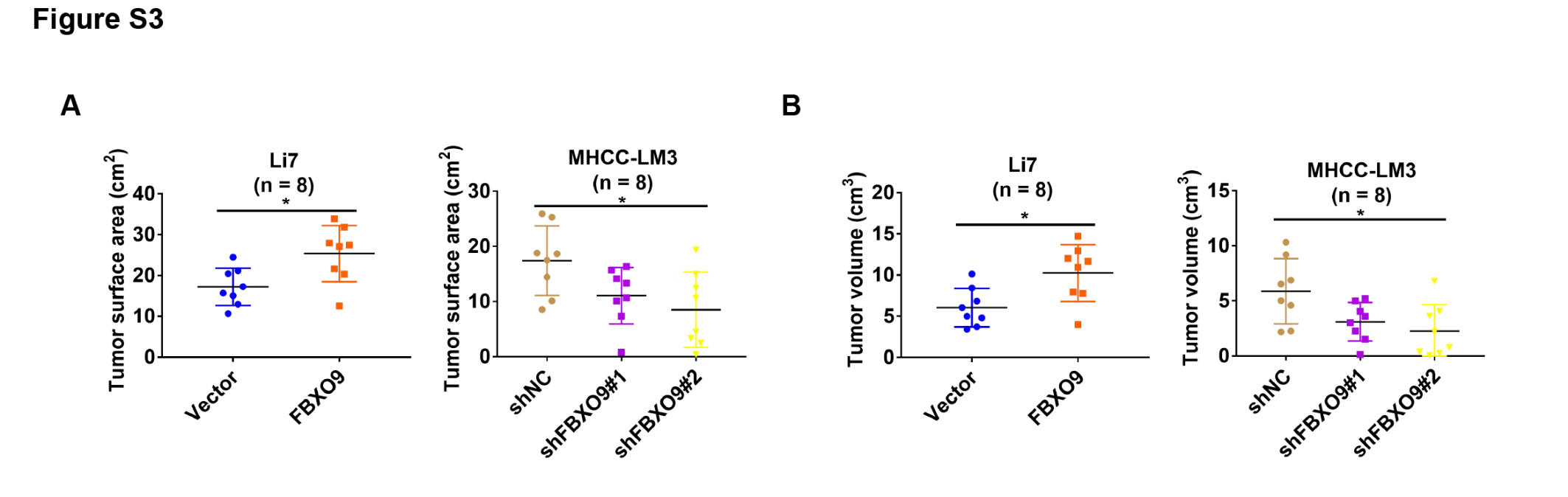


**Supplementary Figure S3.** Quantitative analysis of tumor surface area (A) and tumor volume (B) of orthotopic xenograft mouse inoculated with FBXO9 overexpressed Li7 and knockdown MHCC-LM3 cell lines (n = 8).


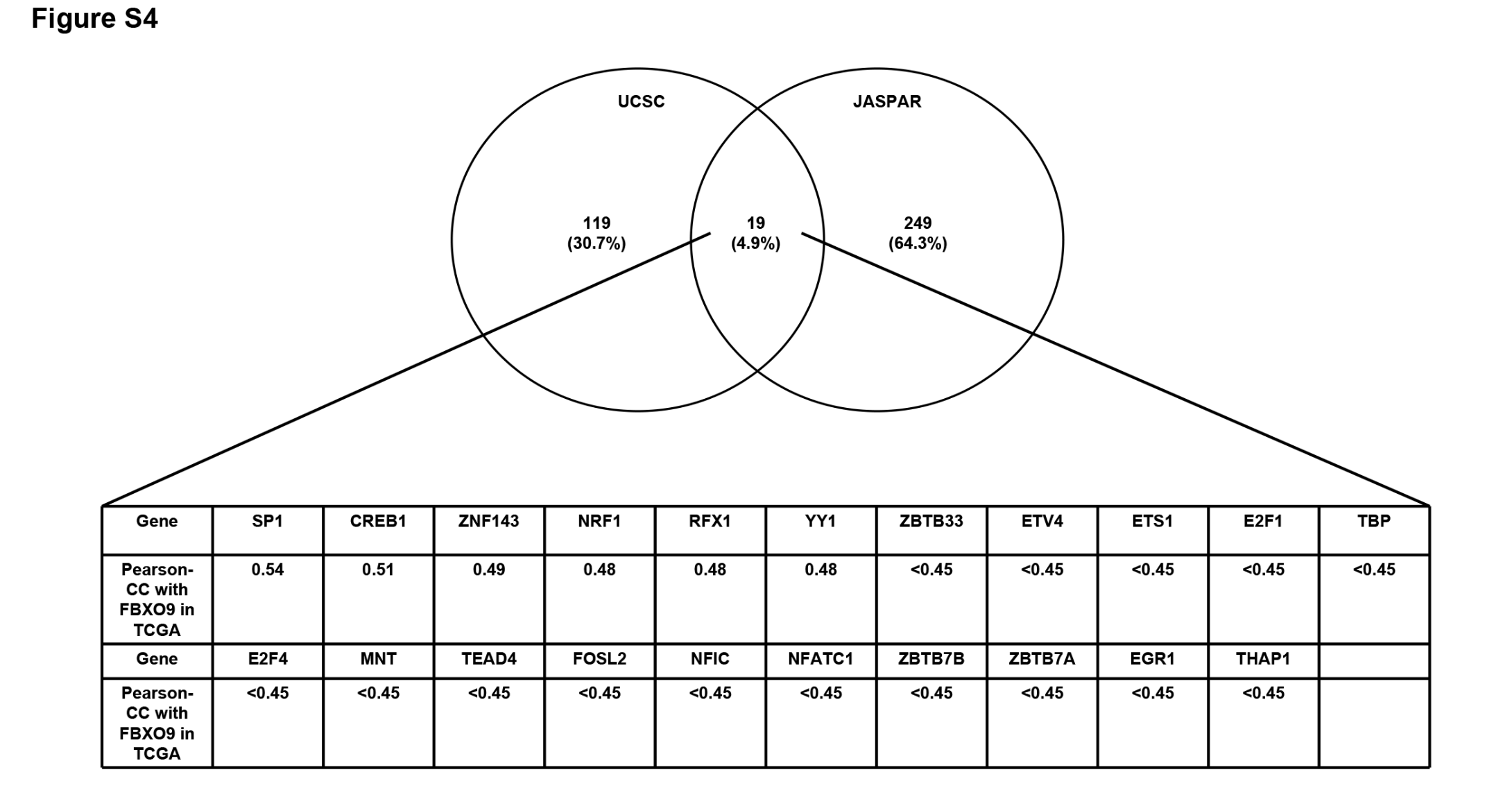


**Supplementary Figure S4.** The potential transcription factors of FBXO9. There are 19 common transcription factors predicted by UCSC and JASPAR database, and only 6 transcription factors (SP1, CREB1, ZNF143, NRF1, RFX1, YY1) which have a Pearson correlation coefficient (Pearson-CC) greater than 0.45 with FBXO9 in TCGA.


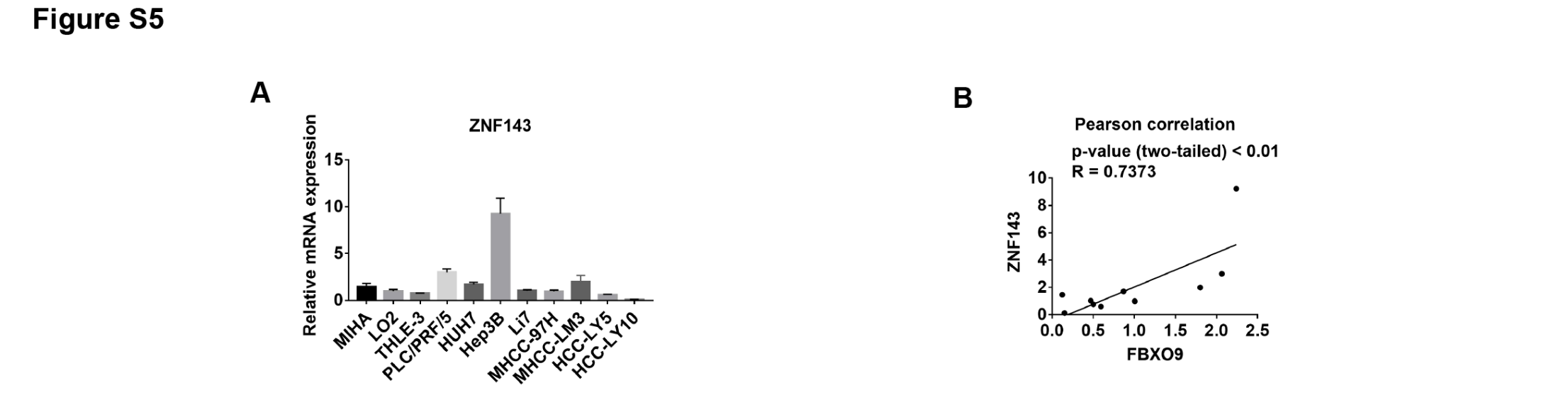


**Supplementary Figure S5. (A)** The expression of ZNF143 was detected by qPCR in a series of HCC cell lines and immortalized liver cell lines. **(B)** The expression correlation between FBXO9 and ZNF143 in these cells was analyzed by qPCR. Error bars represent mean ± S.D; n = 3. R, Pearson correlation coefficient.


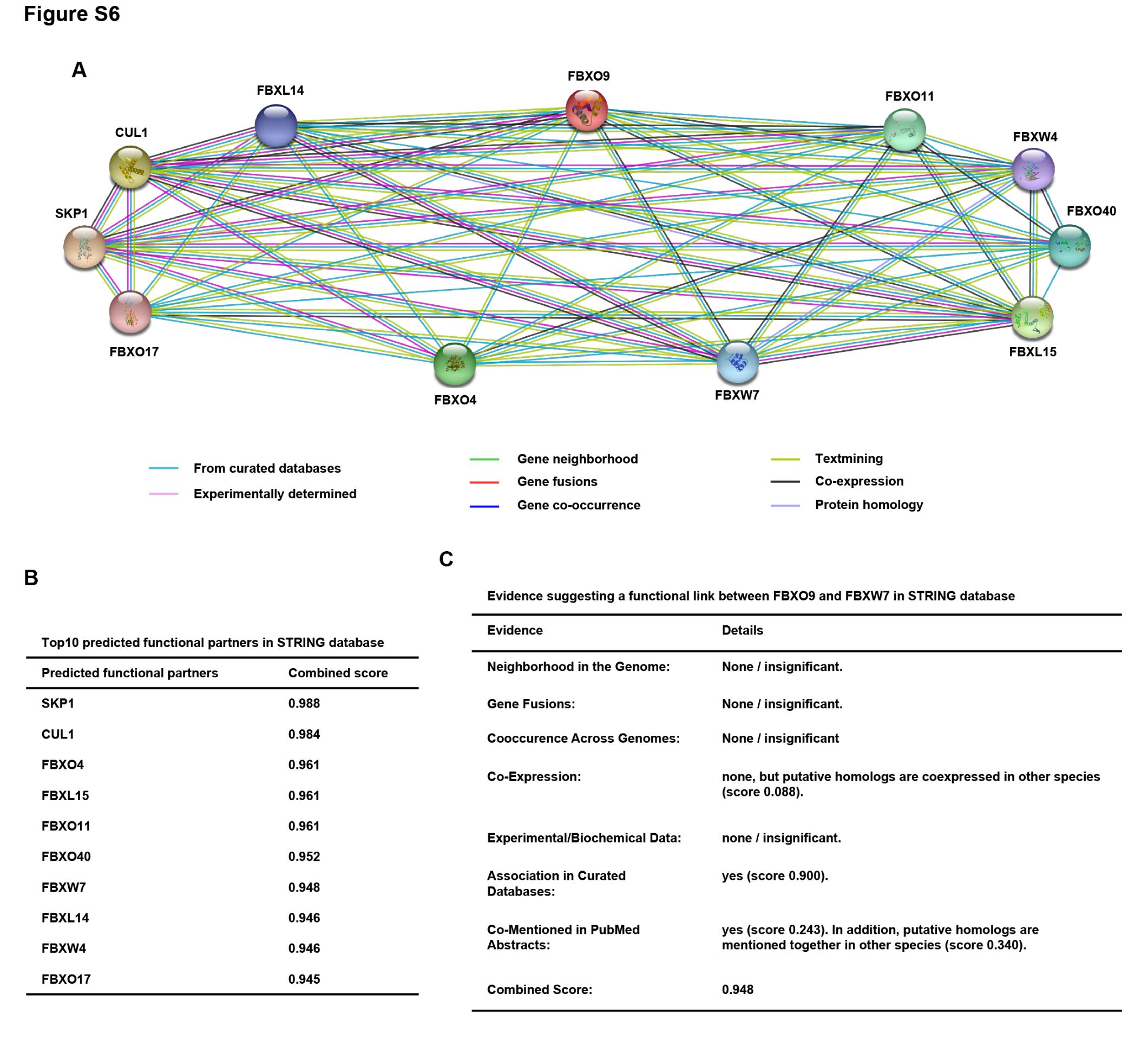


**Supplementary Figure S6. (A-B)** Top10 possible functional partners of FBXO9 predicted by using the STRING database (version 11.0b). **(C)** Evidence suggesting a functional link between FBXO9 and FBXW7 in STRING database.


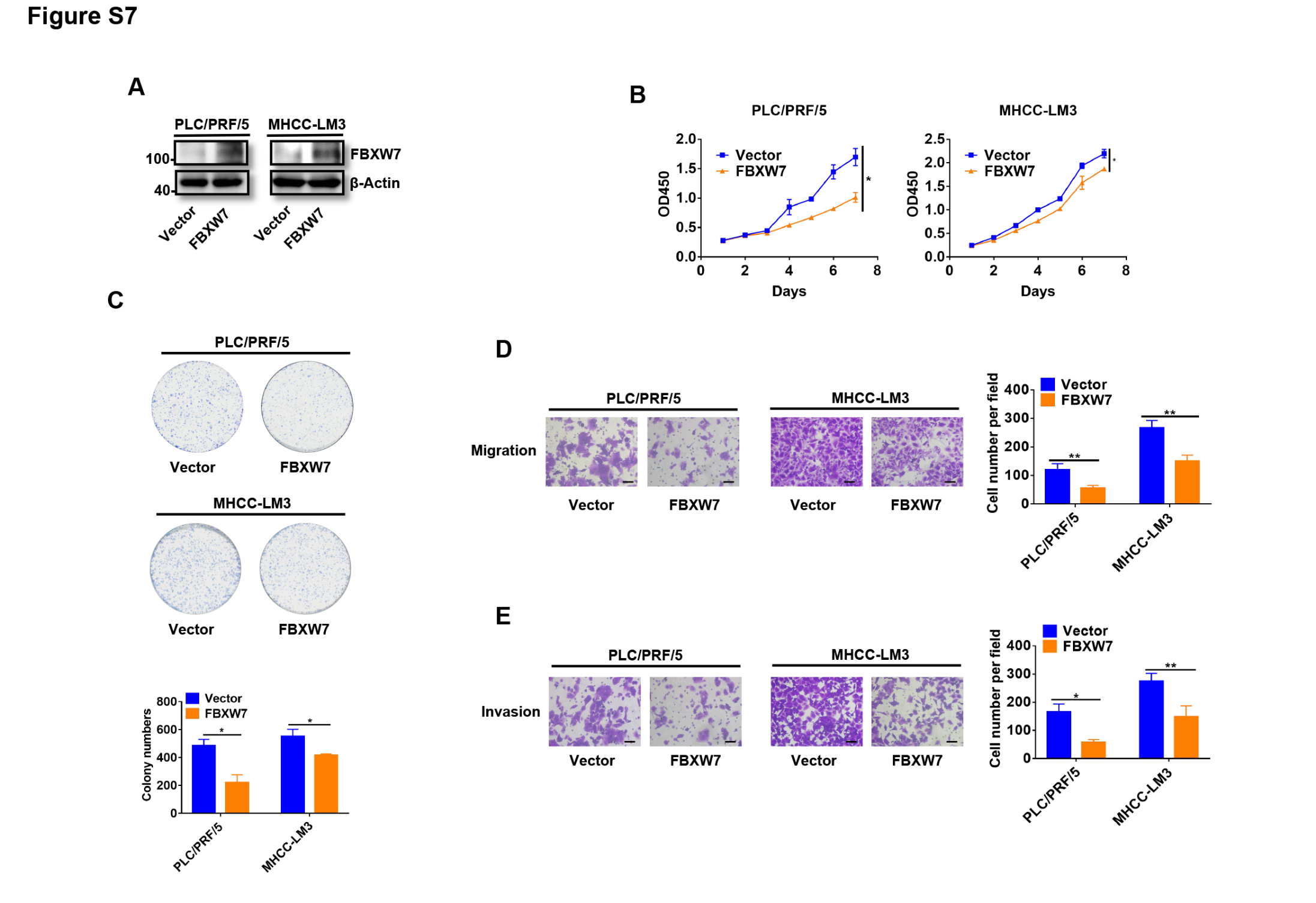


**Supplementary Figure S7.** FBXW7 overexpression can inhibit the progression of HCC. **(A)** Western blot assay of the FBXW7 expression in FBXW7-overexpressing HCC cells. **(B-C)** The proliferation capacity of FBXW7 overexpressed HCC cells was analyzed by the CCK-8 and colony formation assays. **(D-E)** The mobility capacity of FBXO9 overexpressed HCC cells was analyzed by the transwell assays. Data are shown as mean ± S.D, n = 3. Scale bars: 100 μm. *, P < 0.05; **, P < 0.01, by two-tailed Student’s t-test or one-way ANOVA.


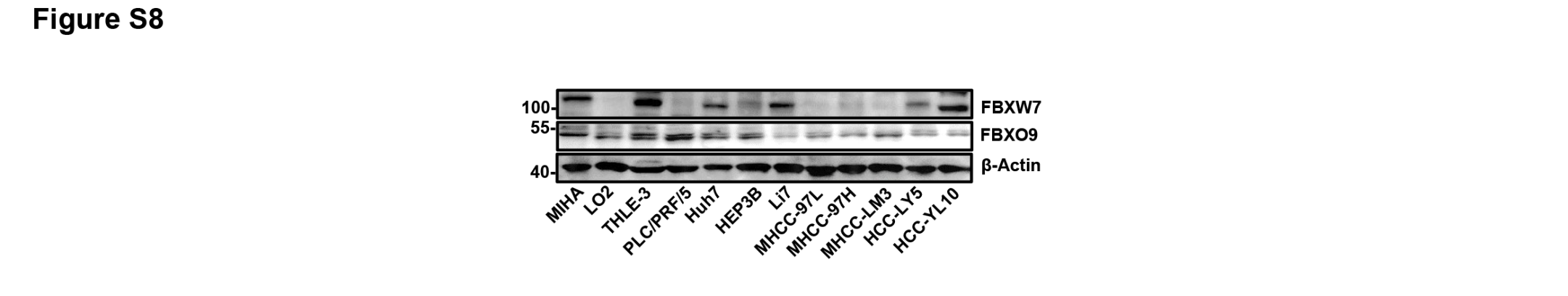


**Supplementary Figure S8.** The protein level of FBXO9 and FBXW7 was detected by Western blotting in a series of HCC cell lines and immortalized liver cell lines.


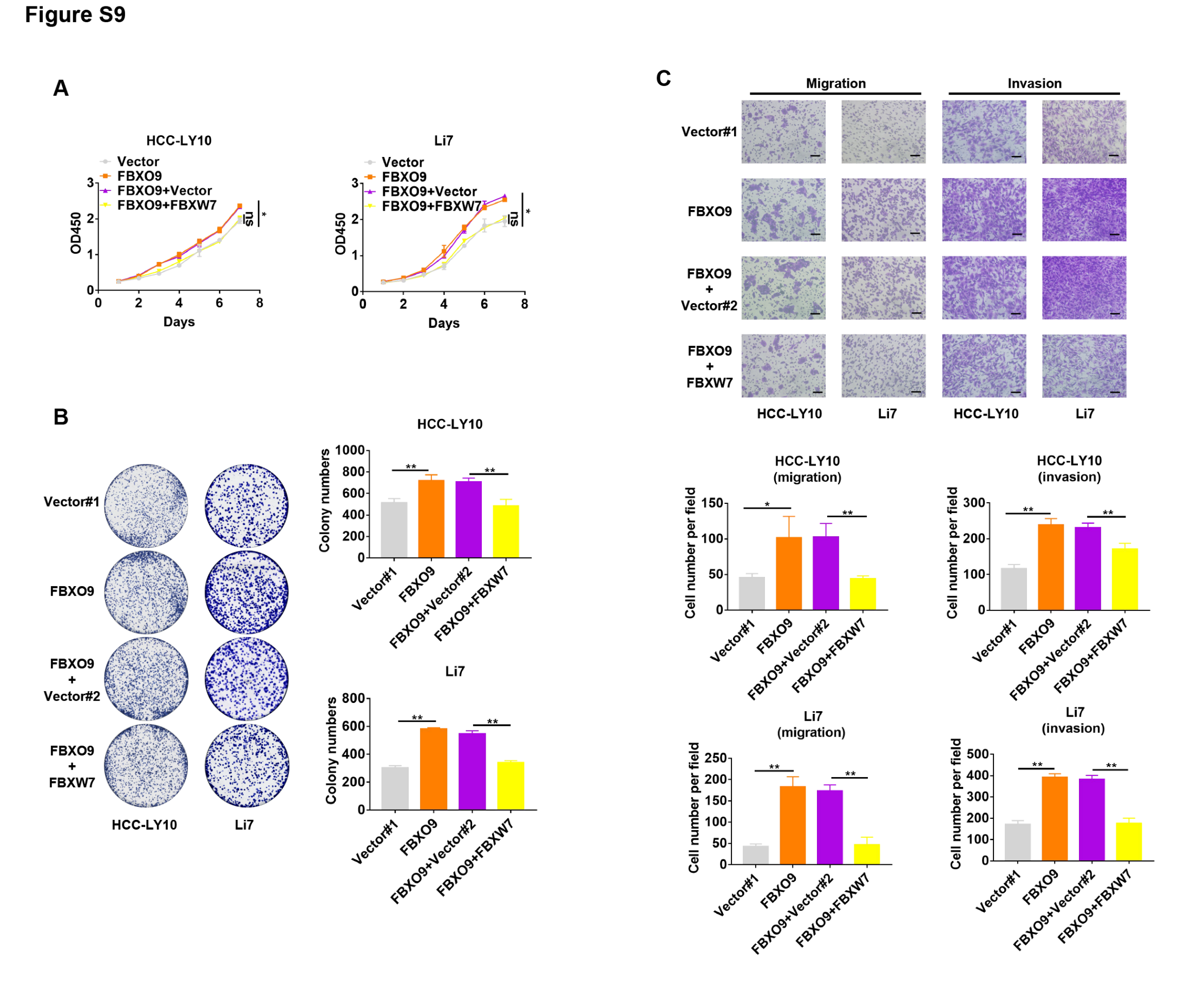


**Supplementary Figure S9.** FBXW7 overexpression rescued FBXO9 overexpression-induced cell proliferation, migration, and invasion translocation in HCC cells. (**A–B)** CCK-8 and colony formation assays were used to determine the proliferation of FBXO9-overexpressing HCC cells after FBXW7 overexpression. Quantitative analysis is shown in bar graphs. **(C)** Transwell assays showed that overexpressing FBXW7 reversed the influence on migration and invasion of HCC cells induced by FBXO9 overexpression. Error bars are shown as the mean ± SD, n = 3. Scale bars: 100 μm.*, P < 0.05; **, P < 0.01; ns, no significance by two-tailed Student *t*-test or one-way ANOVA.


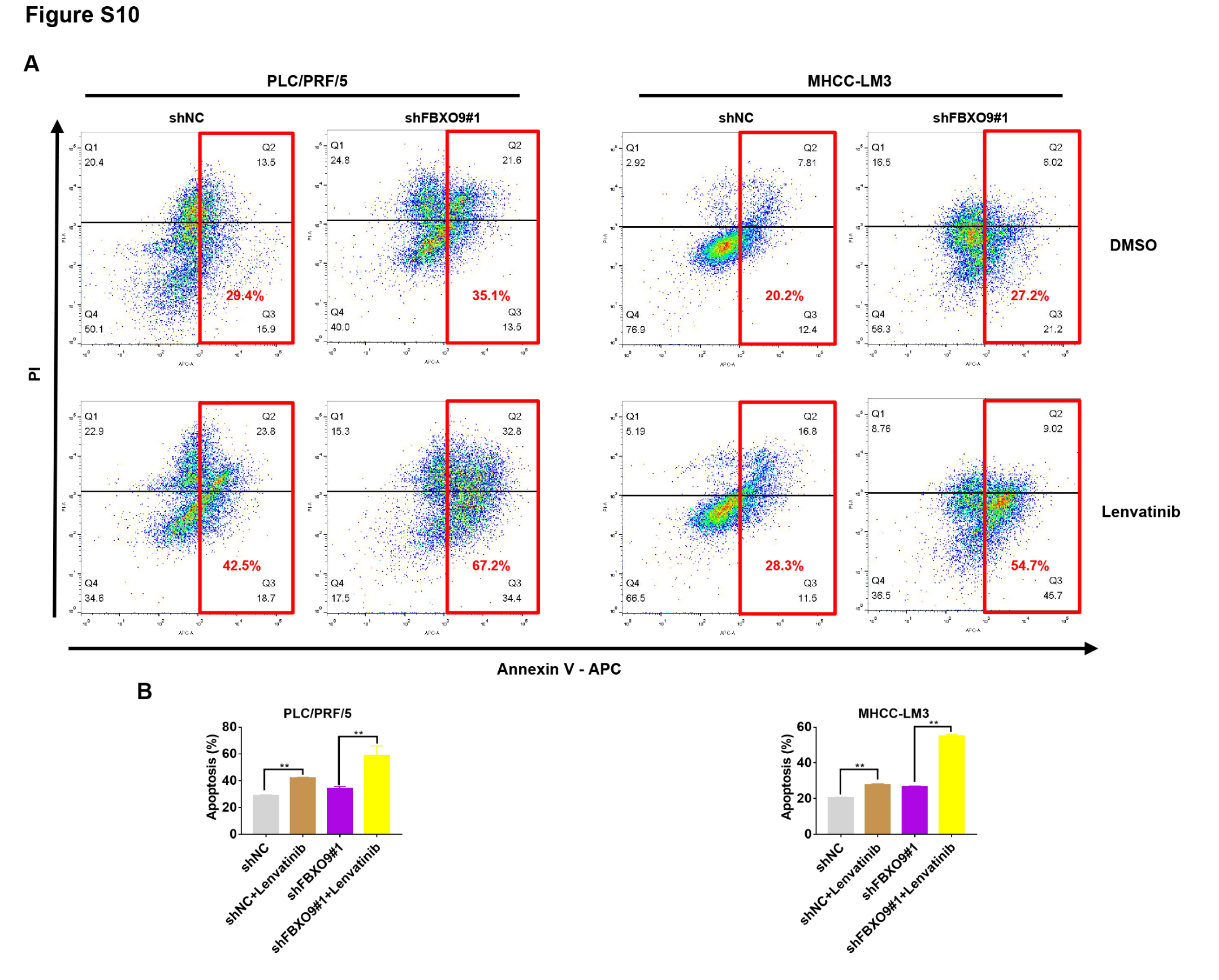


**Supplementary Figure S10. (A-B)** Lenvatinib (10 μM, 24 h) treatment induced apoptosis more obviously upon knockdown of FBXO9.
