# Supplementary material for: FBXO9 Mediates the Cancer-Promoting Effects of ZNF143 by Degrading FBXW7 and Facilitates Drug Resistance in Hepatocellular Carcinoma
Source: Front Oncol. 2022 Jun 30;12:930220. doi: 10.3389/fonc.2022.930220 (PMC9280481; doi:10.3389/fonc.2022.930220)

FBXO9 mediates the cancer-promoting effects of ZNF143 by degrading FBXW7 and facilitates drug resistance in hepatocellular carcinoma

Raw clone formation

Figure 2D

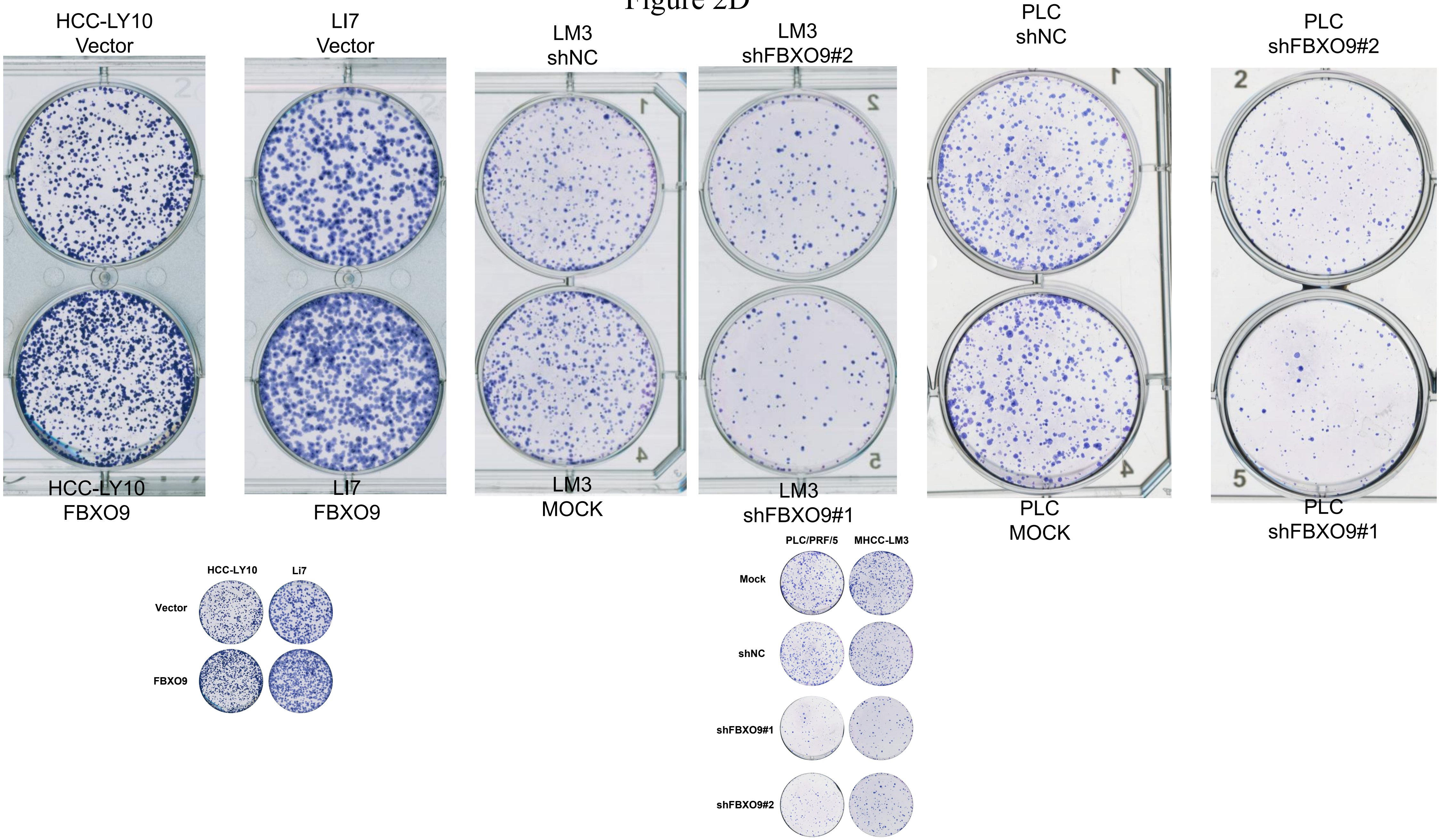
HCC-LY10
Vector
LI7
Vector
LM3
shNC
LM3
shFBXO9#2
PLC
shNC
PLC
shFBXO9#2
HCC-LY10
FBXO9
LI7
FBXO9
LM3
MOCK
LM3
shFBXO9#1
PLC
MOCK
PLC
shFBXO9#1
HCC-LY10
Li7
Vector
FBXO9
PLC/PRF/5
MHCC-LM3
Mock
shNC
shFBXO9#1
shFBXO9#2

Figure 4C

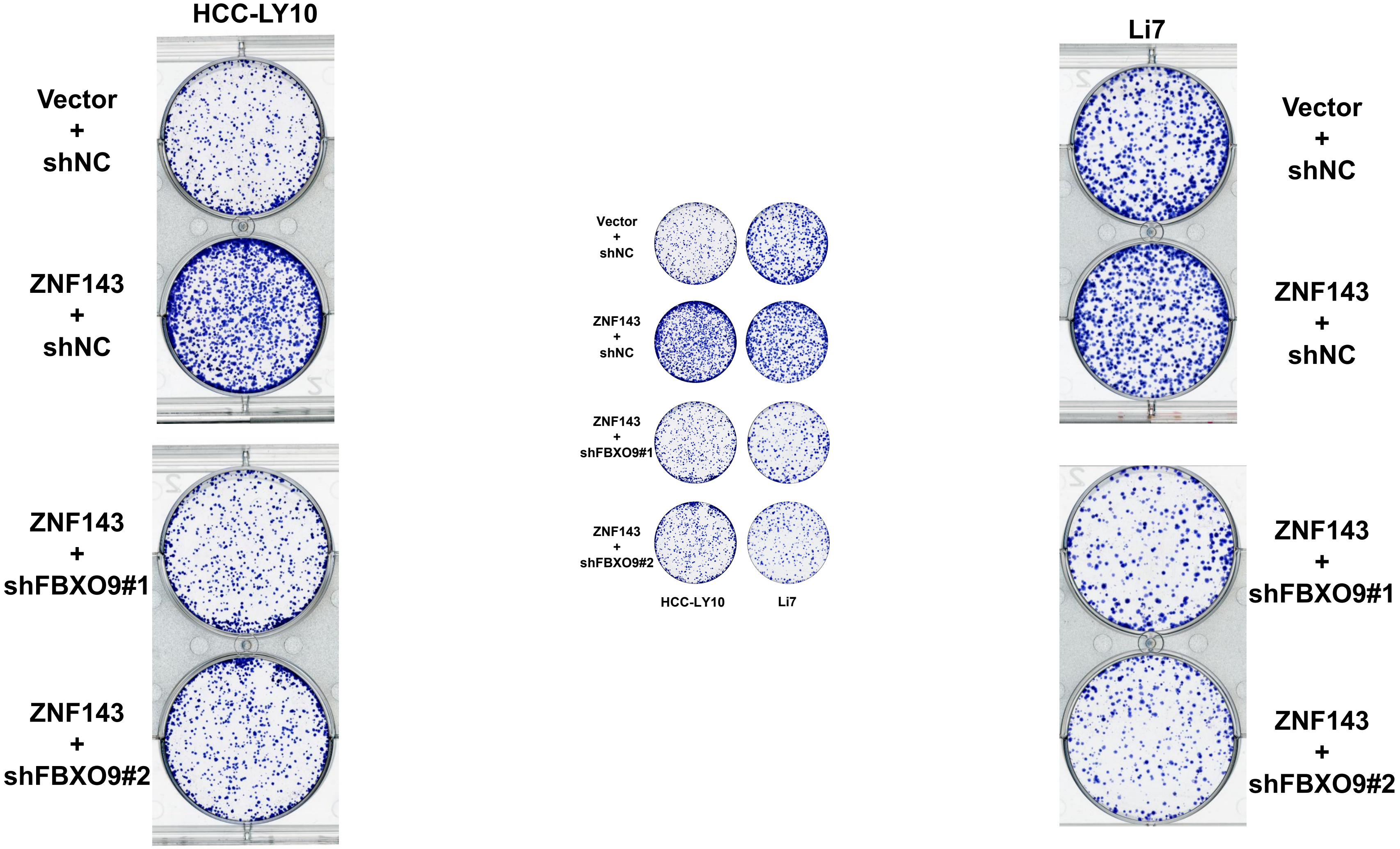
HCC-LY10
Vector + shNC
ZNF143 + shNC
ZNF143 + shFBXO9#1
ZNF143 + shFBXO9#2
Vector + shNC
ZNF143 + shNC
ZNF143 + shFBXO9#1
ZNF143 + shFBXO9#2
HCC-LY10
Li7
Li7
Vector + shNC
ZNF143 + shNC
ZNF143 + shFBXO9#1
ZNF143 + shFBXO9#2

Figure 4G

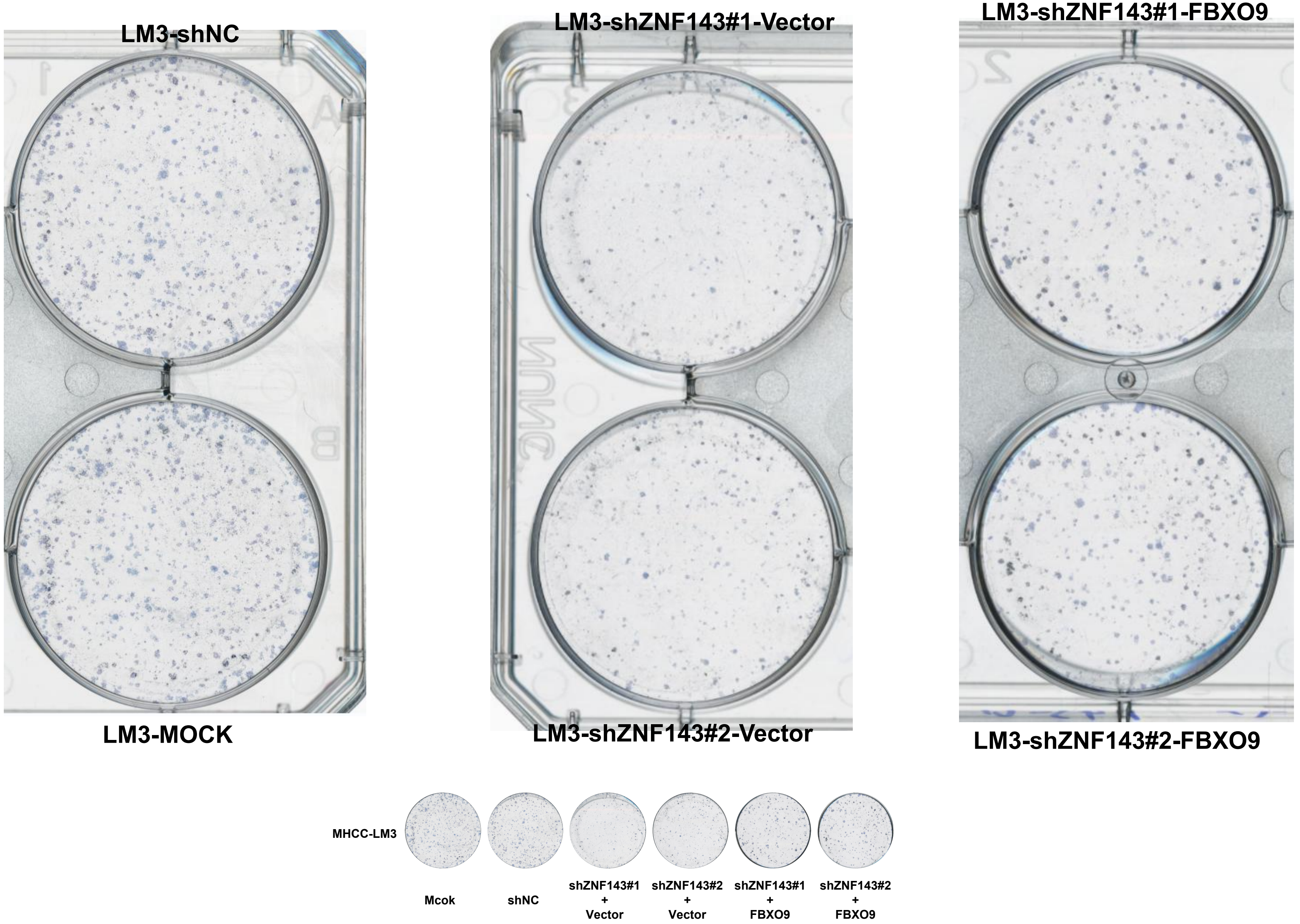
LM3-shNC
LM3-MOCK
LM3-shZNF143#1-Vector
LM3-shZNF143#2-Vector
LM3-shZNF143#1-FBXO9
LM3-shZNF143#2-FBXO9
MHCC-LM3
Mcok
shNC
shZNF143#1 + Vector
shZNF143#2 + Vector
shZNF143#1 + FBXO9
shZNF143#2 + FBXO9

Figure S7C

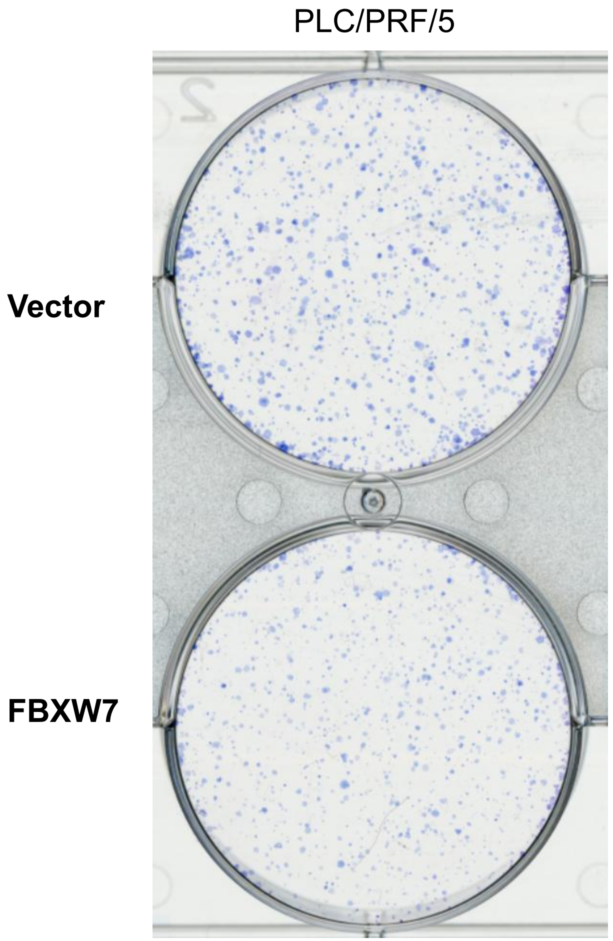
PLC/PRF/5
Vector
FBXW7

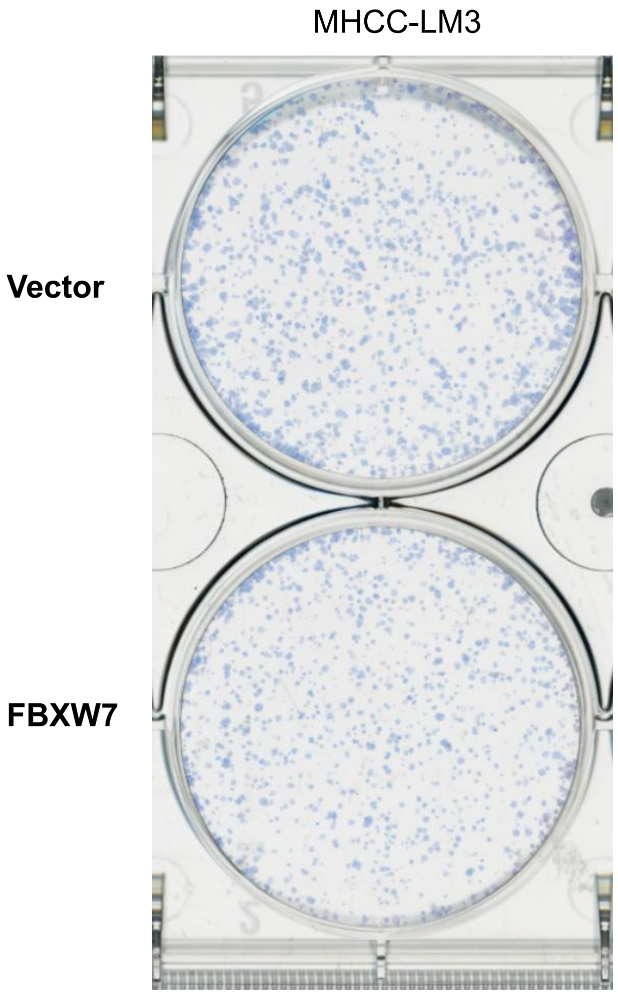
MHCC-LM3
Vector
FBXW7

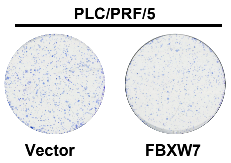
PLC/PRF/5
Vector
FBXW7

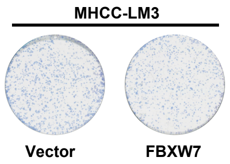
MHCC-LM3
Vector
FBXW7

Figure S9B

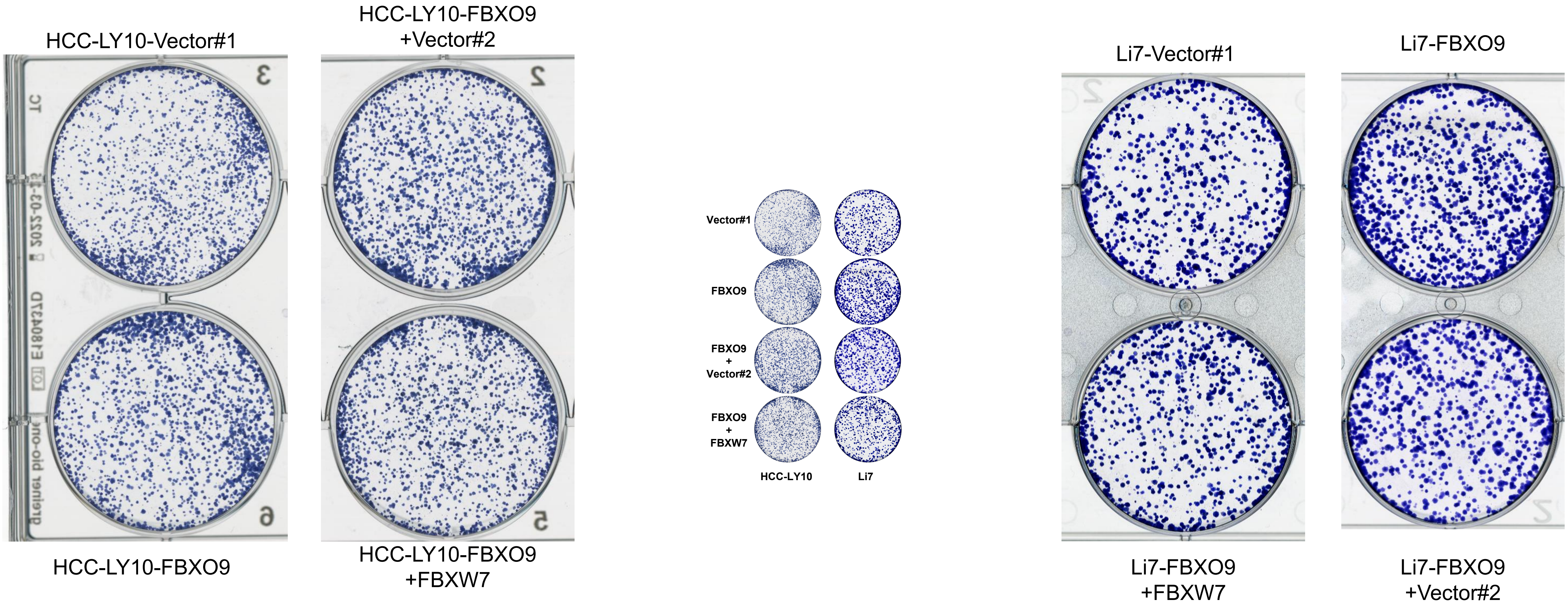
HCC-LY10-Vector#1
HCC-LY10-FBXO9
+Vector#2
HCC-LY10-FBXO9
HCC-LY10-FBXO9
+FBXW7
Vector#1
FBXO9
FBXO9
+
Vector#2
FBXO9
+
FBXW7
HCC-LY10
Li7
Li7-Vector#1
Li7-FBXO9
Li7-FBXO9
+FBXW7
Li7-FBXO9
+Vector#2

Figure 6B
MHCC-LM3

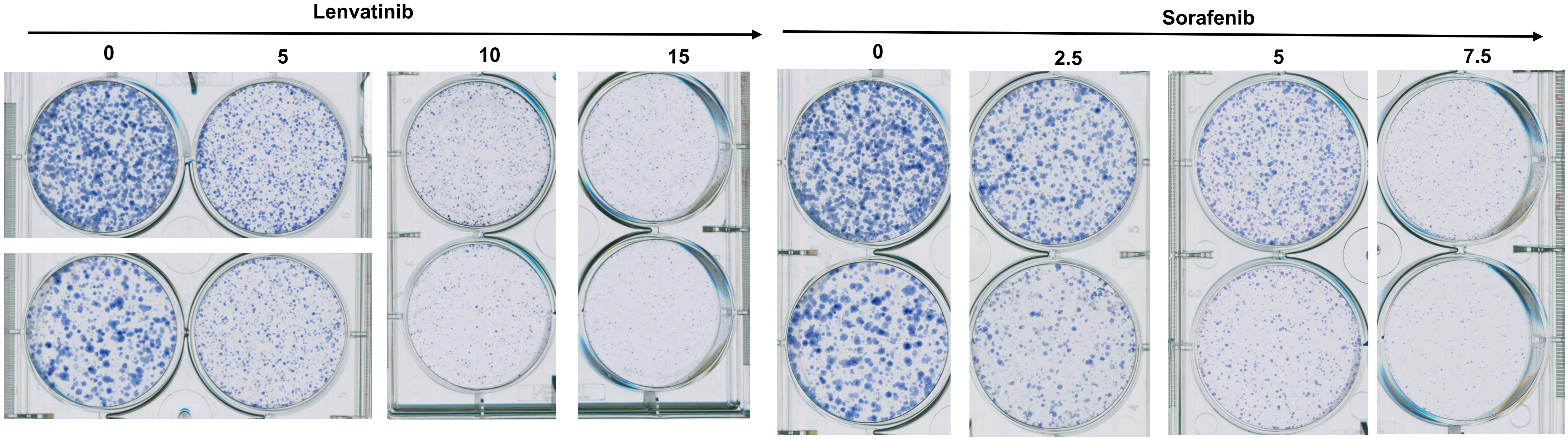

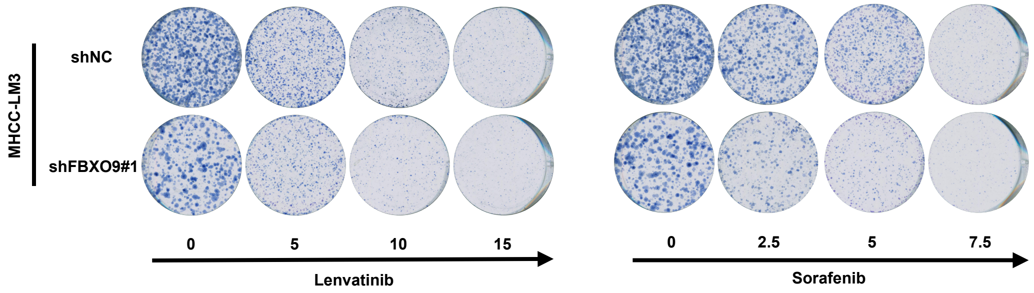

Figure 6B
PLC/PRF/5

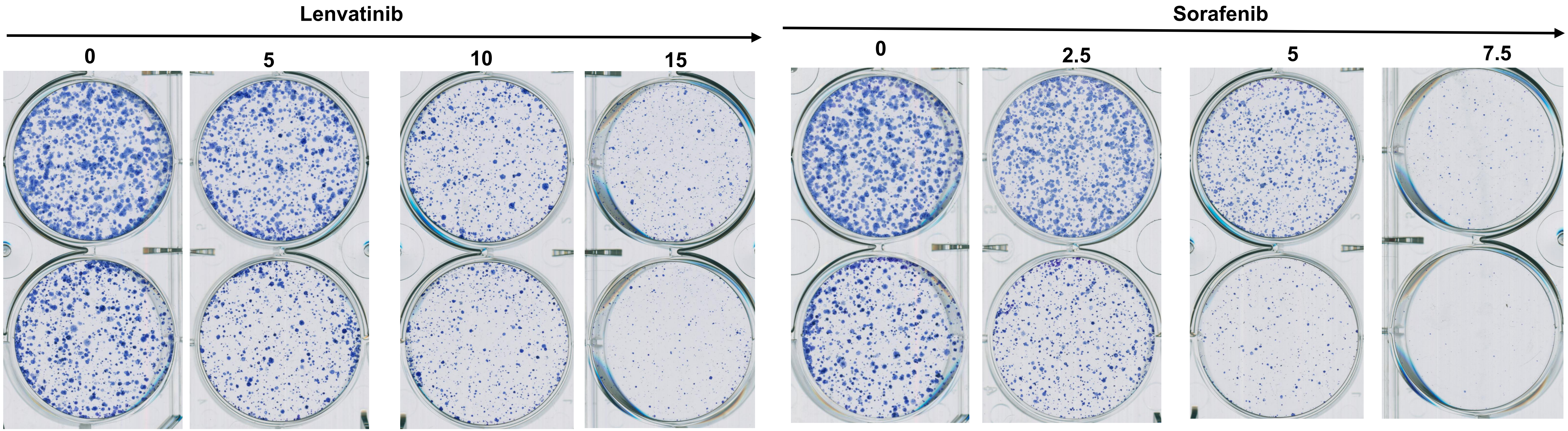

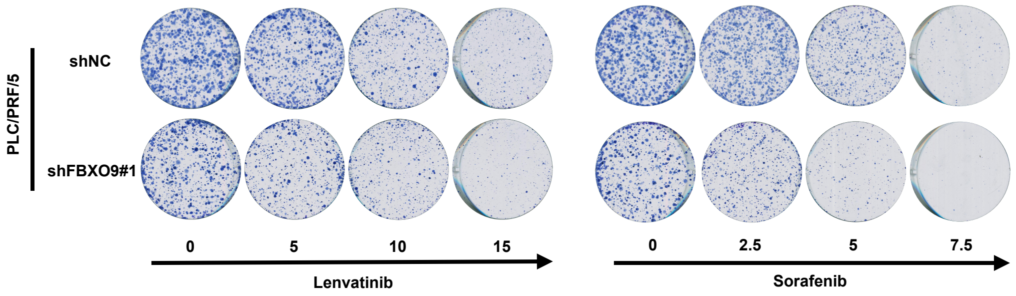

Supplement: Supplementary file 2 [file DataSheet_2.pdf]
