# Supplementary figures and images for: FBXO9 Mediates the Cancer-Promoting Effects of ZNF143 by Degrading FBXW7 and Facilitates Drug Resistance in Hepatocellular Carcinoma
Source: Front Oncol. 2022 Jun 30;12:930220. doi: 10.3389/fonc.2022.930220 (PMC9280481; doi:10.3389/fonc.2022.930220)

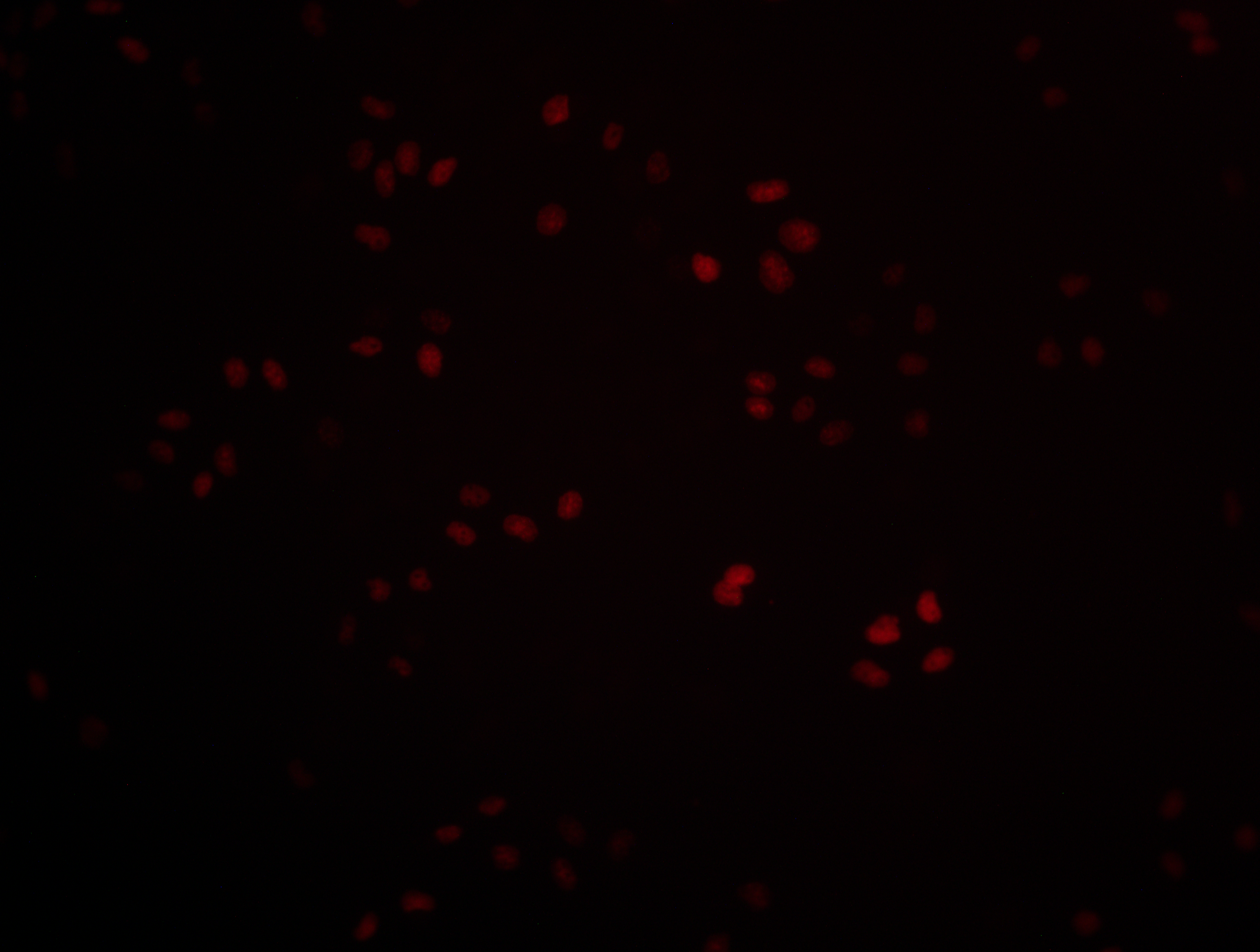

Supplement: Supplementary file 4 [file DataSheet_4.zip › Micrograph Figure S2 Li7 edu/Micrograph Figure S2-LI7 vector EDU.png]

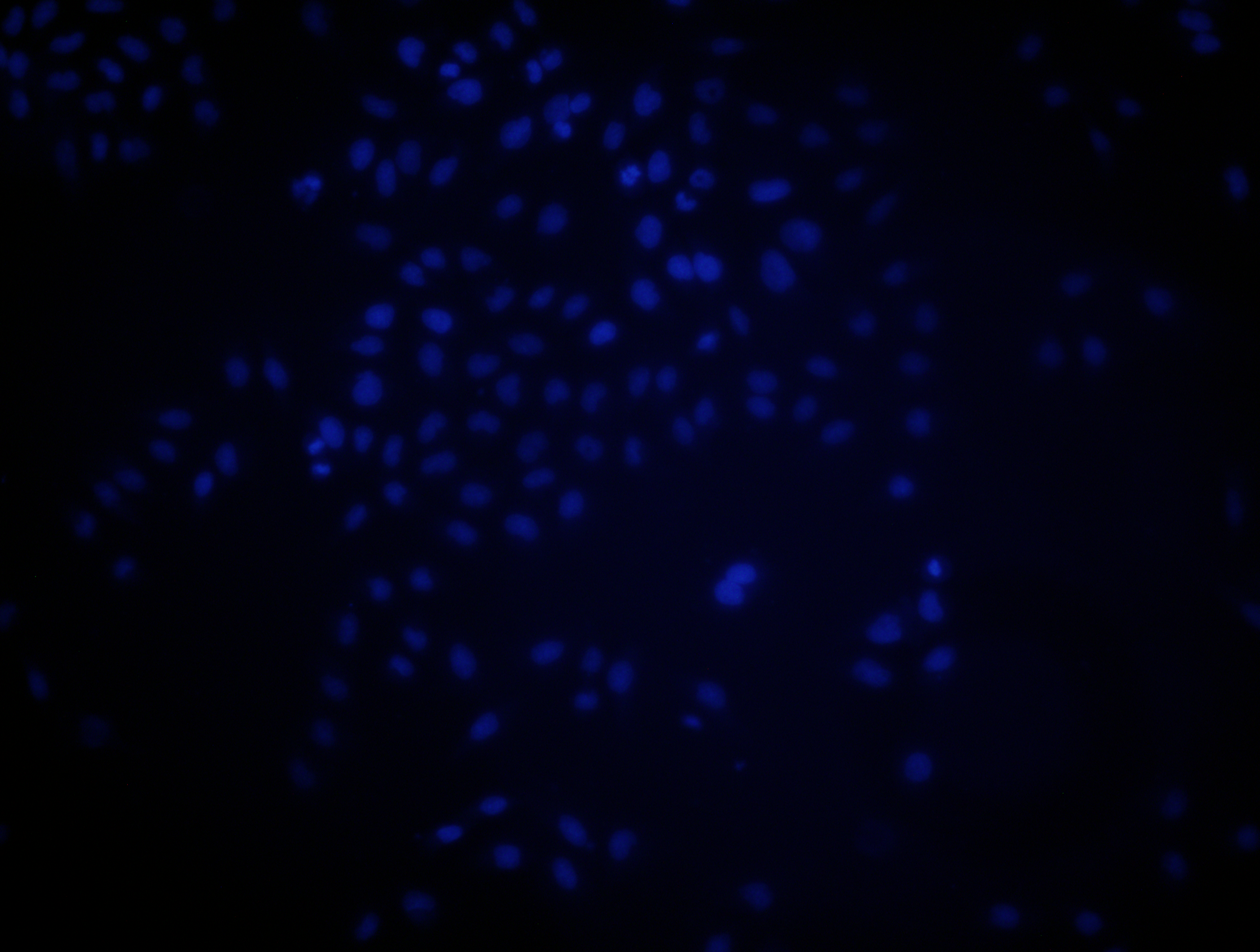

Supplement: Supplementary file 4 [file DataSheet_4.zip › Micrograph Figure S2 Li7 edu/Micrograph Figure S2-LI7 vector hoechst.png]

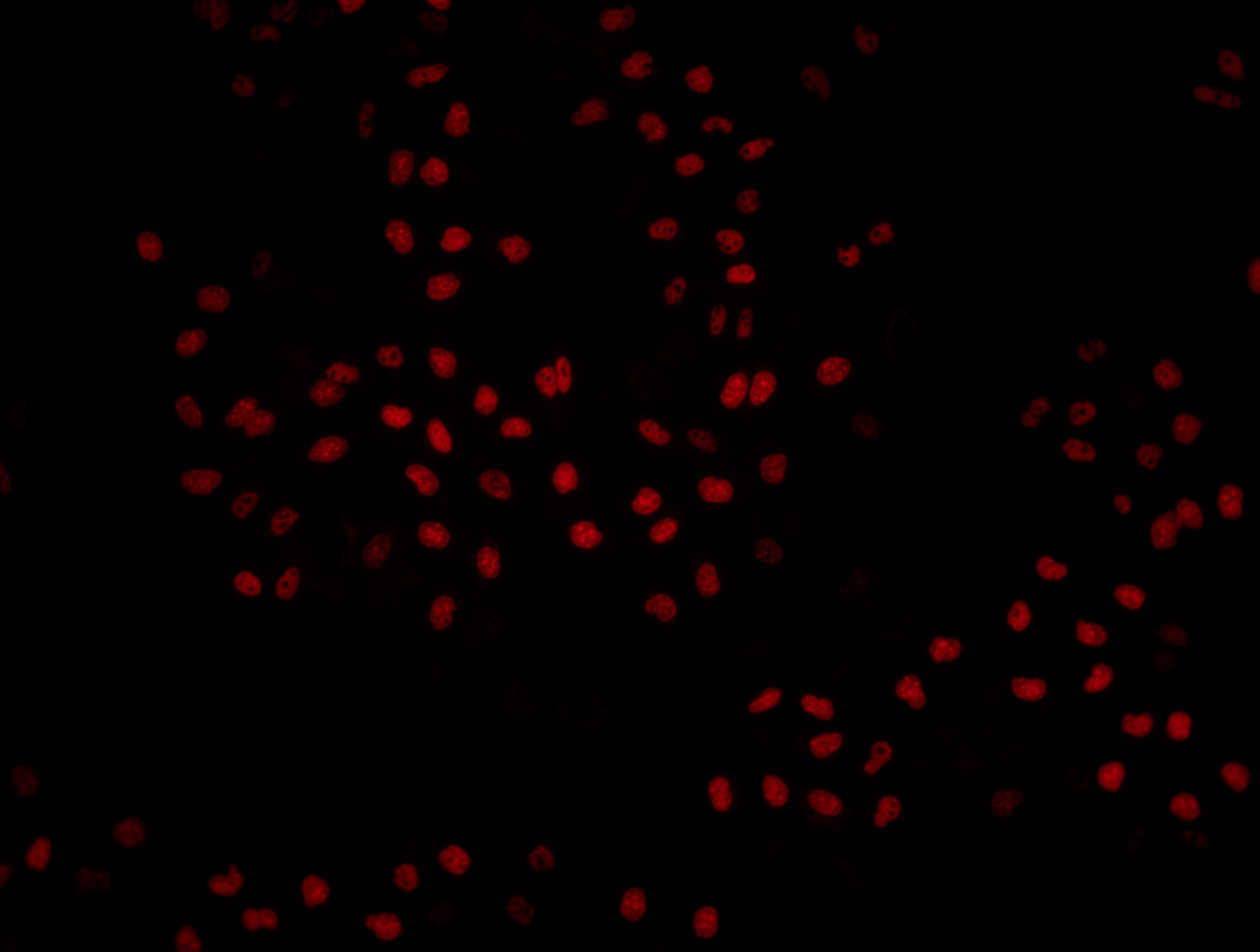

Supplement: Supplementary file 4 [file DataSheet_4.zip › Micrograph Figure S2 Li7 edu/Micrograph Figure S2-li7 fbxo9 edu.png]

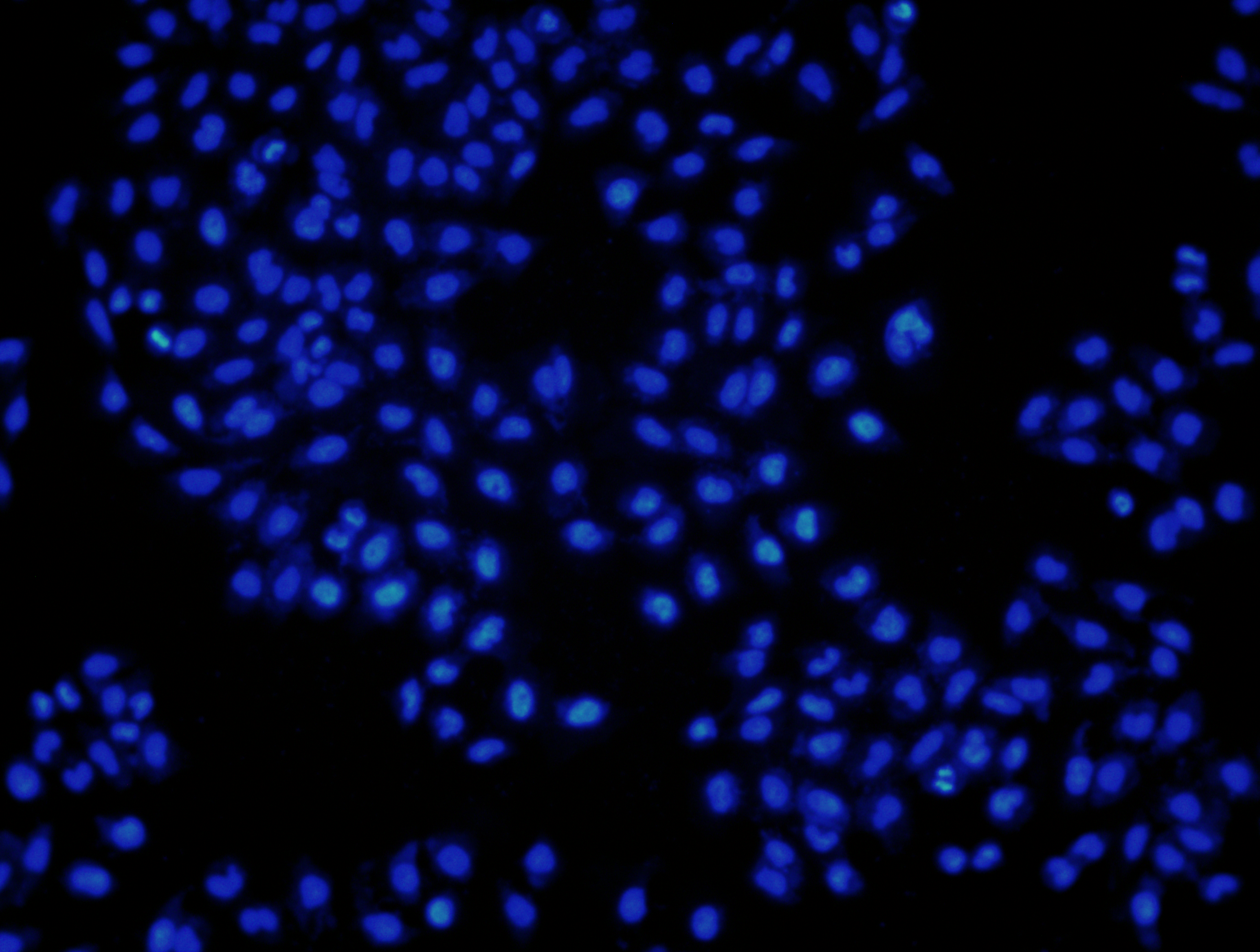

Supplement: Supplementary file 4 [file DataSheet_4.zip › Micrograph Figure S2 Li7 edu/Micrograph Figure S2-li7 fbxo9 hoechst.png]

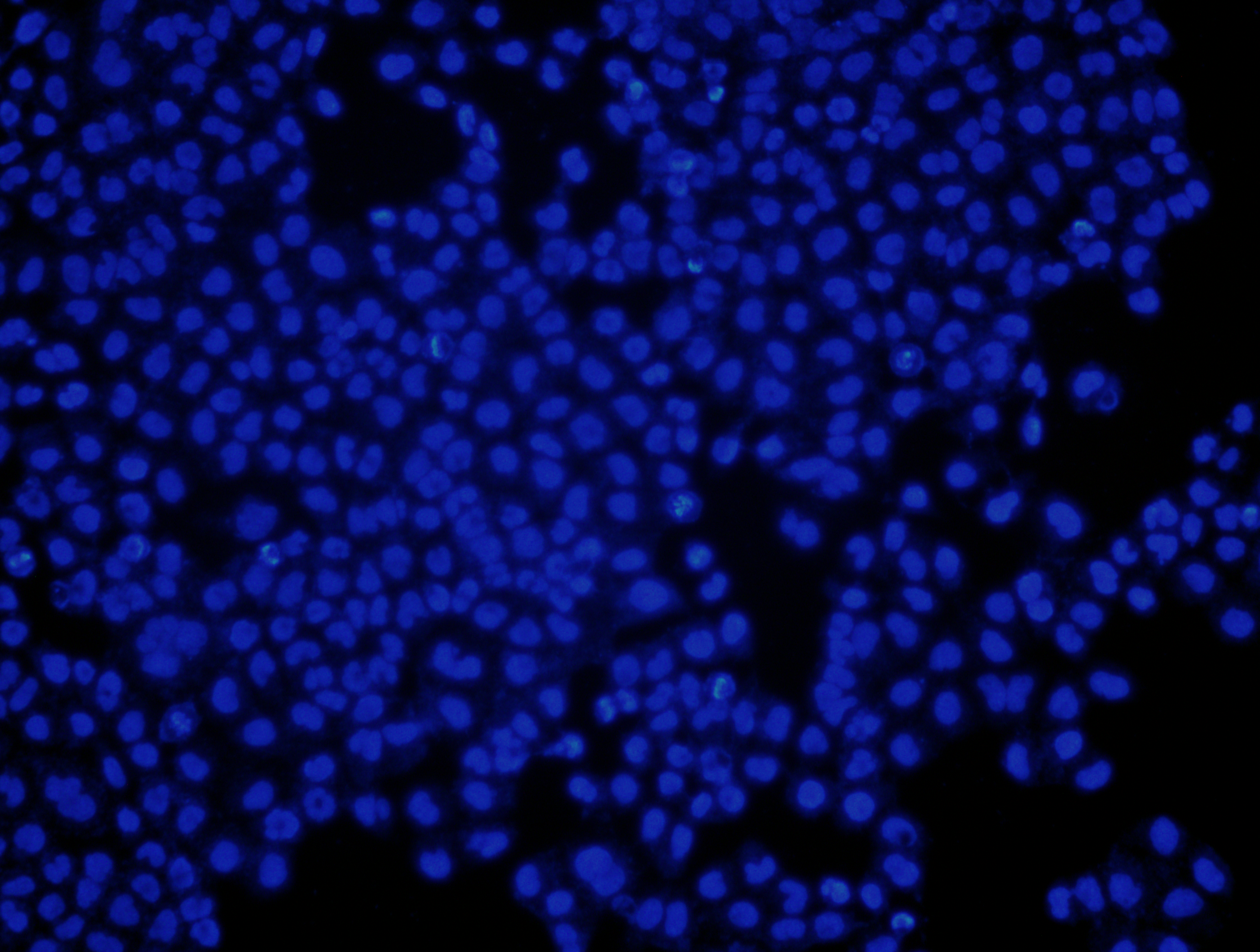

Supplement: Supplementary file 5 [file DataSheet_5.zip › Micrograph Figure S2 LM3 edu/Micrograph Figure S2-LM3 shFBXO9#1 HOECHST.png]

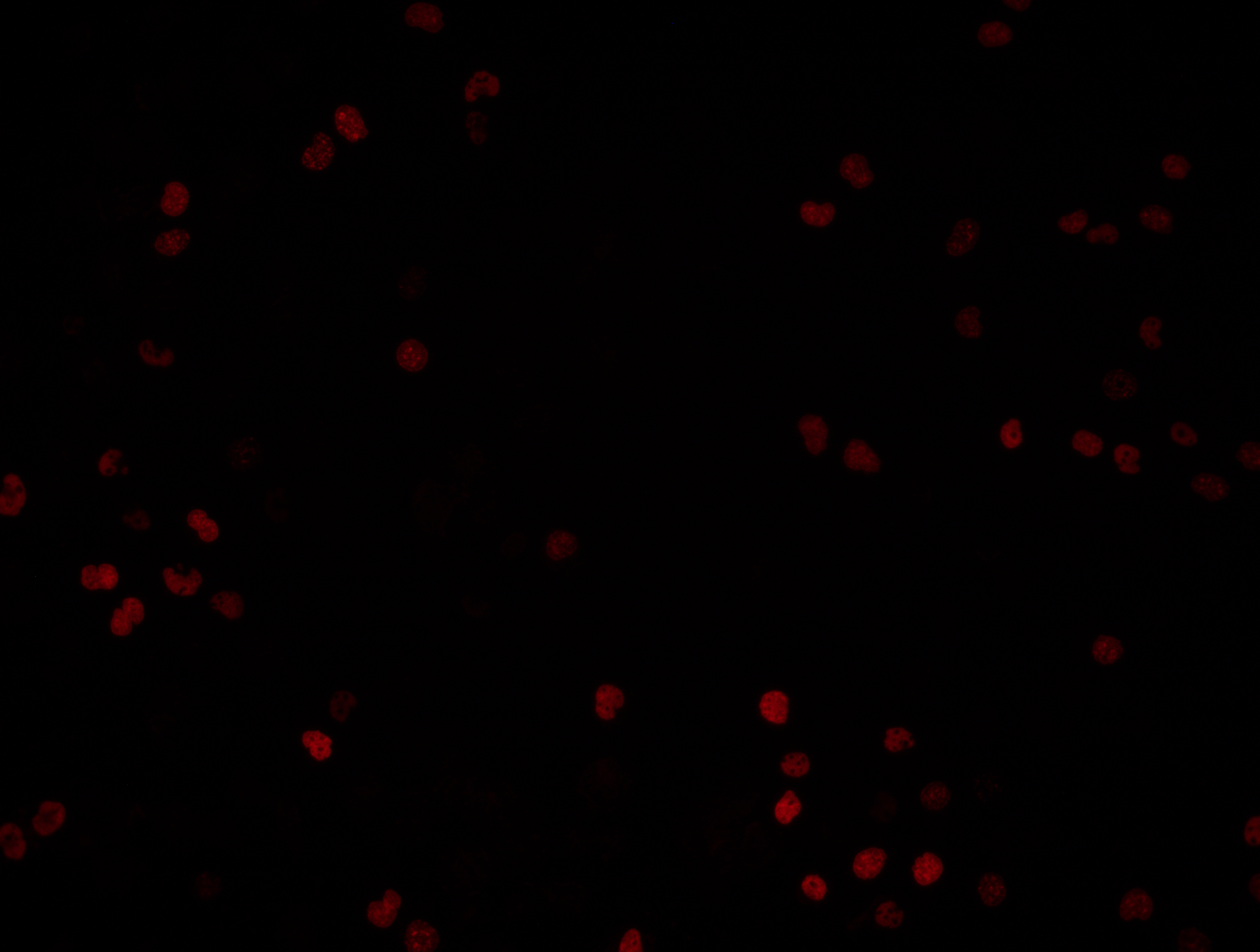

Supplement: Supplementary file 5 [file DataSheet_5.zip › Micrograph Figure S2 LM3 edu/Micrograph Figure S2-lm3 mock edu.png]

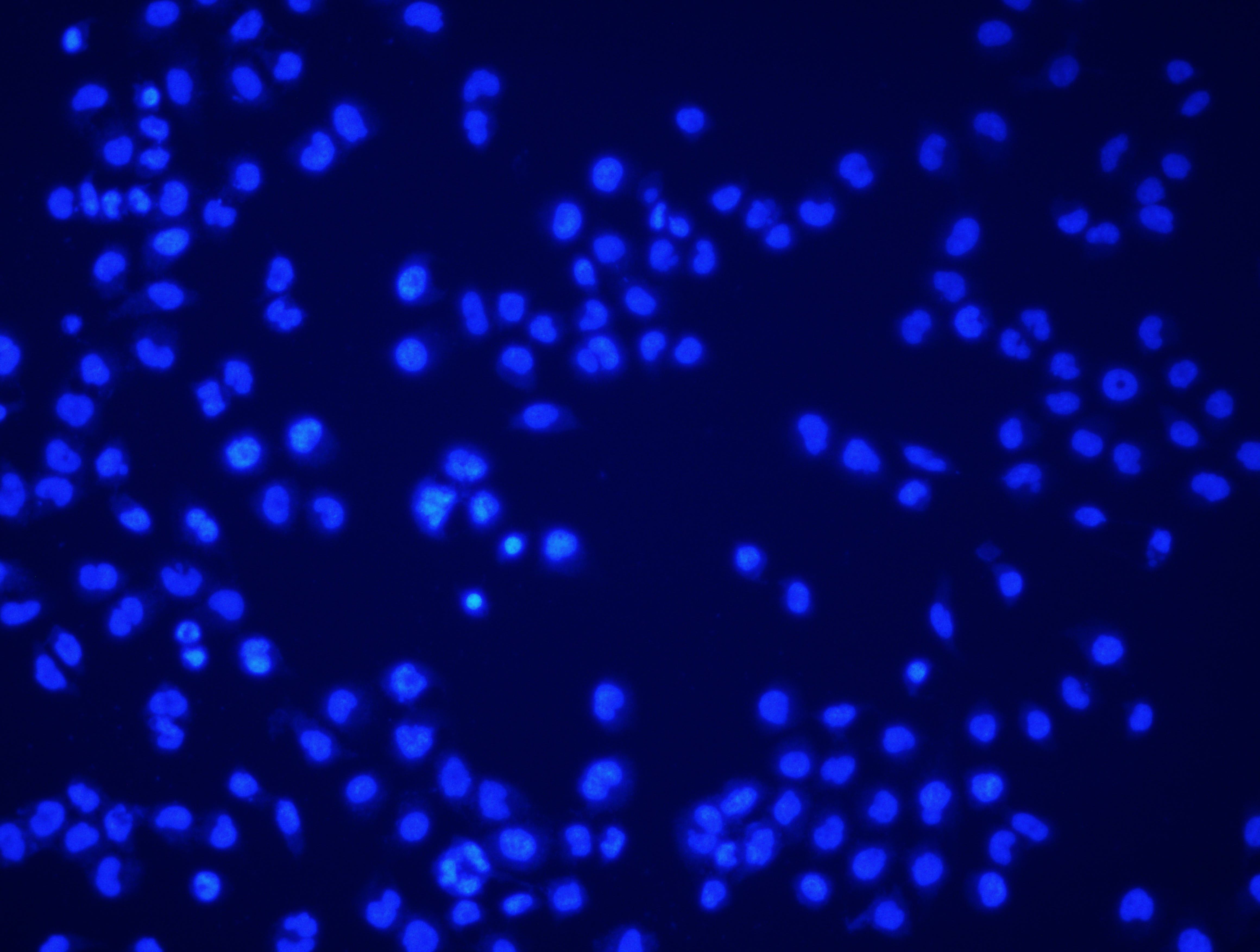

Supplement: Supplementary file 5 [file DataSheet_5.zip › Micrograph Figure S2 LM3 edu/Micrograph Figure S2-lm3 mock hoechst.png]

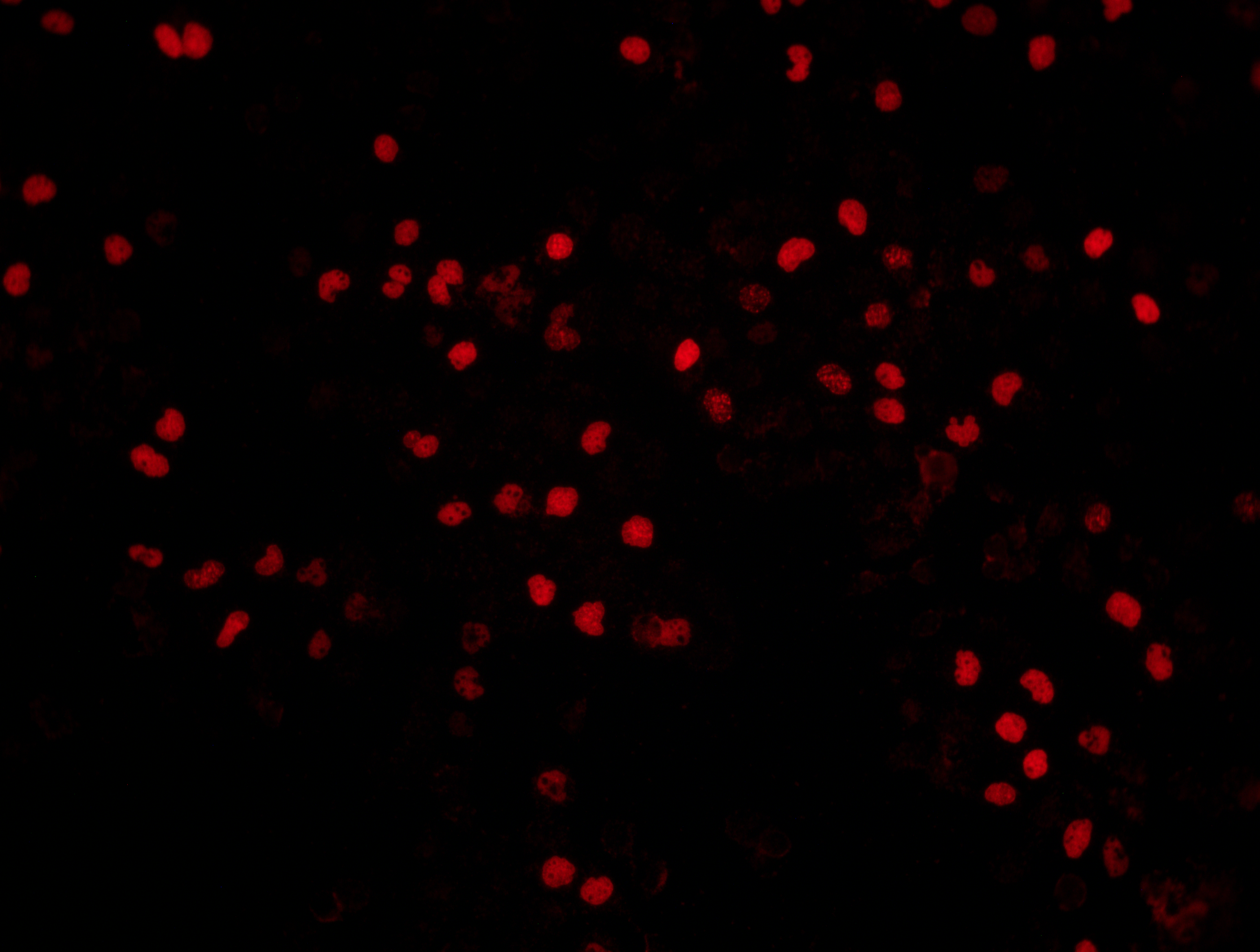

Supplement: Supplementary file 5 [file DataSheet_5.zip › Micrograph Figure S2 LM3 edu/Micrograph Figure S2-lm3 nc edu.png]

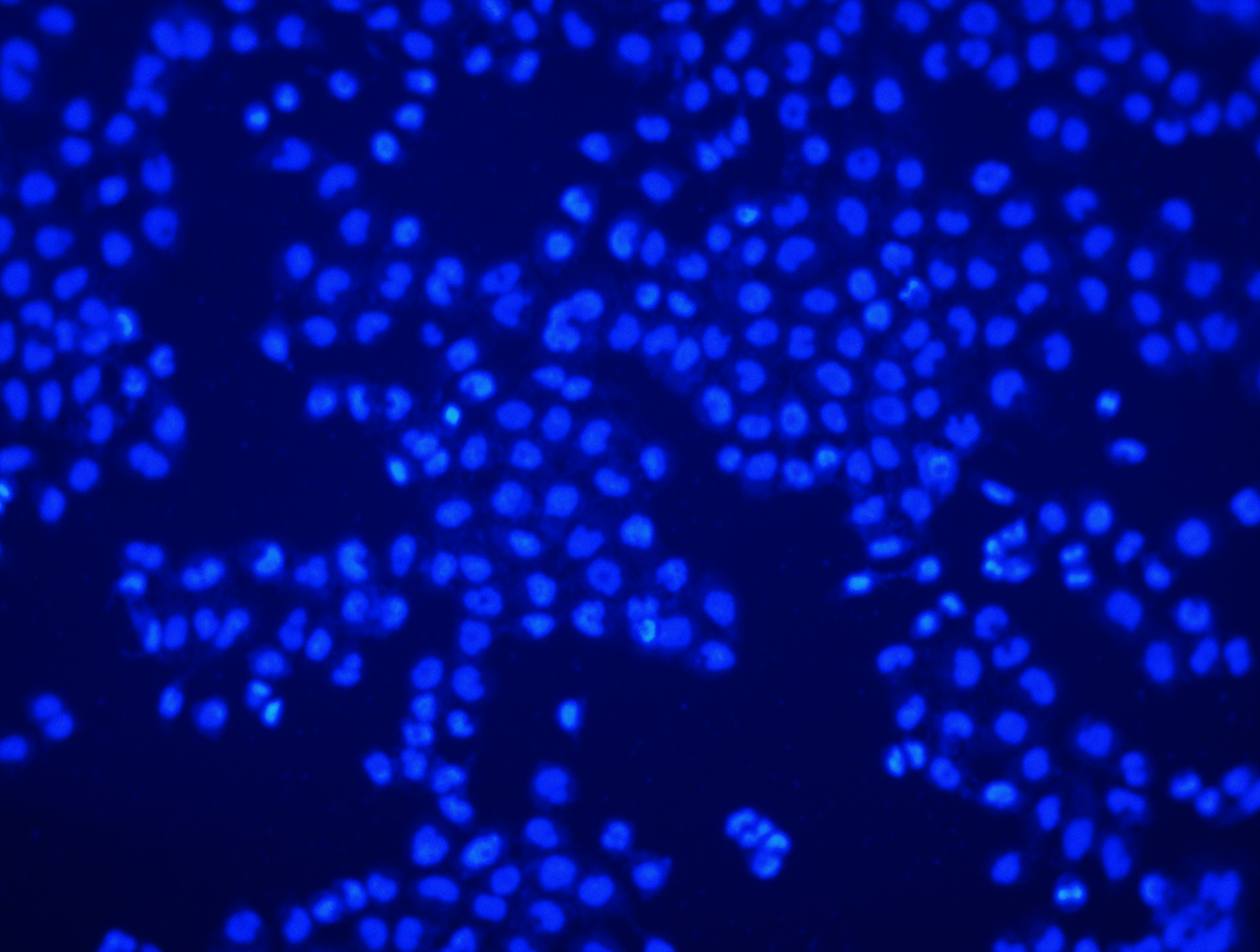

Supplement: Supplementary file 5 [file DataSheet_5.zip › Micrograph Figure S2 LM3 edu/Micrograph Figure S2-lm3 nc hoechst.png]

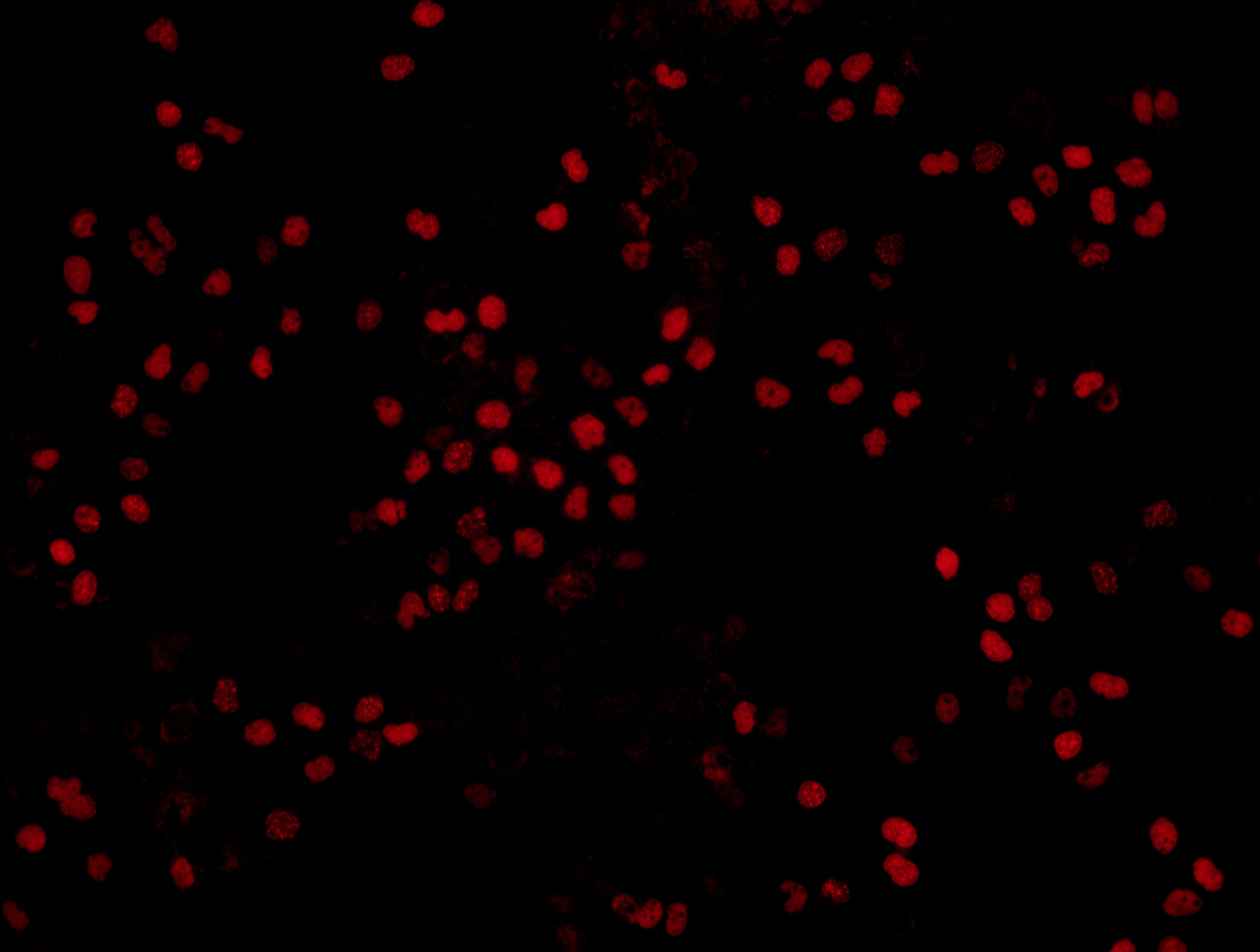

Supplement: Supplementary file 5 [file DataSheet_5.zip › Micrograph Figure S2 LM3 edu/Micrograph Figure S2-lm3 shFBXO9#1 EDU.png]

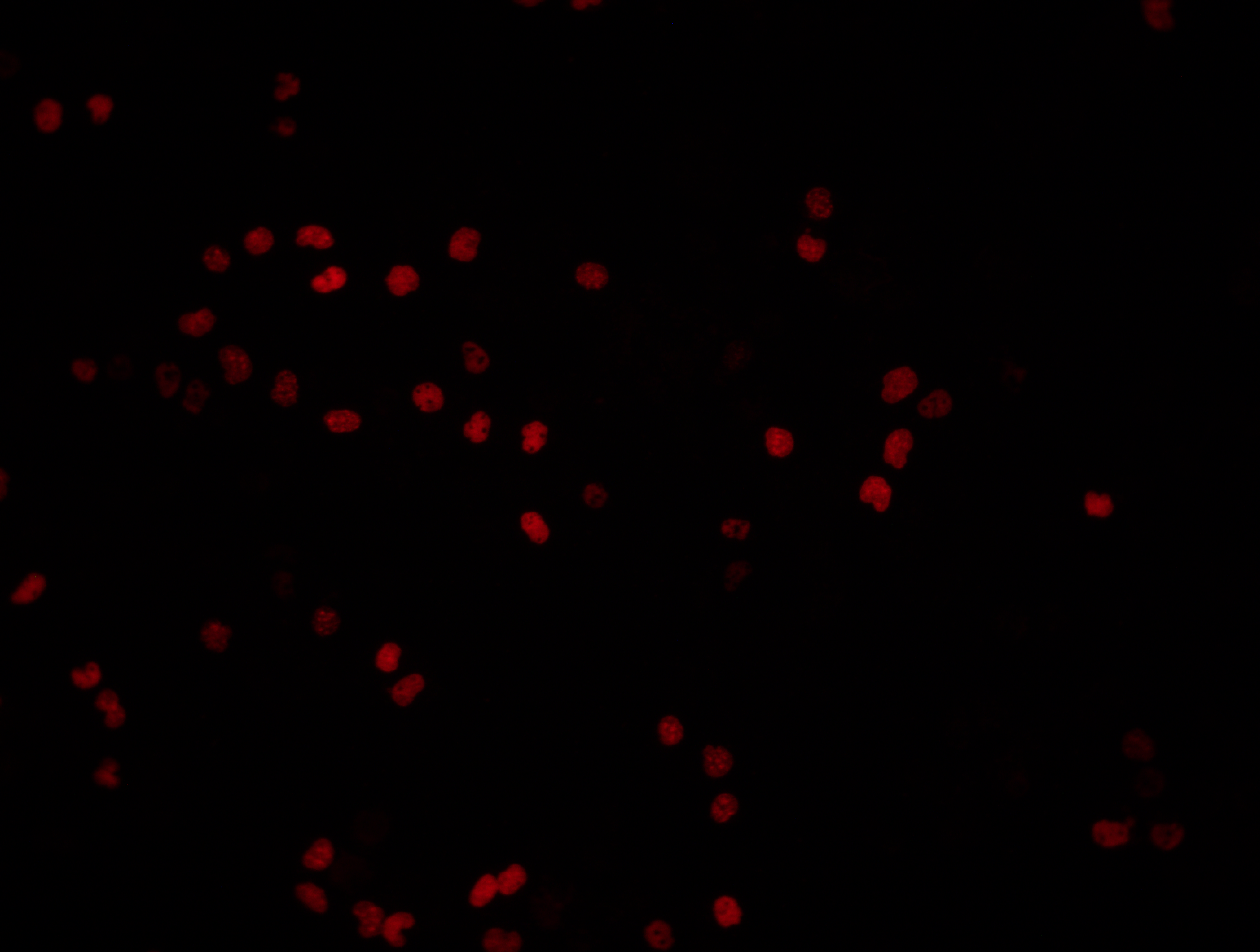

Supplement: Supplementary file 5 [file DataSheet_5.zip › Micrograph Figure S2 LM3 edu/Micrograph Figure S2-lm3 shfbxo9#2 edu.png]

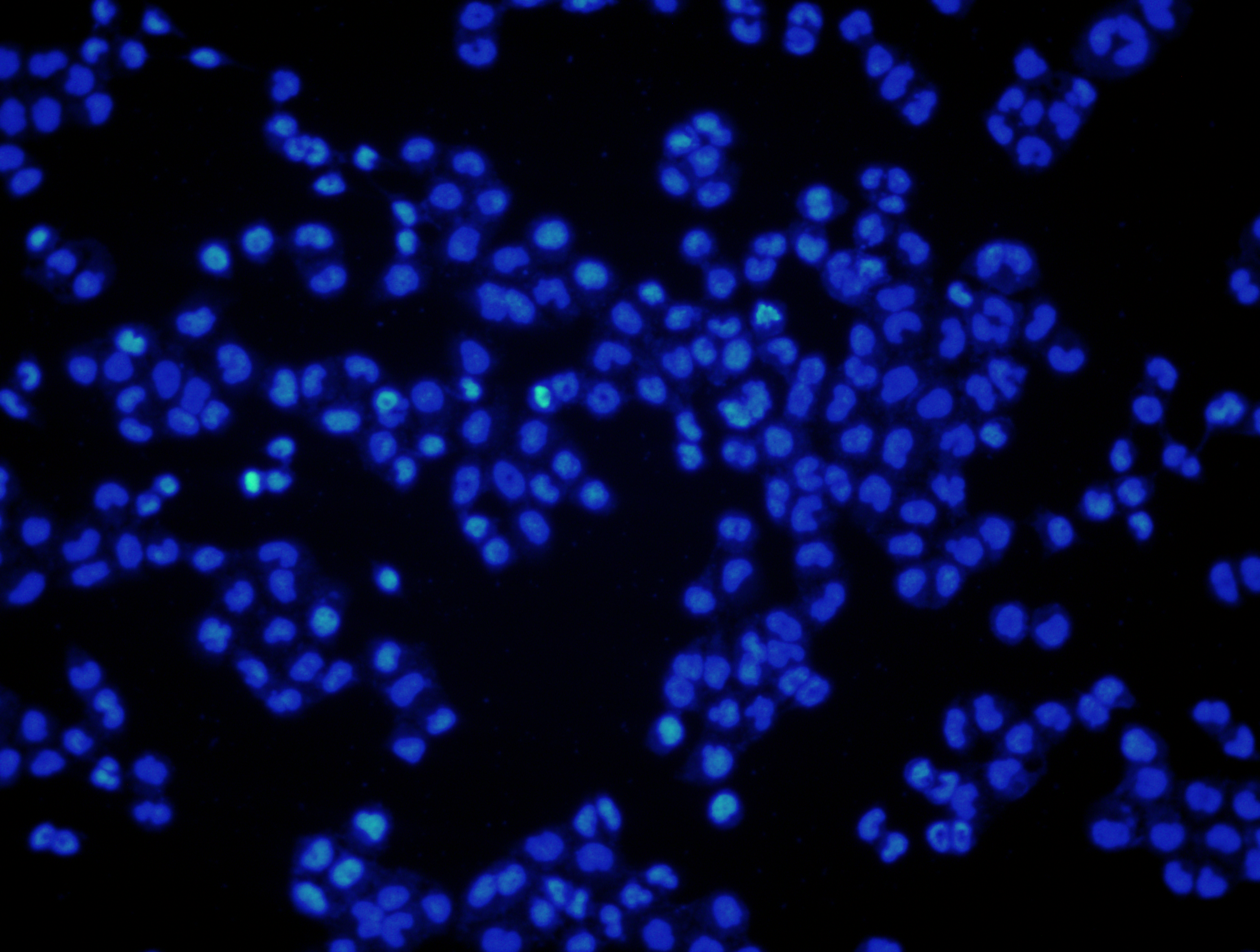

Supplement: Supplementary file 5 [file DataSheet_5.zip › Micrograph Figure S2 LM3 edu/Micrograph Figure S2-lm3 shfbxo9#2 hoechst.png]

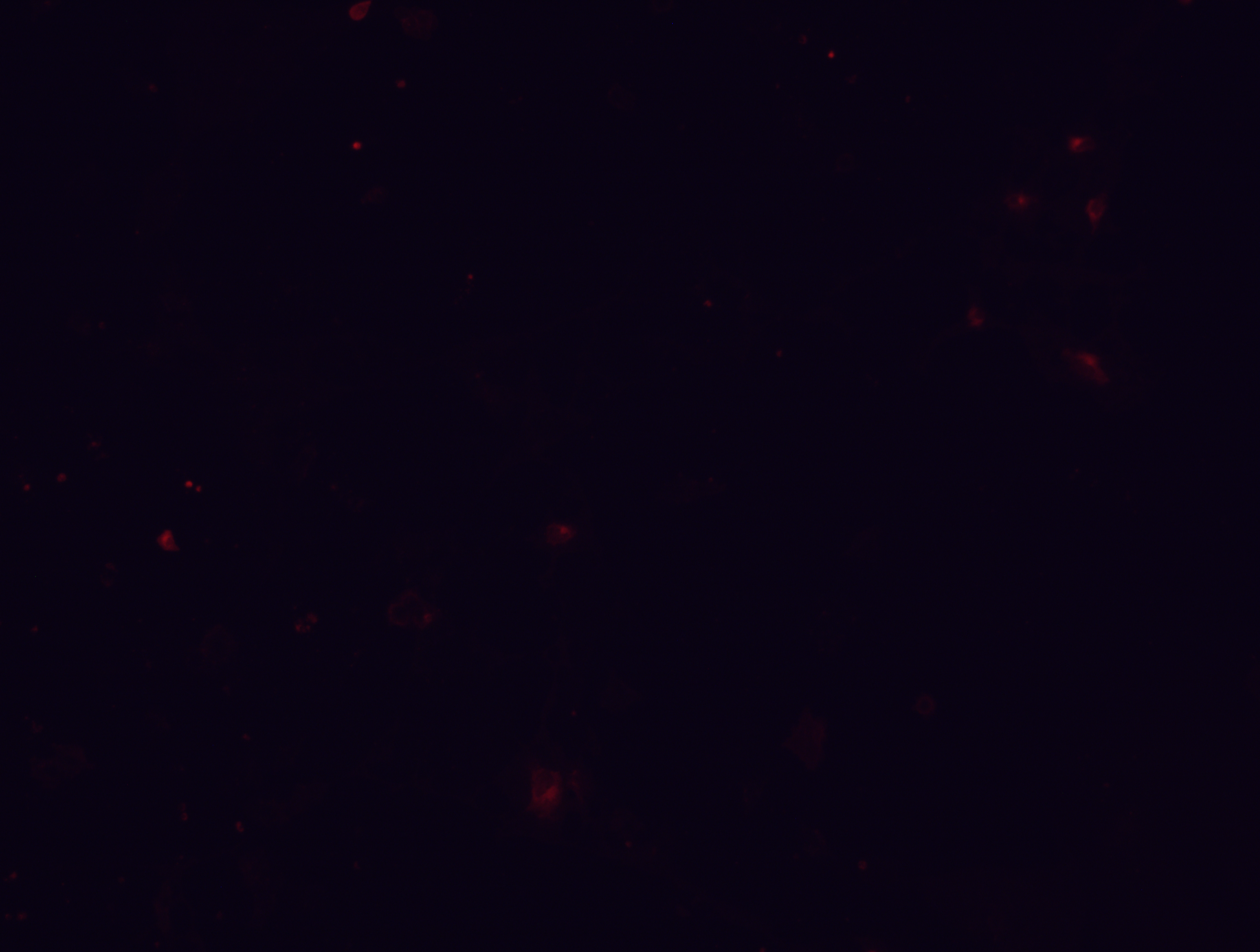

Supplement: Supplementary file 6 [file DataSheet_6.zip › Micrograph Figure S2-LI7 Annexin V/Micrograph Figure S2-LI7 FBXO9 Annexin V.png]

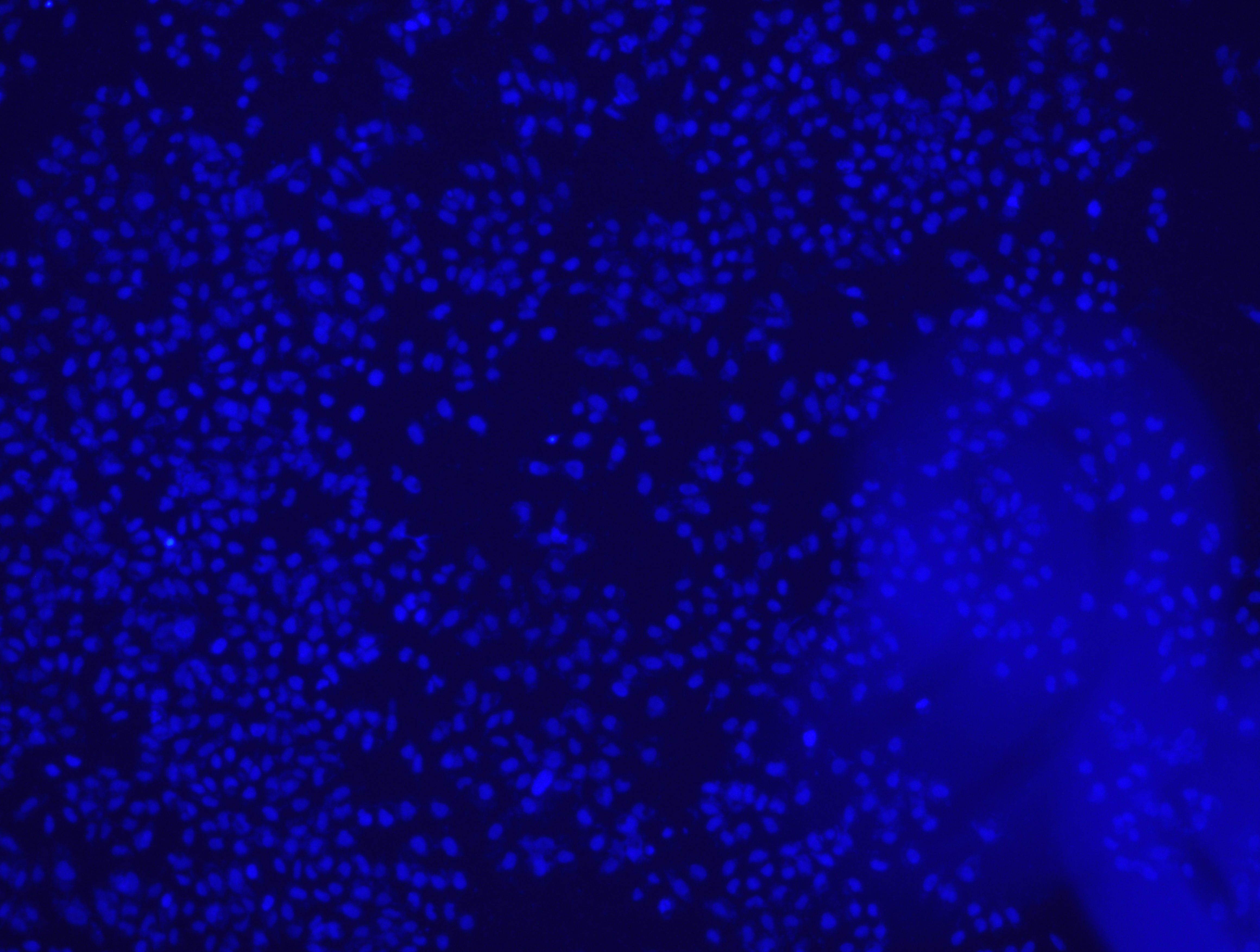

Supplement: Supplementary file 6 [file DataSheet_6.zip › Micrograph Figure S2-LI7 Annexin V/Micrograph Figure S2-LI7 FBXO9 HOECHST.png]

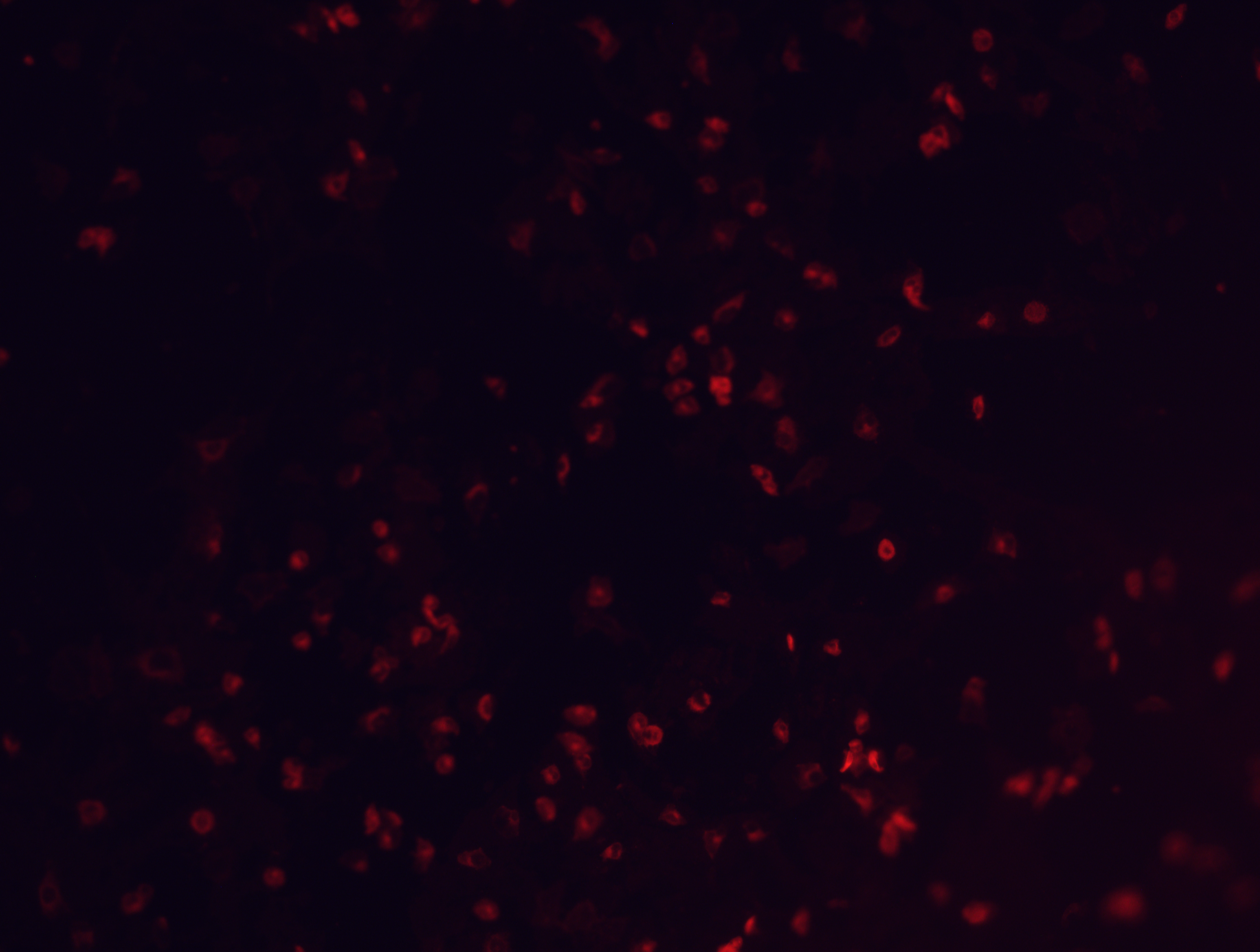

Supplement: Supplementary file 6 [file DataSheet_6.zip › Micrograph Figure S2-LI7 Annexin V/Micrograph Figure S2-LI7 VECTOR Annexin V.png]

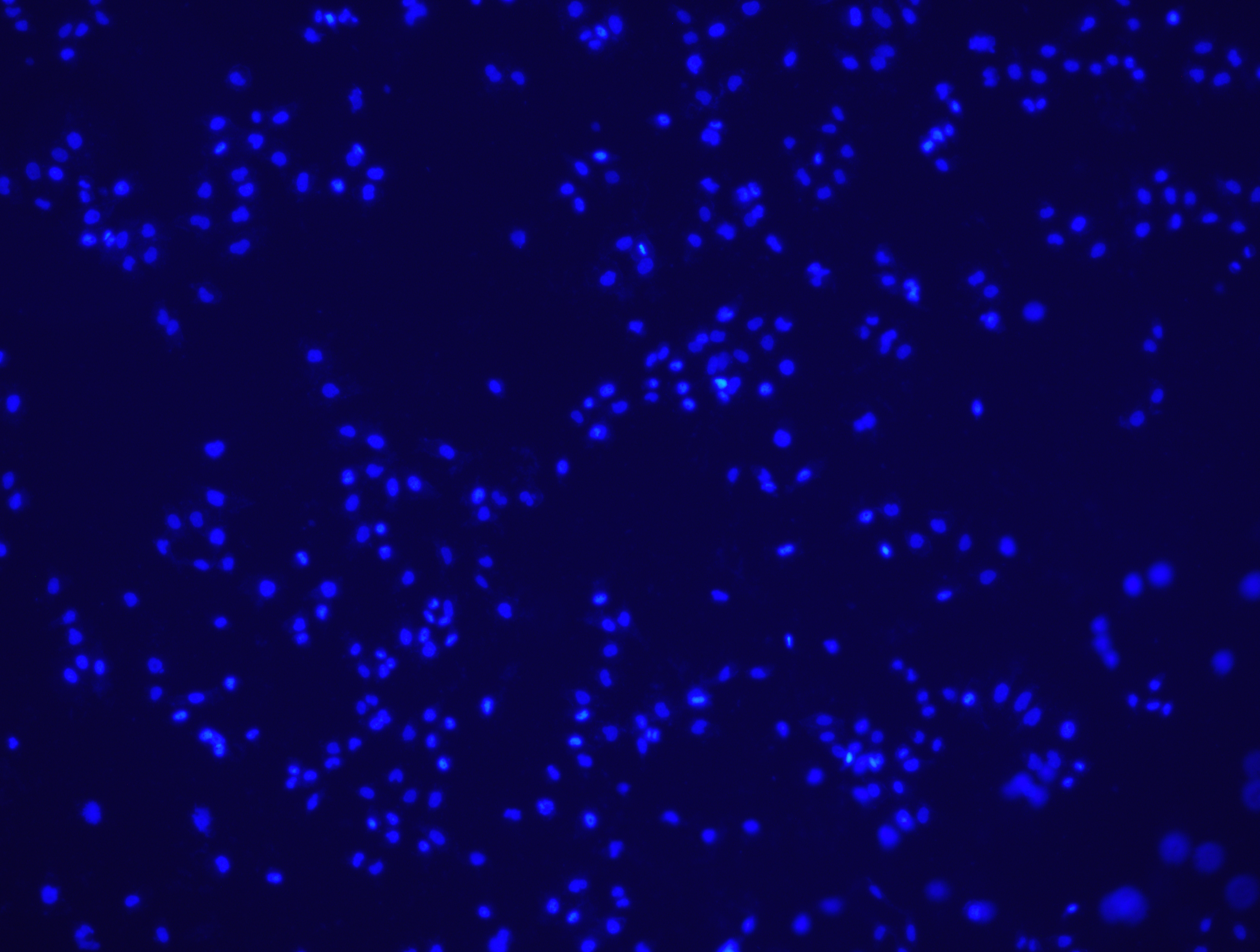

Supplement: Supplementary file 6 [file DataSheet_6.zip › Micrograph Figure S2-LI7 Annexin V/Micrograph Figure S2-LI7 VECTOR HOECHST.png]

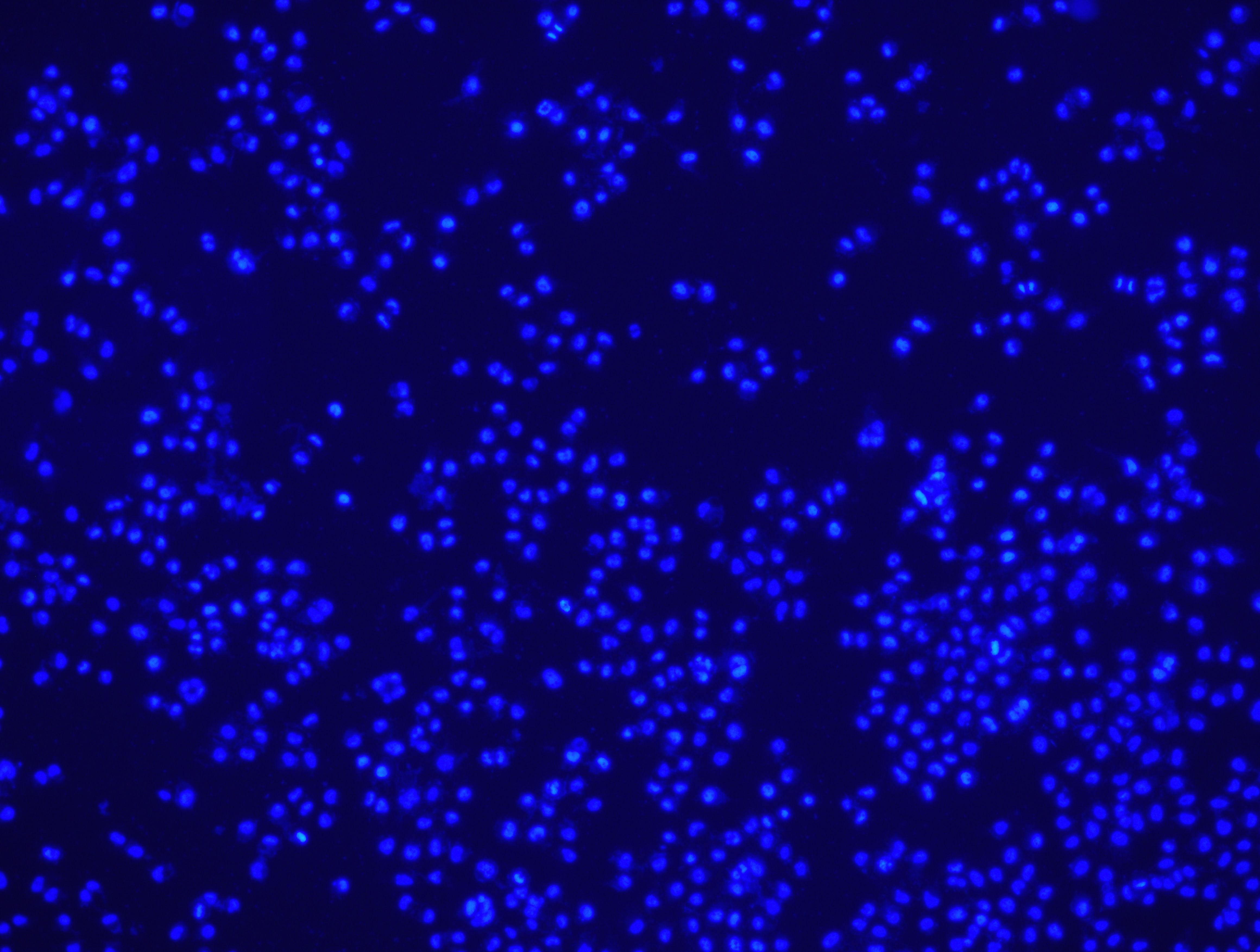

Supplement: Supplementary file 7 [file DataSheet_7.zip › Micrograph Figure S2-lm3 Annexin V/Micrograph Figure S2-LM3 MOCK HOECHST.png]

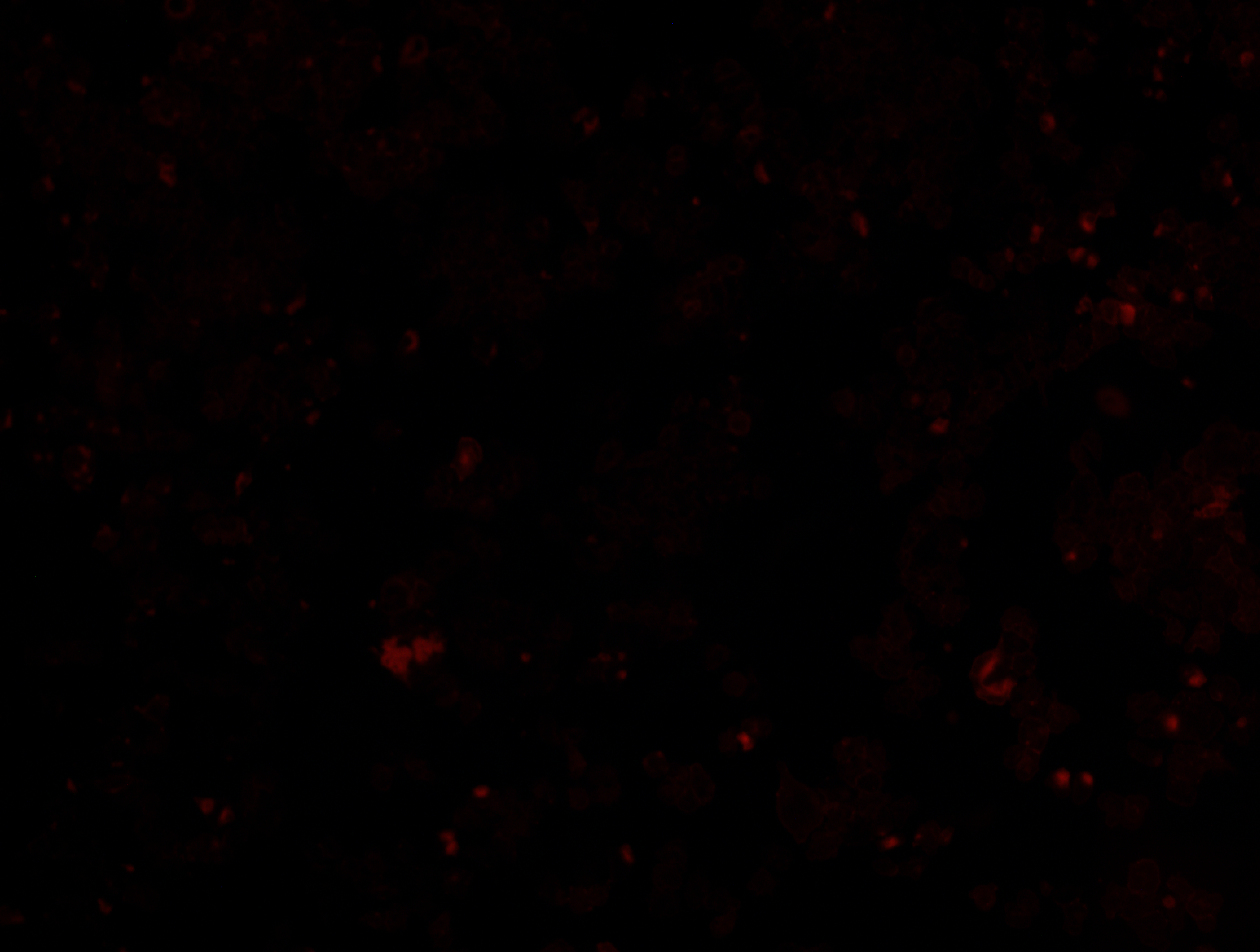

Supplement: Supplementary file 7 [file DataSheet_7.zip › Micrograph Figure S2-lm3 Annexin V/Micrograph Figure S2-LM3 SHNC Annexin V.png]

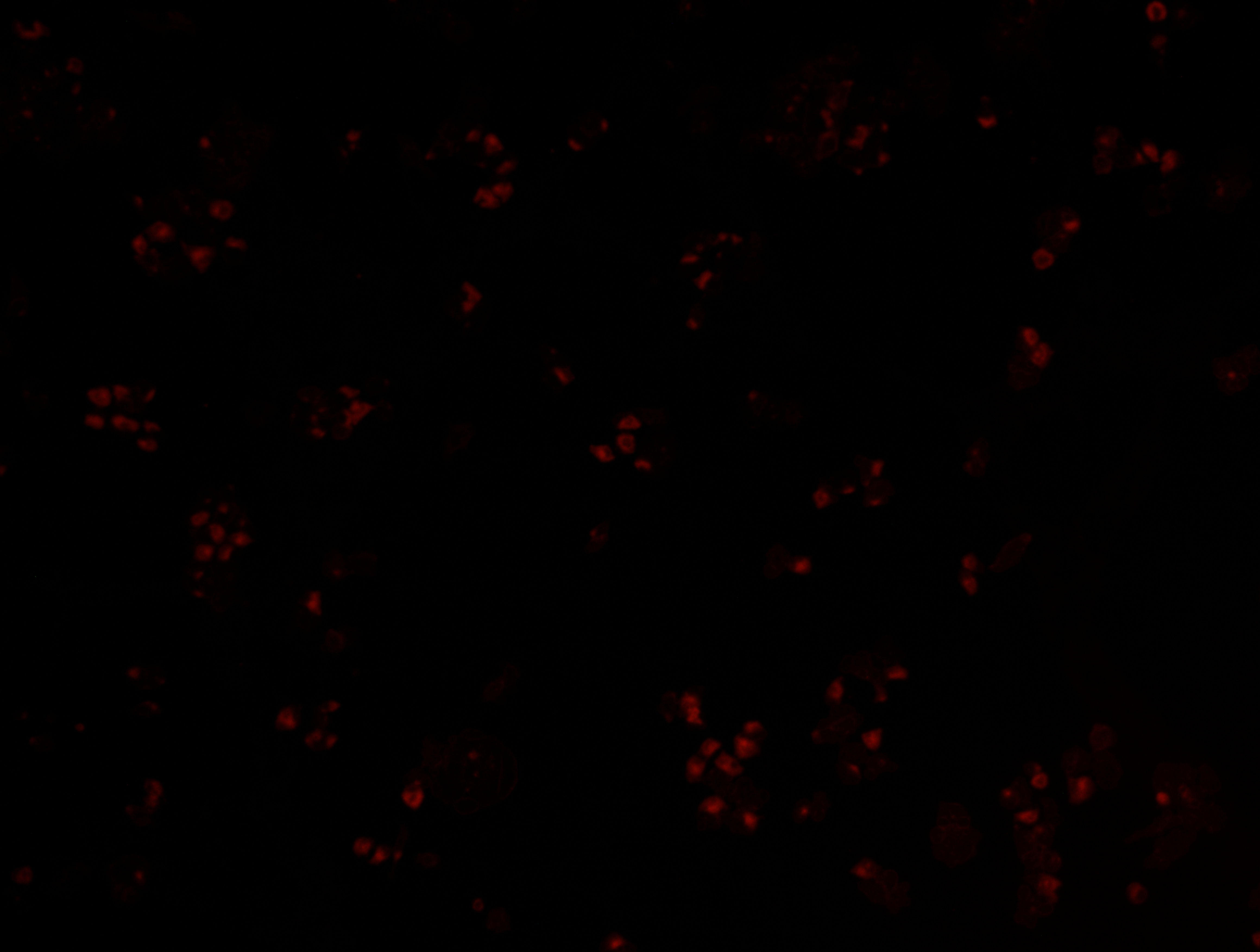

Supplement: Supplementary file 7 [file DataSheet_7.zip › Micrograph Figure S2-lm3 Annexin V/Micrograph Figure S2-LM3 shFBXO9#1 Annexin V.png]

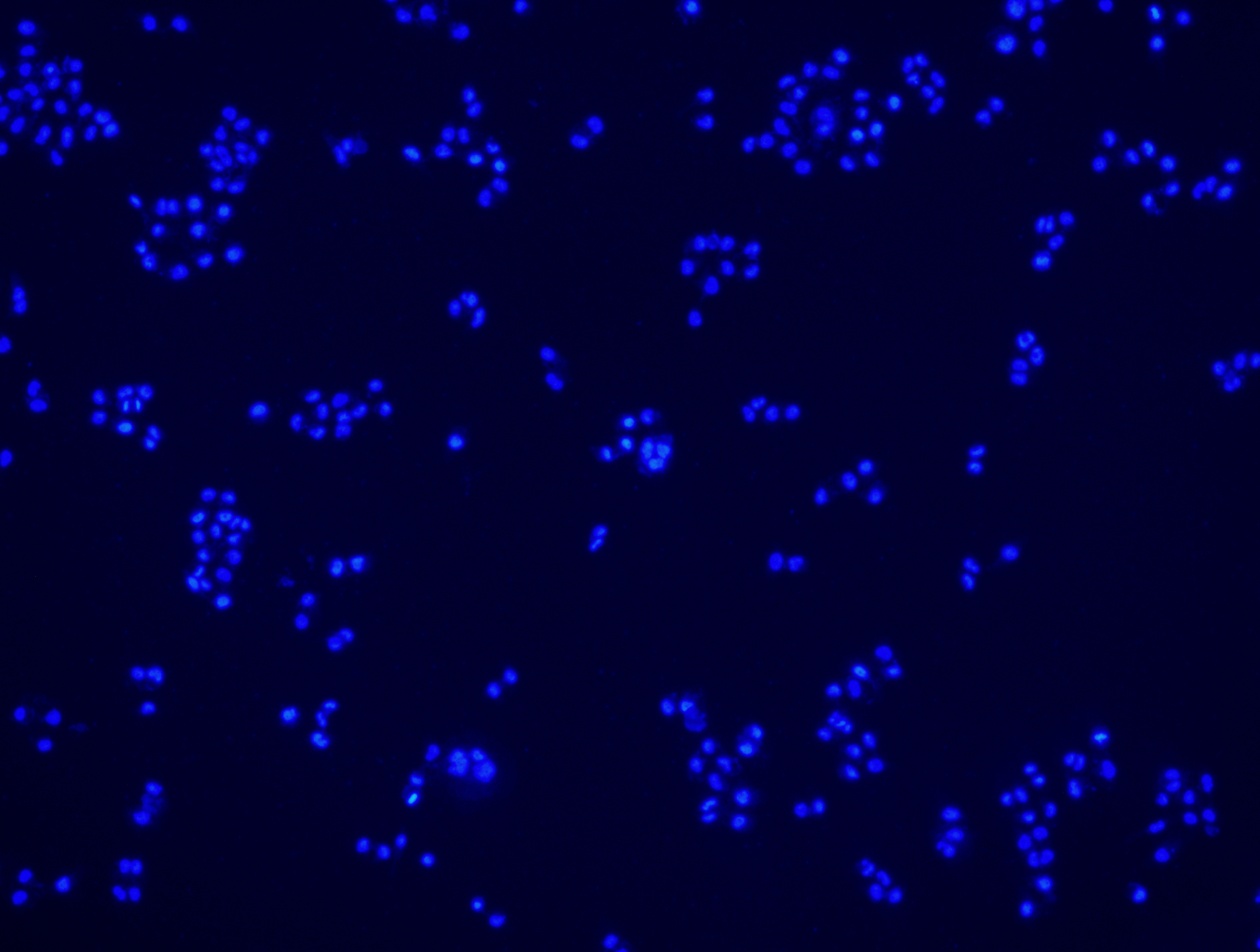

Supplement: Supplementary file 7 [file DataSheet_7.zip › Micrograph Figure S2-lm3 Annexin V/Micrograph Figure S2-LM3 shFBXO9#1 Hoechst.png]

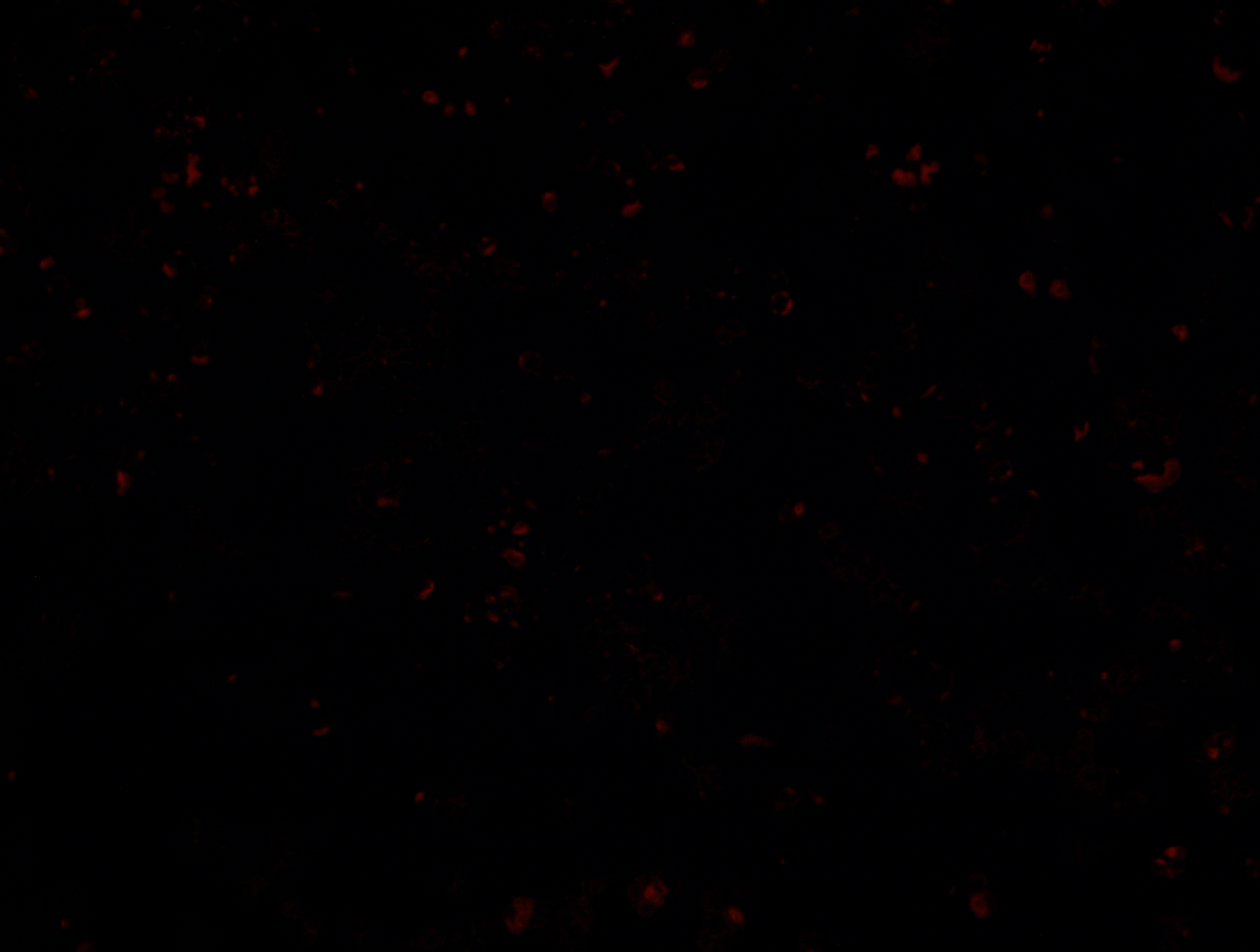

Supplement: Supplementary file 7 [file DataSheet_7.zip › Micrograph Figure S2-lm3 Annexin V/Micrograph Figure S2-LM3 shFBXO9#2 Annexin V.png]

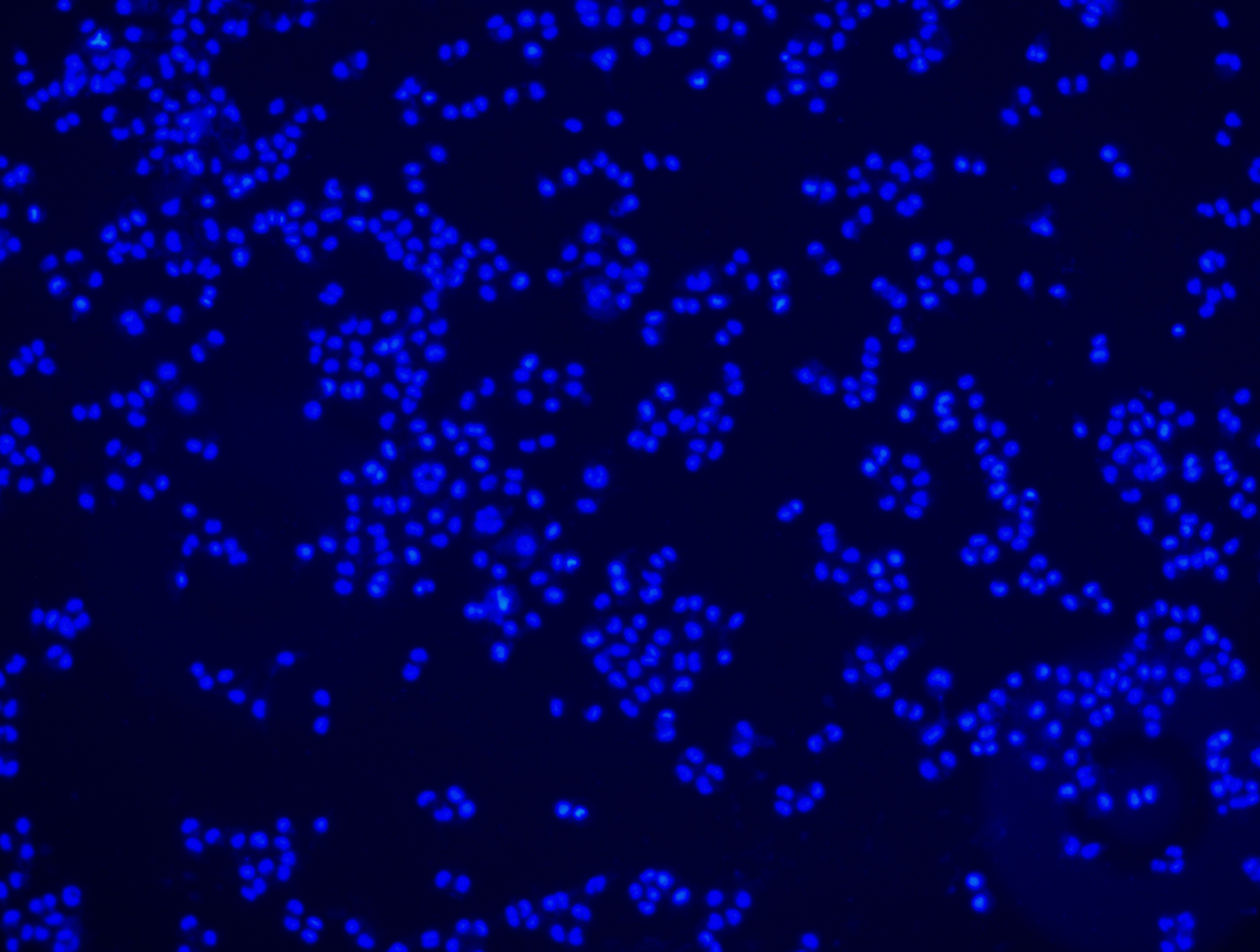

Supplement: Supplementary file 7 [file DataSheet_7.zip › Micrograph Figure S2-lm3 Annexin V/Micrograph Figure S2-LM3 shFBXO9#2 Hoechst.png]

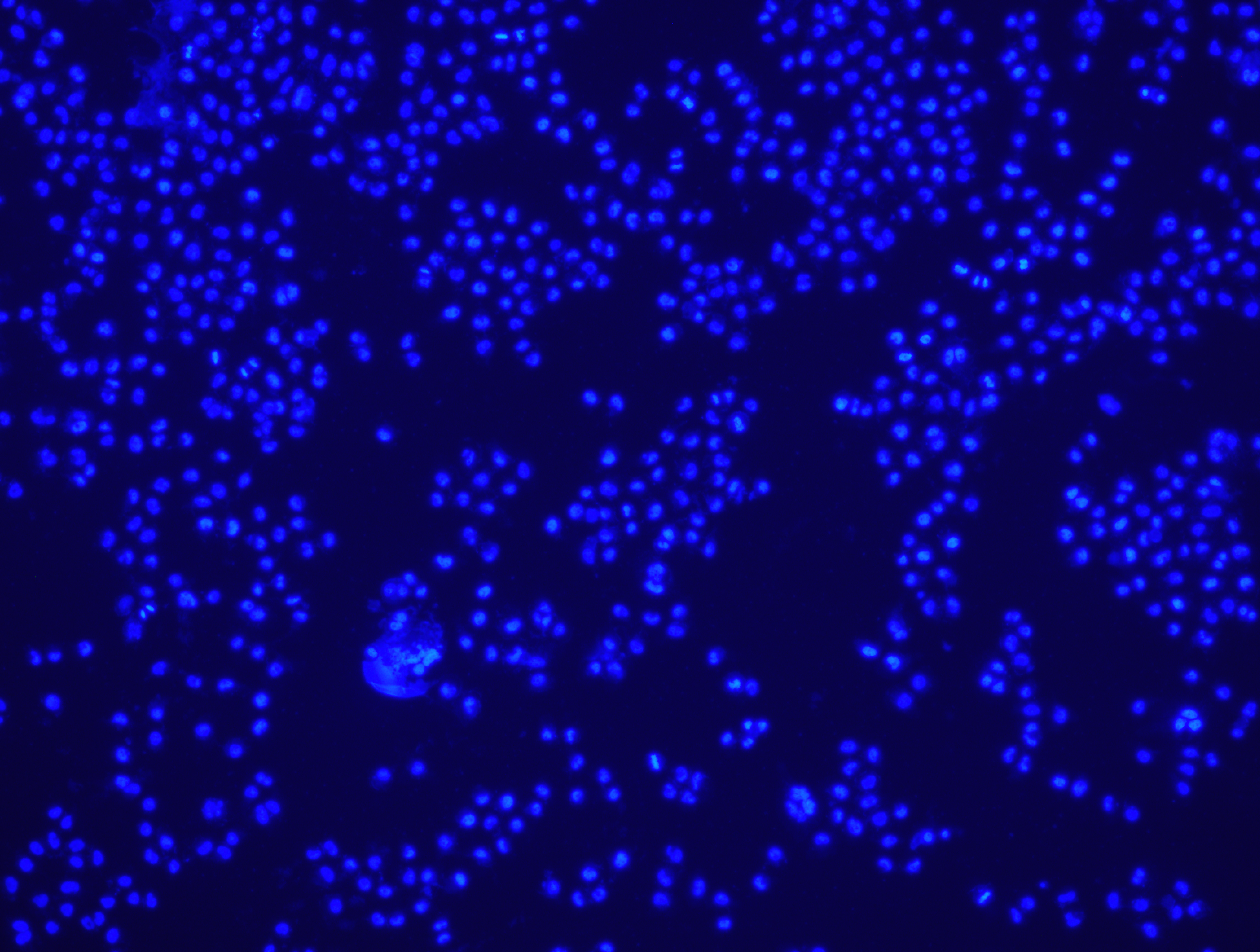

Supplement: Supplementary file 7 [file DataSheet_7.zip › Micrograph Figure S2-lm3 Annexin V/Micrograph Figure S2-LM3 shnc hoechst.png]

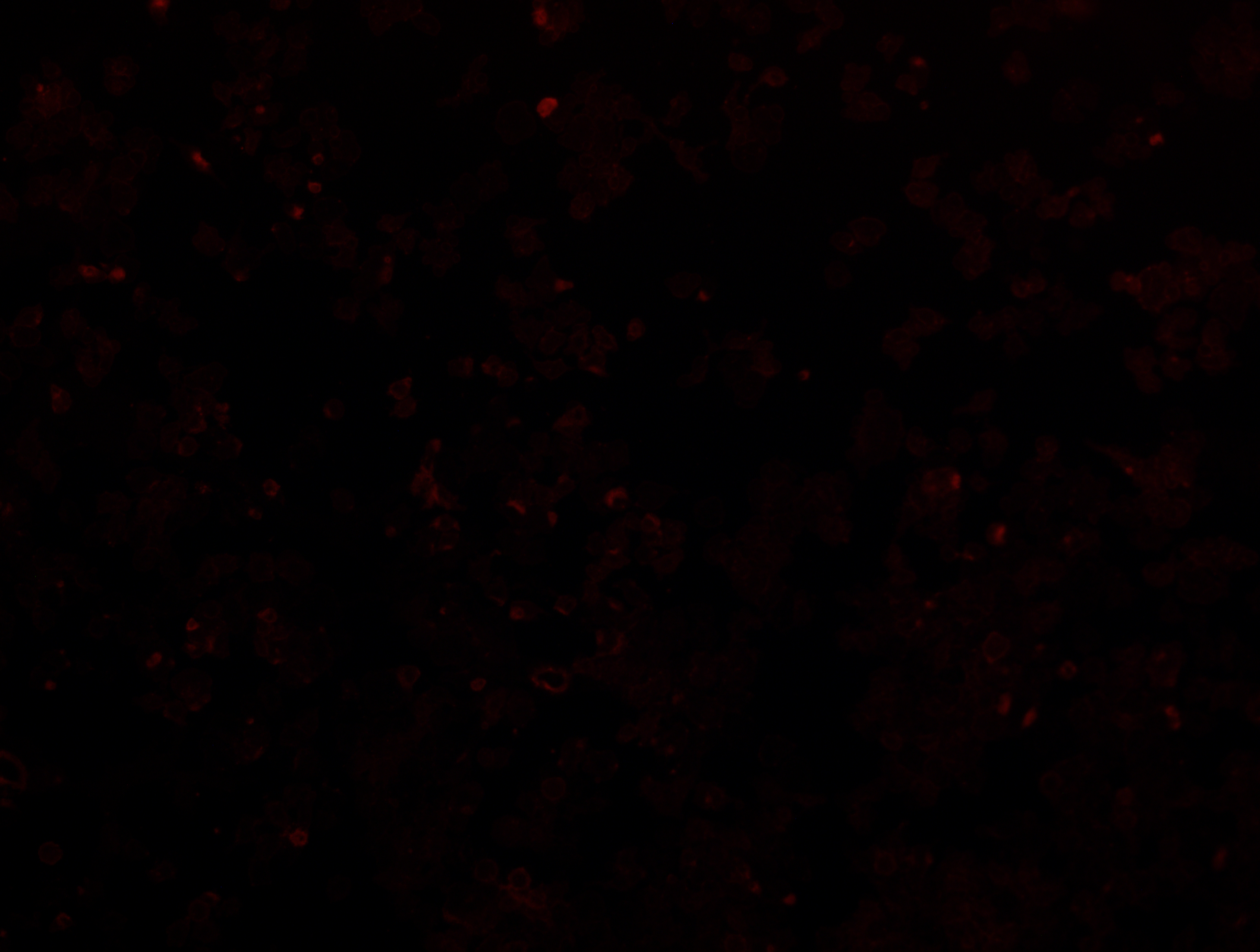

Supplement: Supplementary file 7 [file DataSheet_7.zip › Micrograph Figure S2-lm3 Annexin V/Micrograph Figure S2-lm3 mock Annexin V.png]

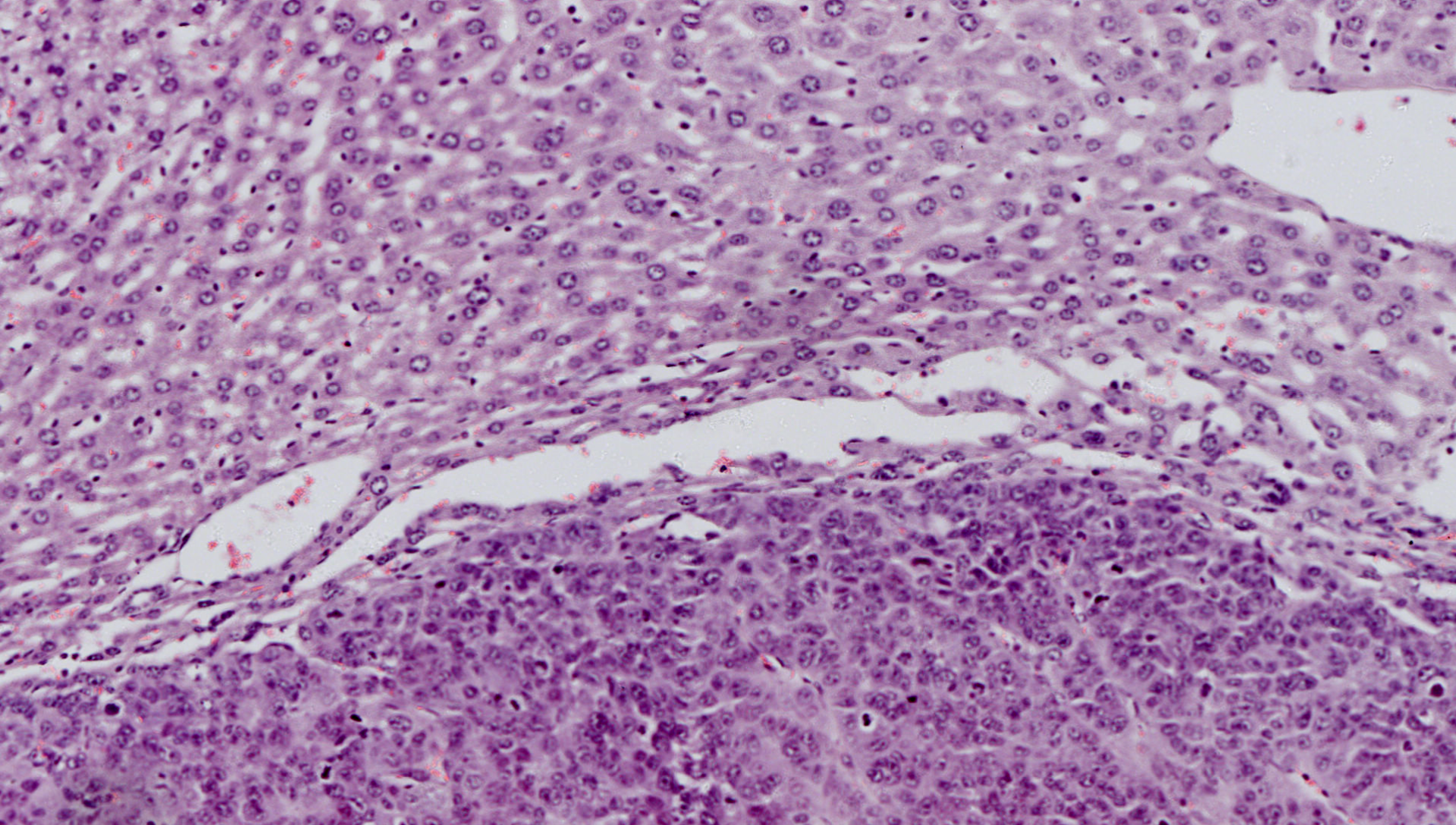

Supplement: Supplementary file 8 [file DataSheet_8.zip › Micrograph Figure 2H-Liver/Liver FbxO9 20x.tif]

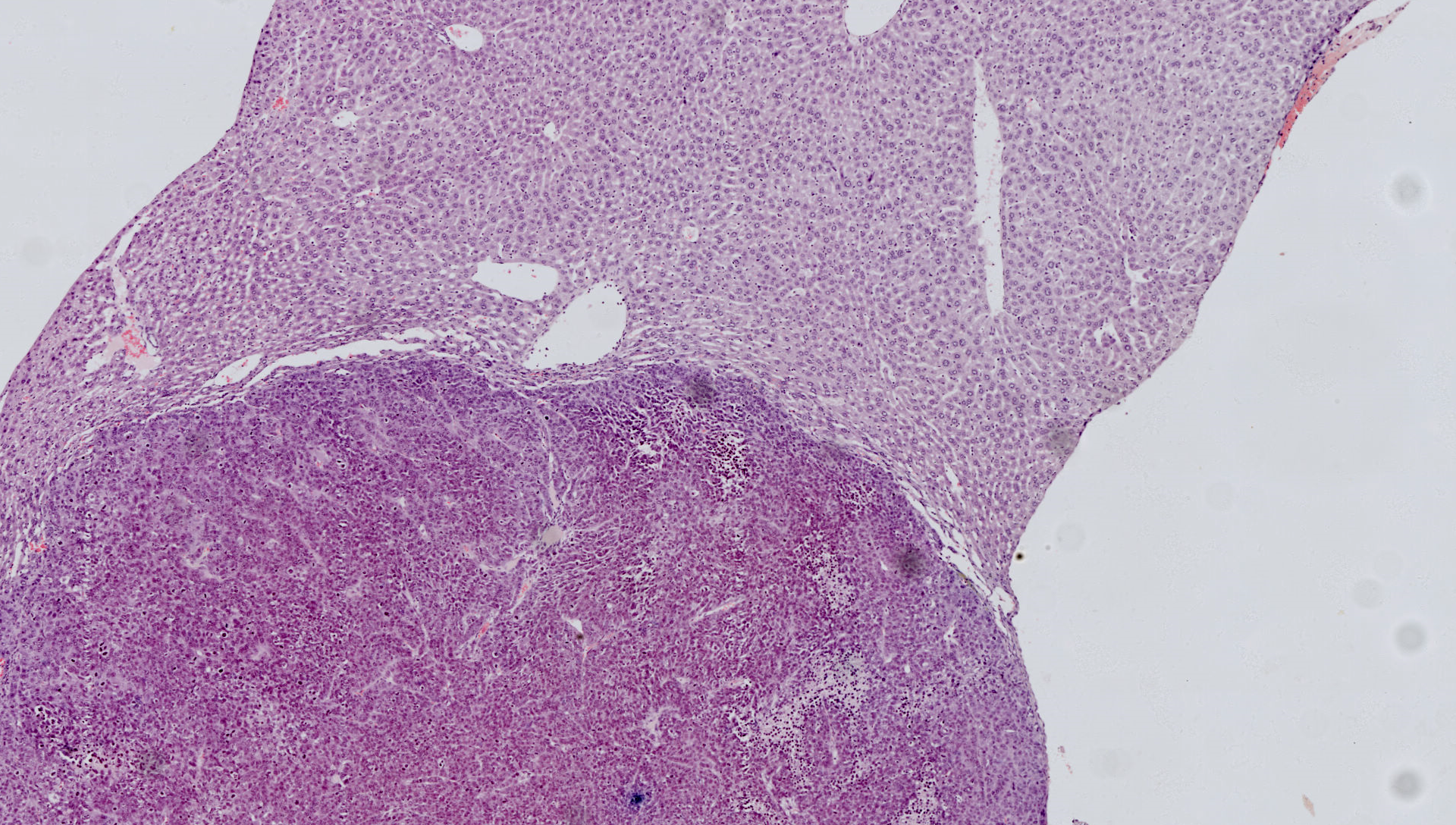

Supplement: Supplementary file 8 [file DataSheet_8.zip › Micrograph Figure 2H-Liver/Liver FbxO9 5x.tif]

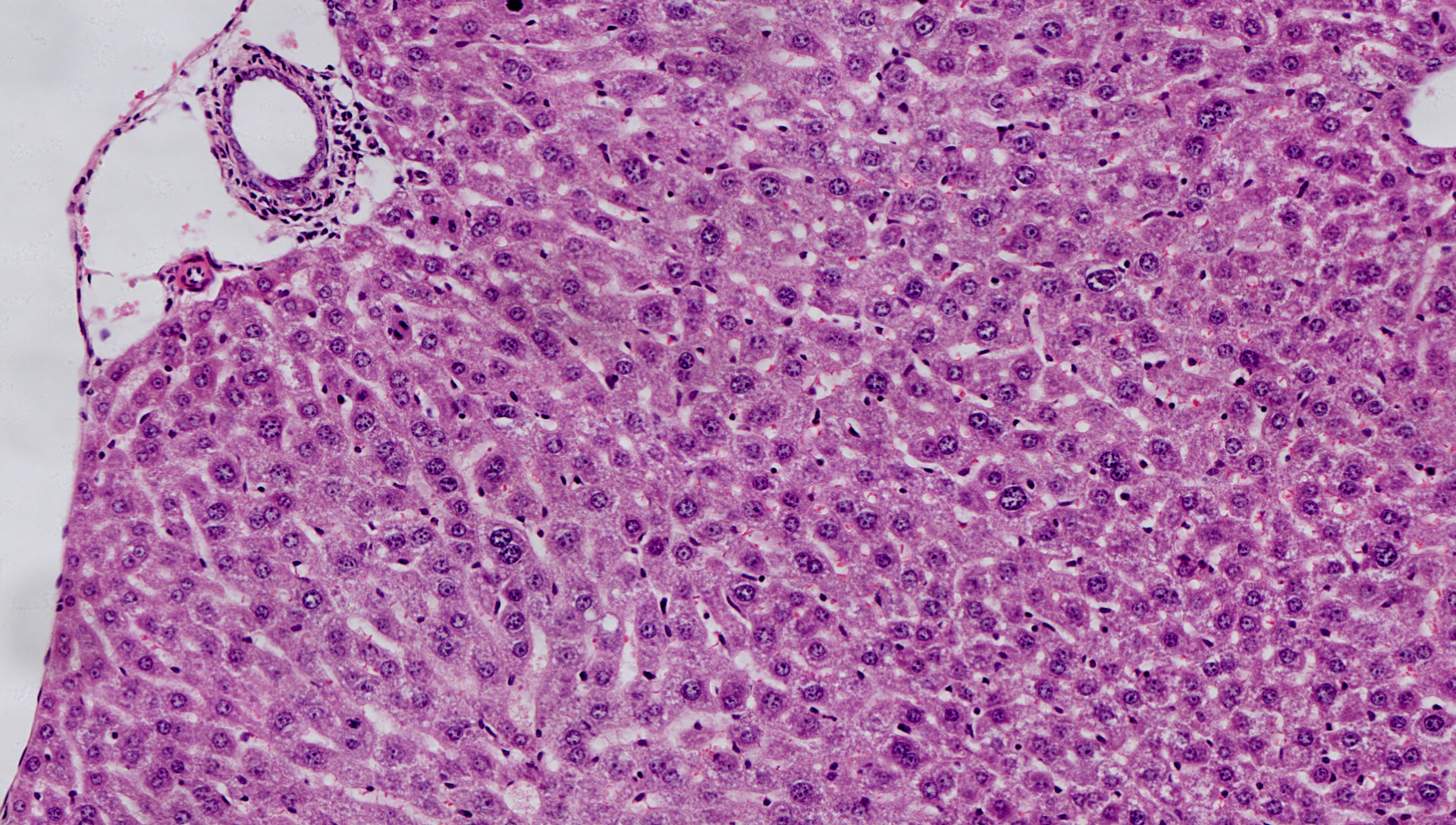

Supplement: Supplementary file 8 [file DataSheet_8.zip › Micrograph Figure 2H-Liver/Liver Vector 20x.tif]

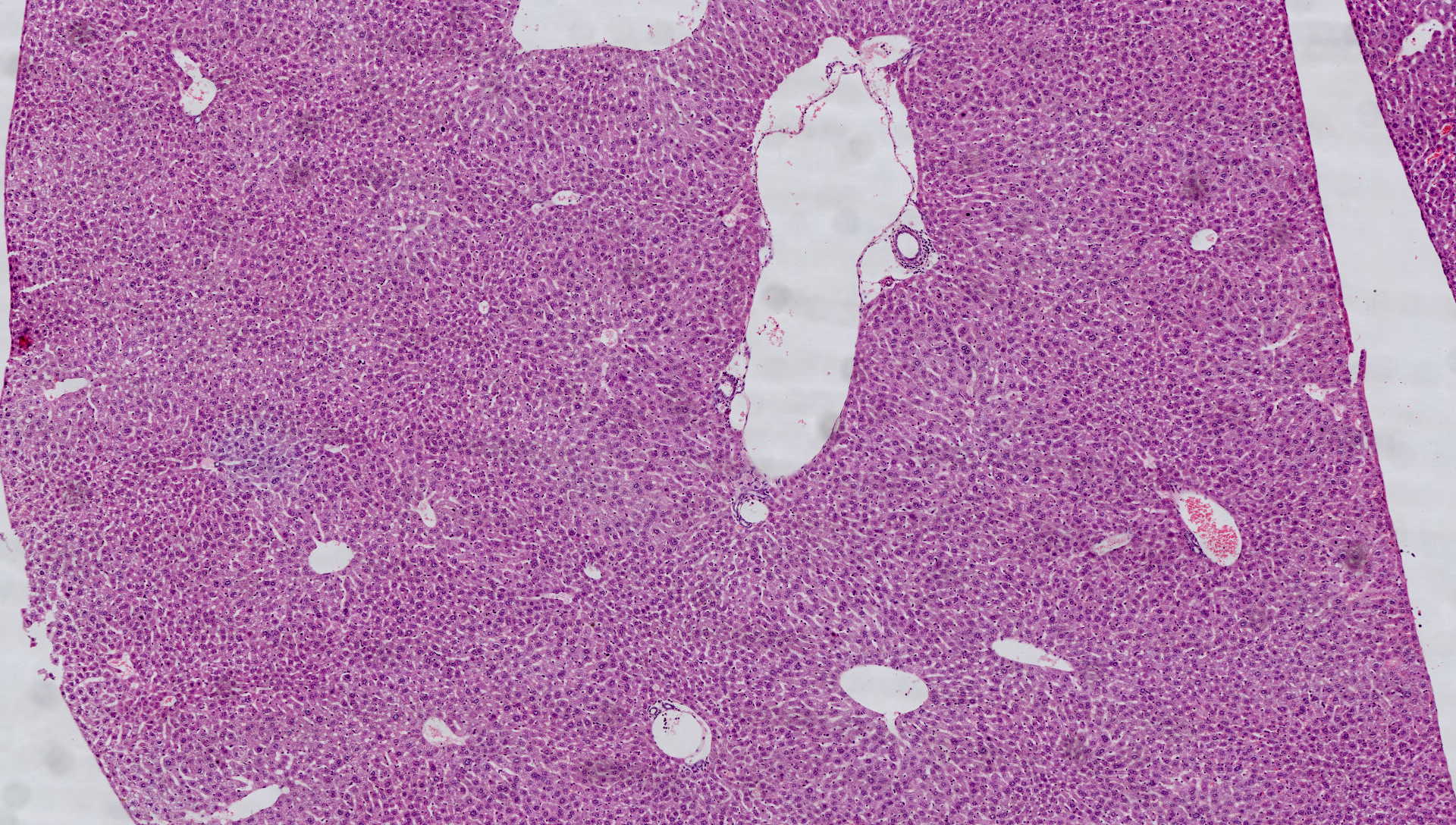

Supplement: Supplementary file 8 [file DataSheet_8.zip › Micrograph Figure 2H-Liver/Liver Vector 5x.tif]

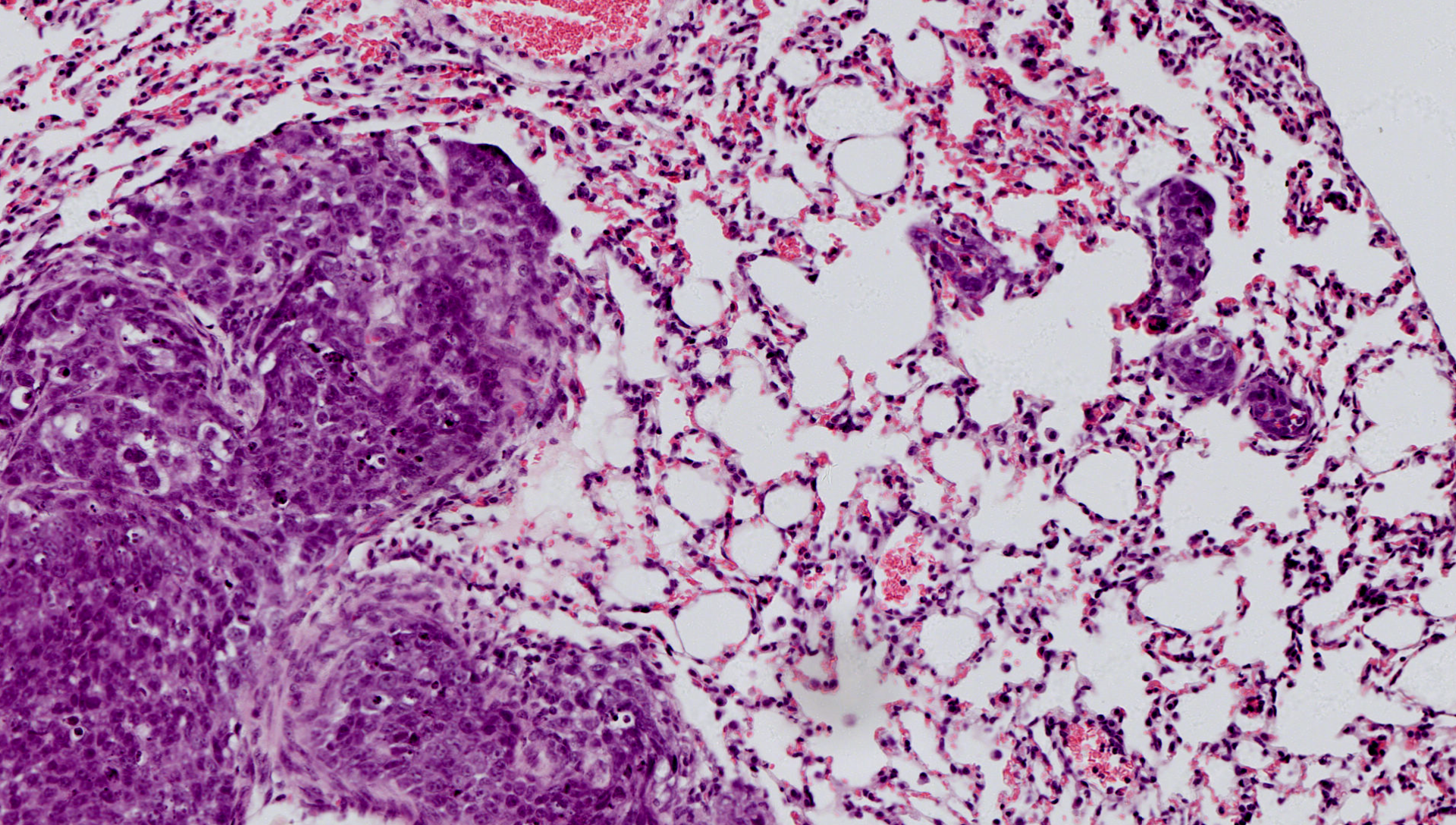

Supplement: Supplementary file 9 [file DataSheet_9.zip › Micrograph Figure 2H-Lung/Lung FbxO9 20x.tif]

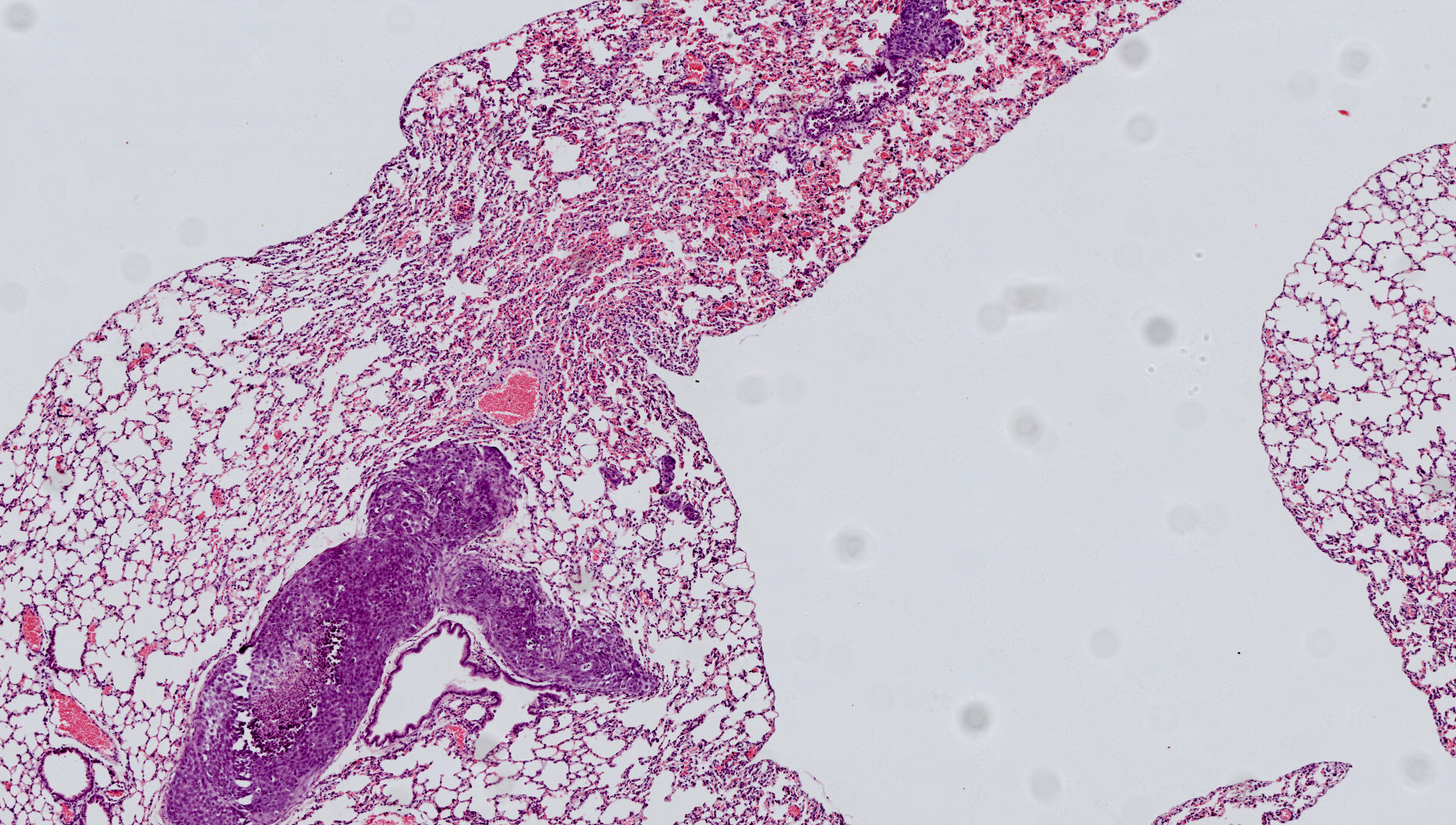

Supplement: Supplementary file 9 [file DataSheet_9.zip › Micrograph Figure 2H-Lung/Lung FbxO9 5x.tif]

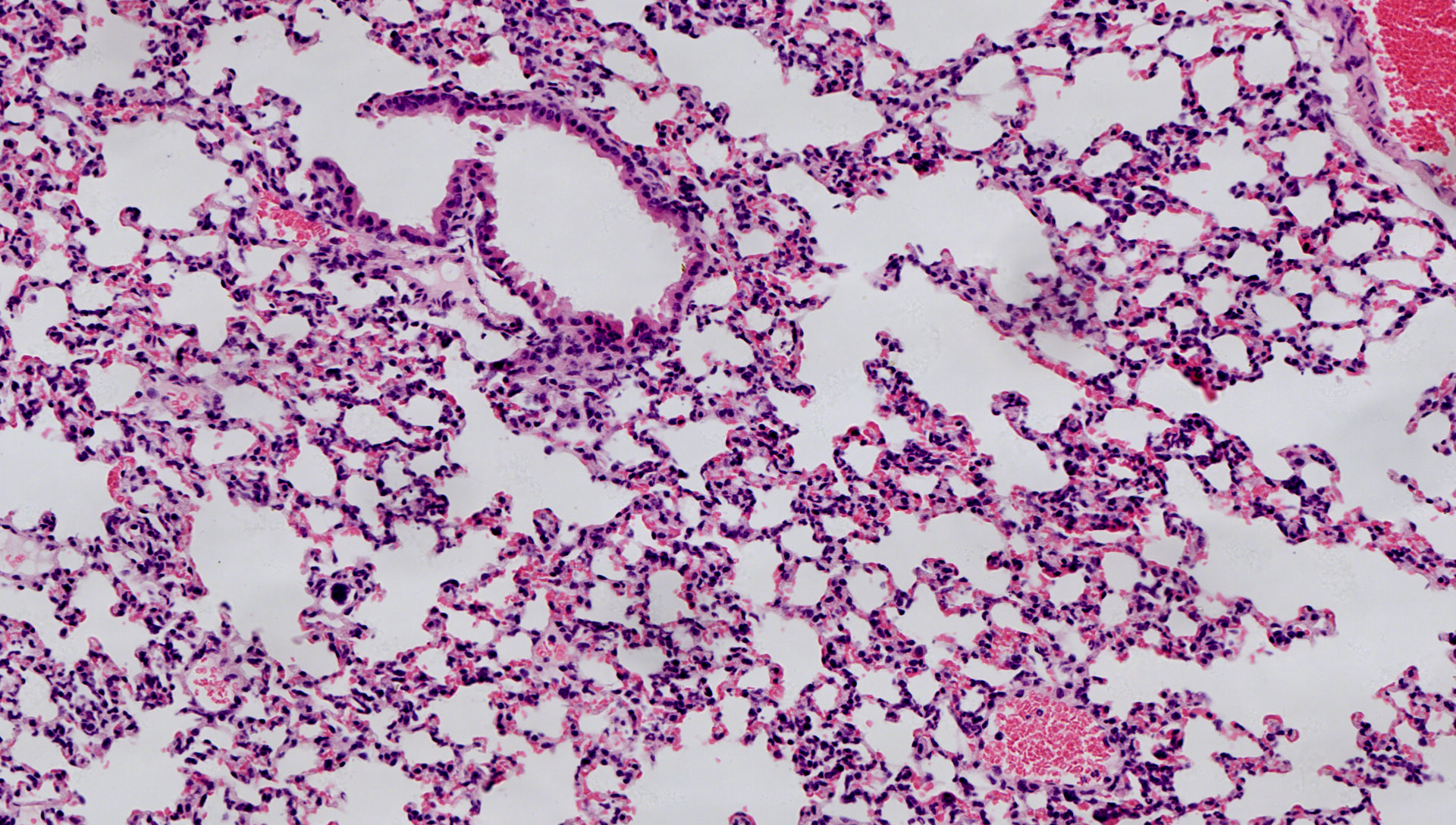

Supplement: Supplementary file 9 [file DataSheet_9.zip › Micrograph Figure 2H-Lung/Lung Vector 20x.tif]

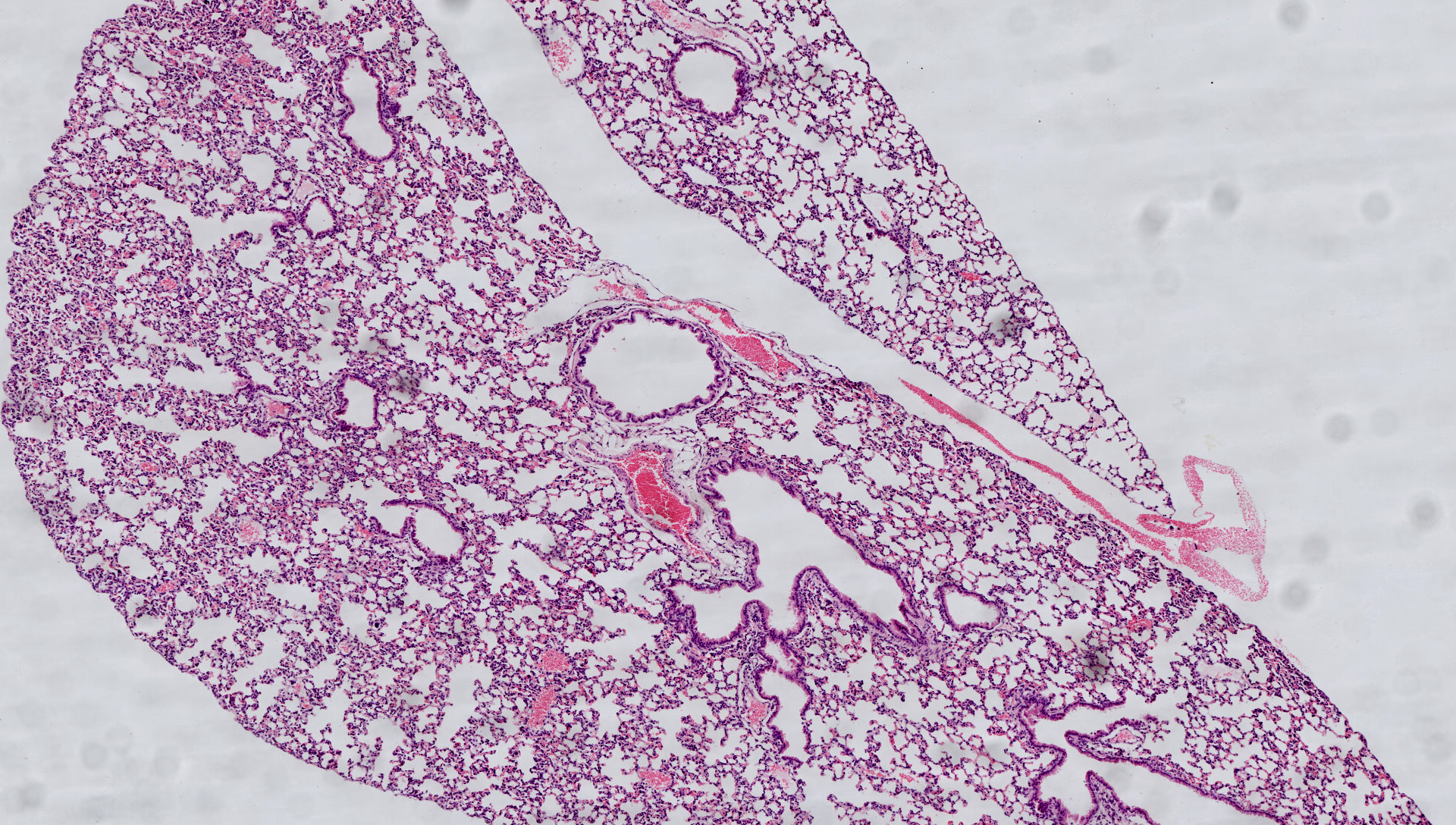

Supplement: Supplementary file 9 [file DataSheet_9.zip › Micrograph Figure 2H-Lung/Lung Vector 5x.tif]

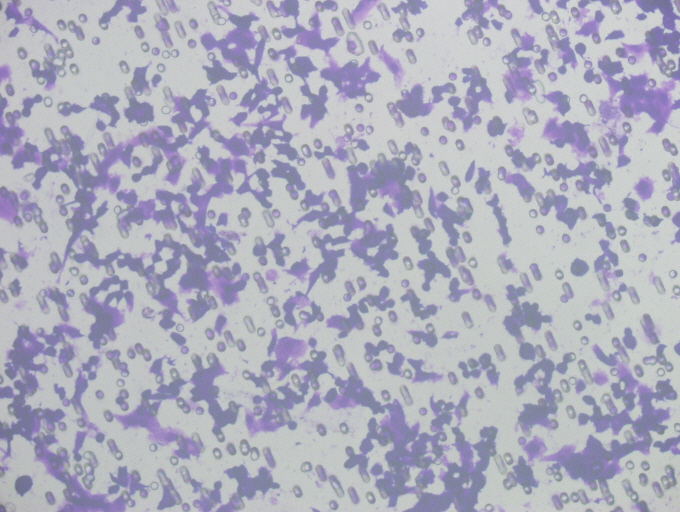

Supplement: Supplementary file 10 [file DataSheet_10.zip › Micrograph Figure 2F/HCC-LY10 FBXO9.jpg]

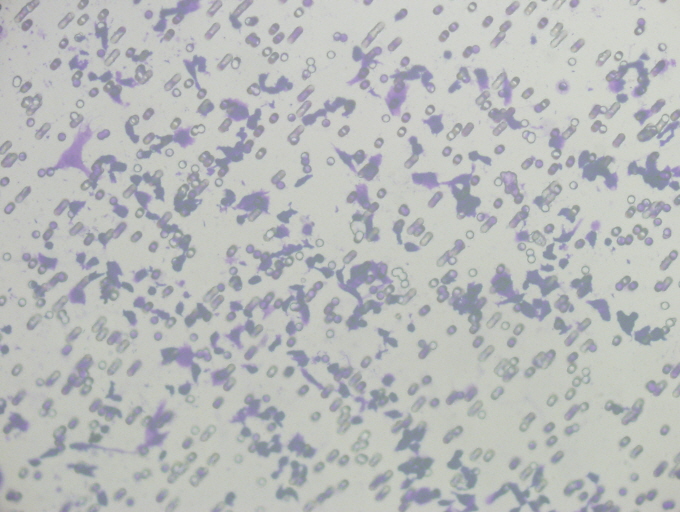

Supplement: Supplementary file 10 [file DataSheet_10.zip › Micrograph Figure 2F/HCC-LY10 Vector.jpg]

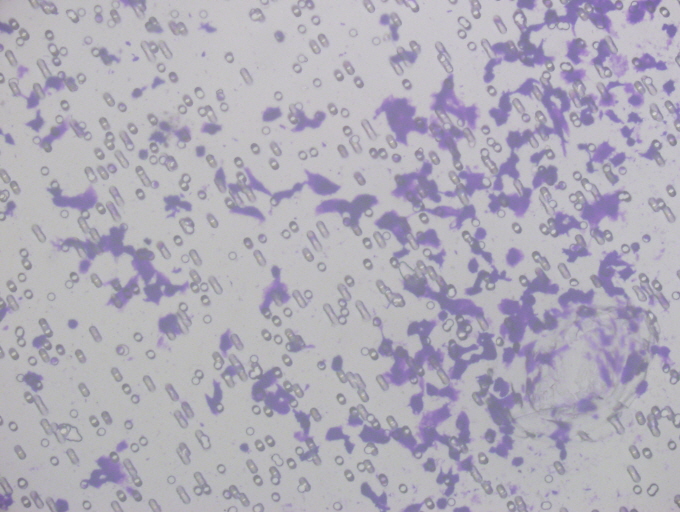

Supplement: Supplementary file 10 [file DataSheet_10.zip › Micrograph Figure 2F/LI7 FBXO9.jpg]

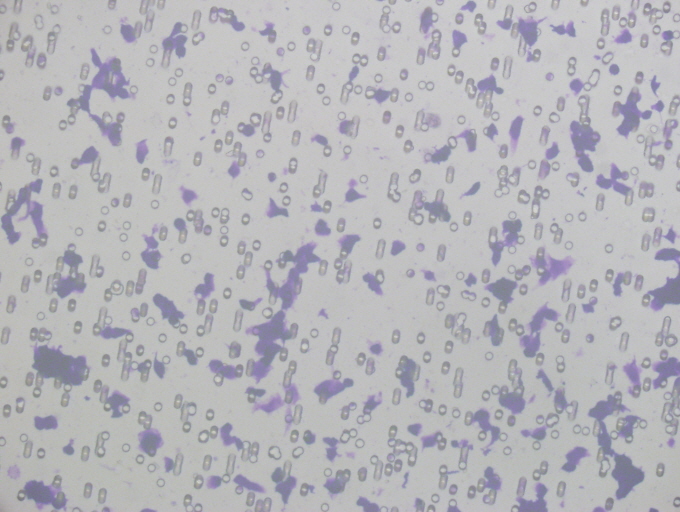

Supplement: Supplementary file 10 [file DataSheet_10.zip › Micrograph Figure 2F/LI7 VECTOR.jpg]

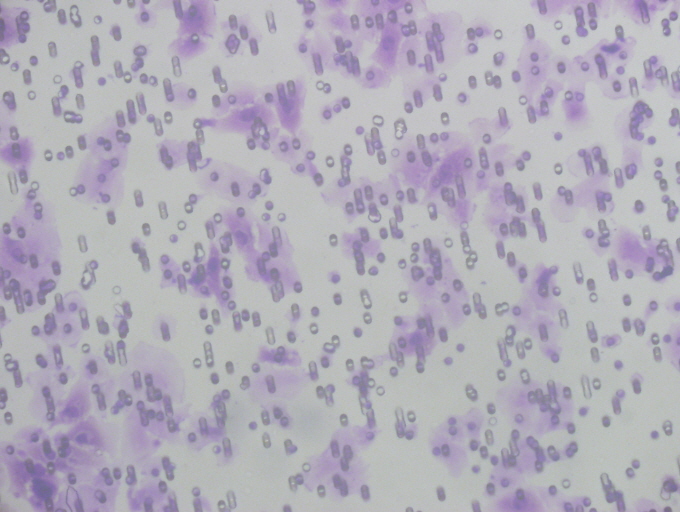

Supplement: Supplementary file 10 [file DataSheet_10.zip › Micrograph Figure 2F/LM3 nc.jpg]

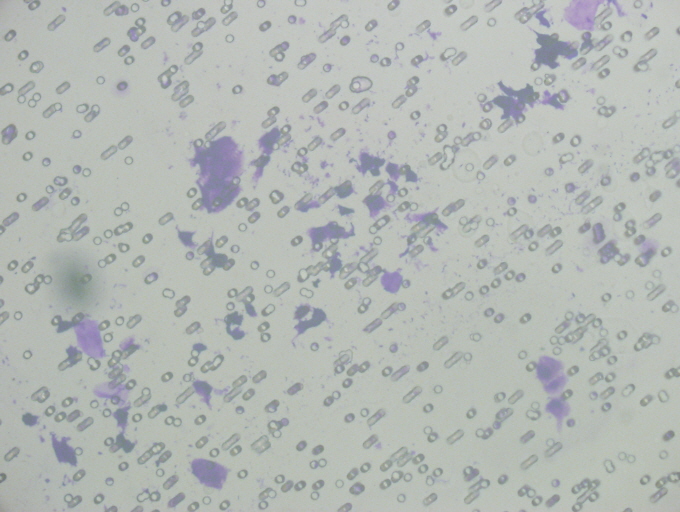

Supplement: Supplementary file 10 [file DataSheet_10.zip › Micrograph Figure 2F/LM3 shFBXO9#2.jpg]

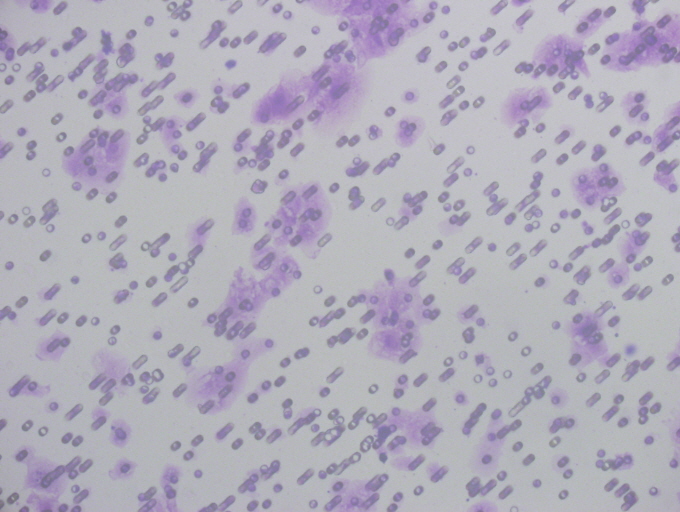

Supplement: Supplementary file 10 [file DataSheet_10.zip › Micrograph Figure 2F/PLC shFBXO9#2.jpg]

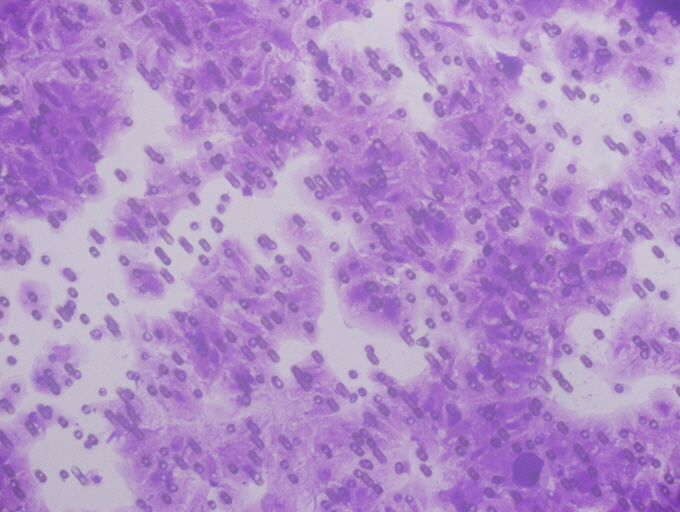

Supplement: Supplementary file 10 [file DataSheet_10.zip › Micrograph Figure 2F/PLC shNC.jpg]

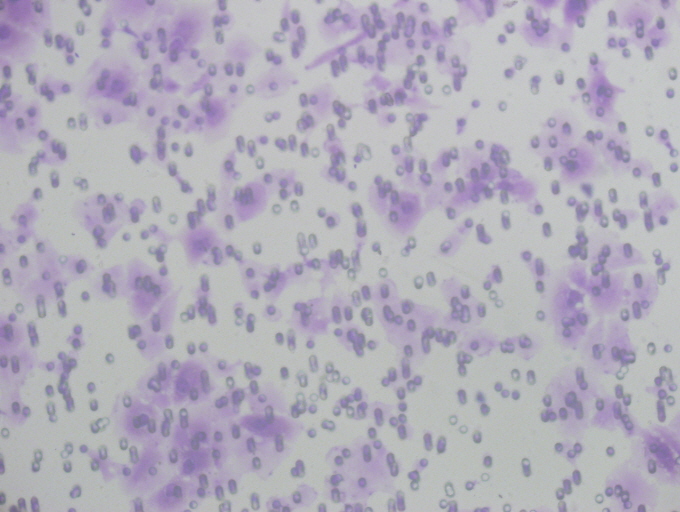

Supplement: Supplementary file 10 [file DataSheet_10.zip › Micrograph Figure 2F/lm3 mock.jpg]

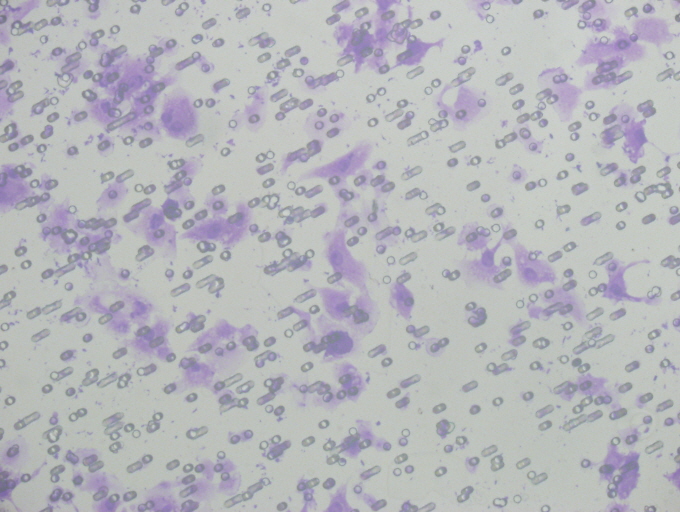

Supplement: Supplementary file 10 [file DataSheet_10.zip › Micrograph Figure 2F/lm3 shFBXO9#1.jpg]

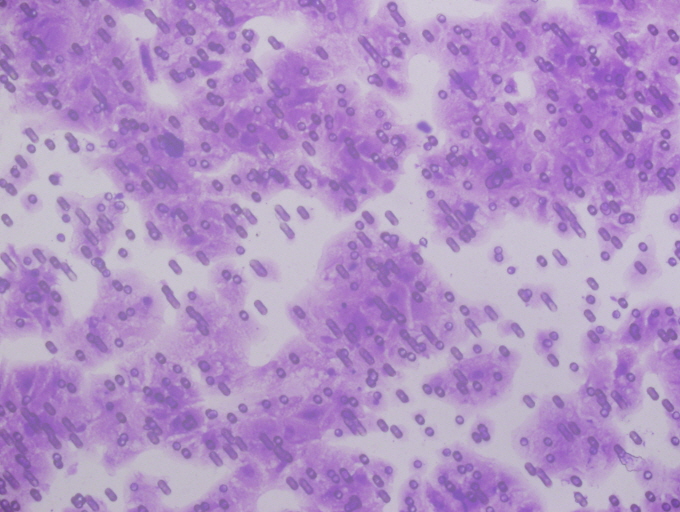

Supplement: Supplementary file 10 [file DataSheet_10.zip › Micrograph Figure 2F/plc mock.jpg]

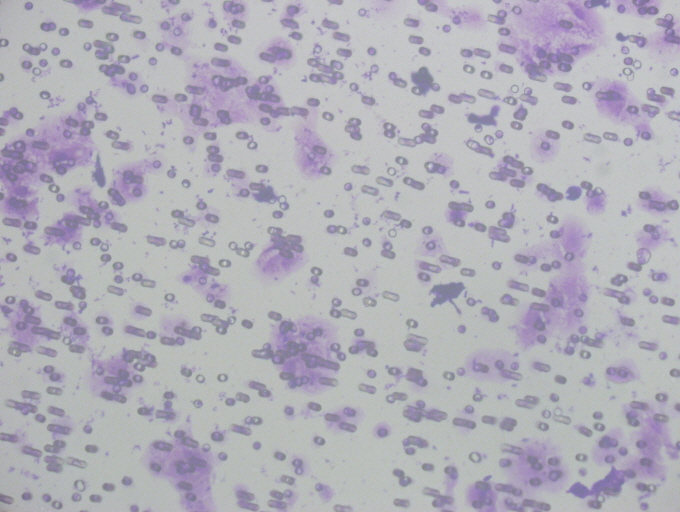

Supplement: Supplementary file 10 [file DataSheet_10.zip › Micrograph Figure 2F/plc shFBXO9#1.jpg]

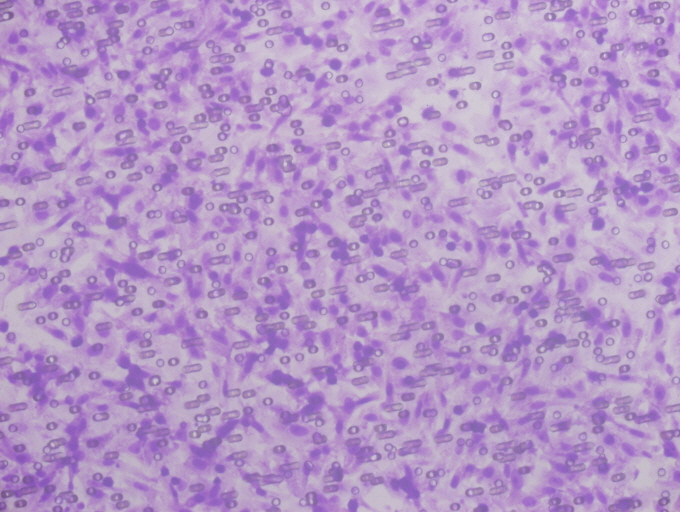

Supplement: Supplementary file 11 [file DataSheet_11.zip › Micrograph Figure 2G/HCC-LY10 FBXO9.jpg]

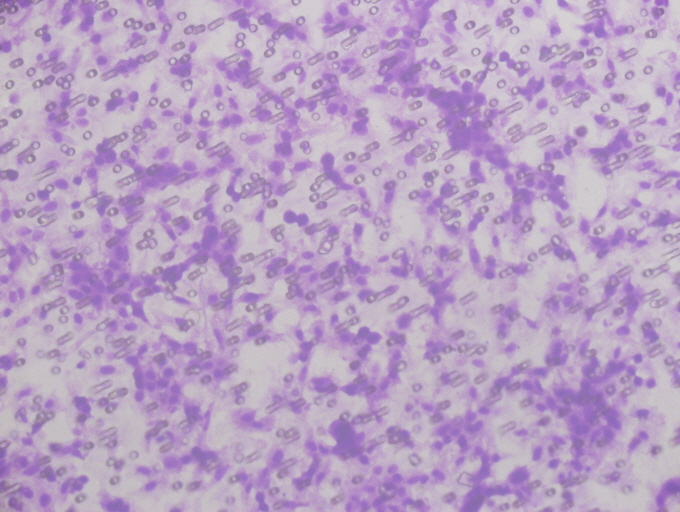

Supplement: Supplementary file 11 [file DataSheet_11.zip › Micrograph Figure 2G/HCC-LY10 Vector.jpg]

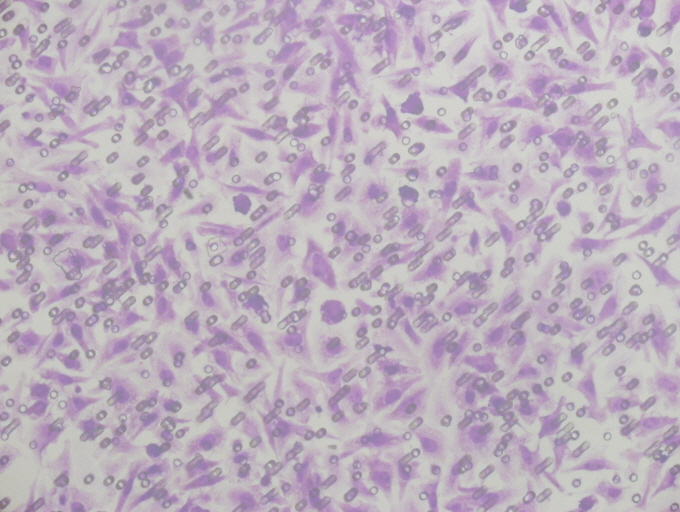

Supplement: Supplementary file 11 [file DataSheet_11.zip › Micrograph Figure 2G/LI7 FBXO9.jpg]

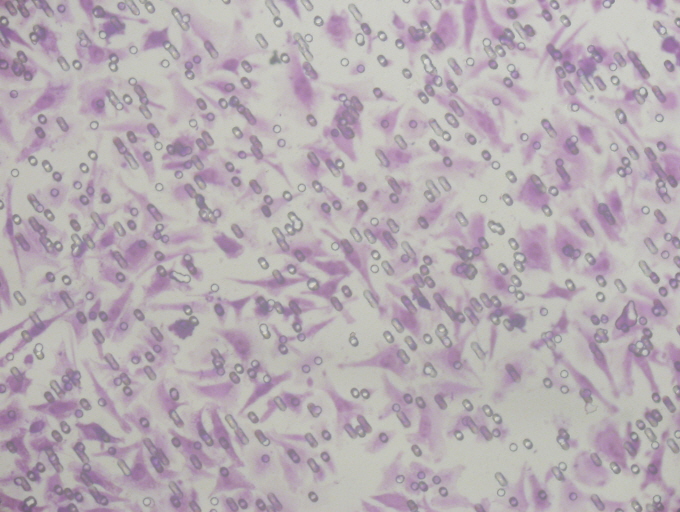

Supplement: Supplementary file 11 [file DataSheet_11.zip › Micrograph Figure 2G/LI7 Vector.jpg]

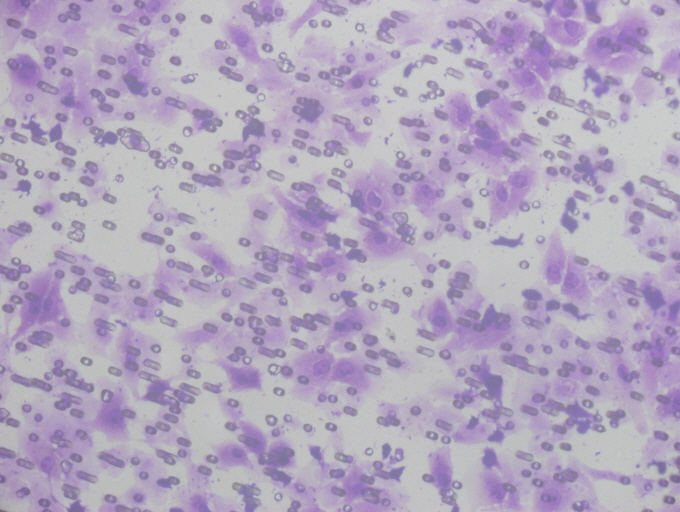

Supplement: Supplementary file 11 [file DataSheet_11.zip › Micrograph Figure 2G/lm3 mock.jpg]

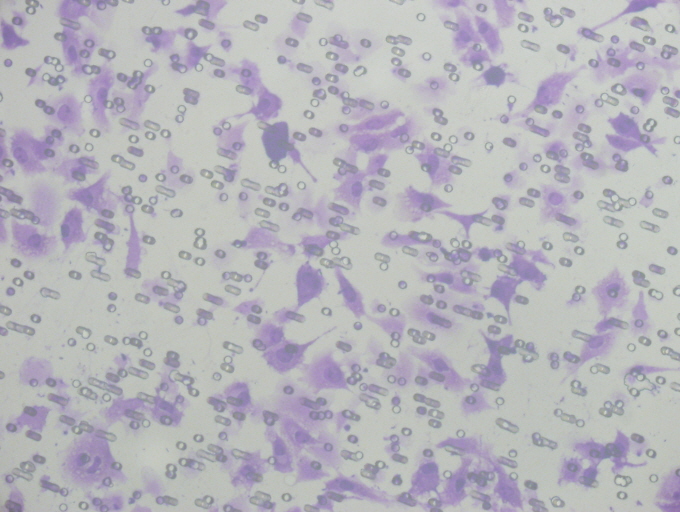

Supplement: Supplementary file 11 [file DataSheet_11.zip › Micrograph Figure 2G/lm3 shfbxo9#1.jpg]

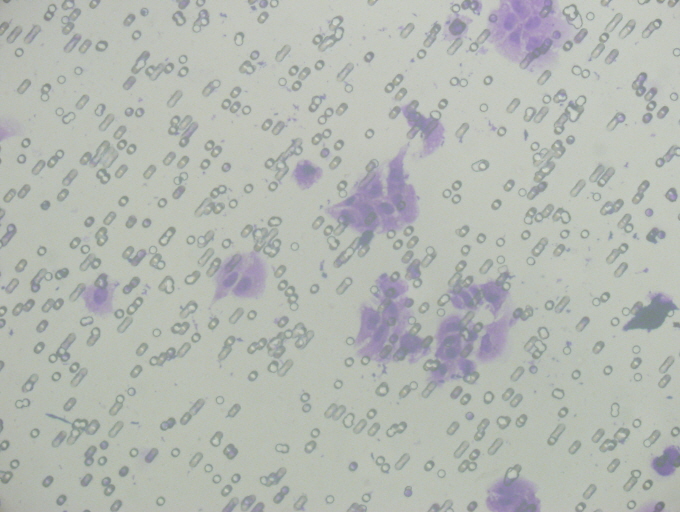

Supplement: Supplementary file 11 [file DataSheet_11.zip › Micrograph Figure 2G/lm3 shfbxo9#2.jpg]

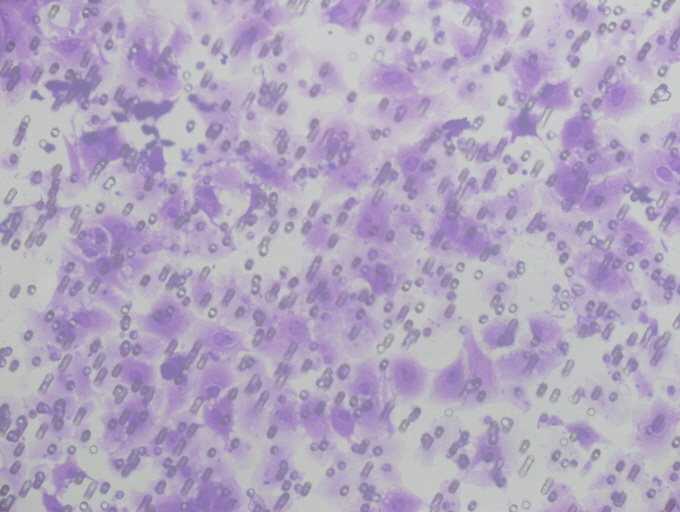

Supplement: Supplementary file 11 [file DataSheet_11.zip › Micrograph Figure 2G/lm3 shnc.jpg]

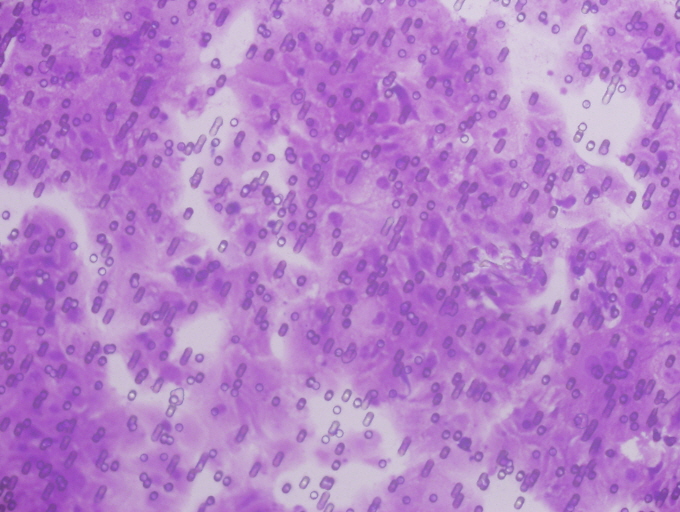

Supplement: Supplementary file 11 [file DataSheet_11.zip › Micrograph Figure 2G/plc MOCK.jpg]

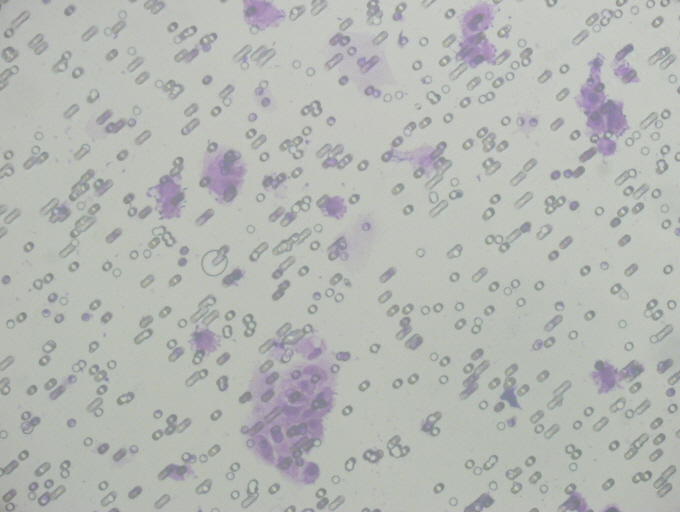

Supplement: Supplementary file 11 [file DataSheet_11.zip › Micrograph Figure 2G/plc shFBXO9#1 .jpg]

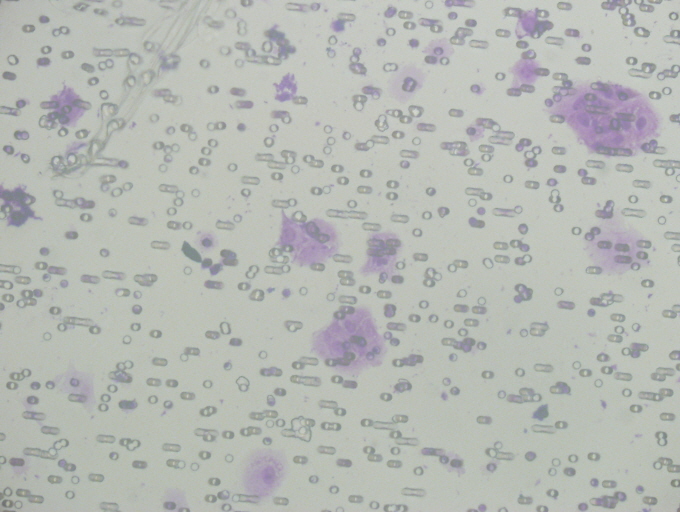

Supplement: Supplementary file 11 [file DataSheet_11.zip › Micrograph Figure 2G/plc shFBXO9#2 .jpg]

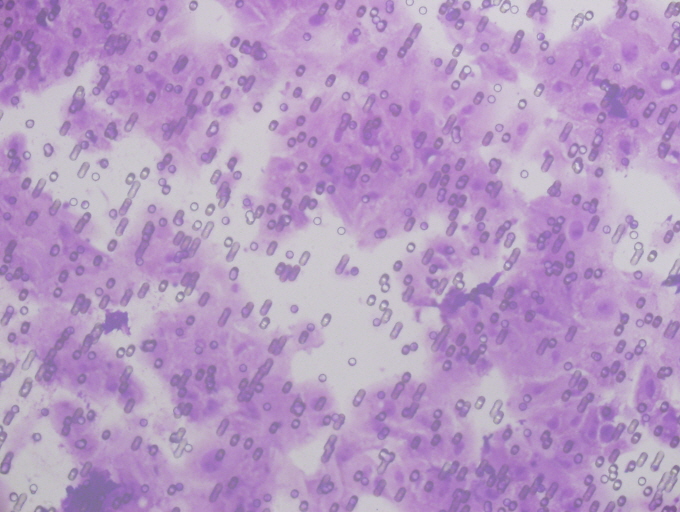

Supplement: Supplementary file 11 [file DataSheet_11.zip › Micrograph Figure 2G/plc shnc.jpg]

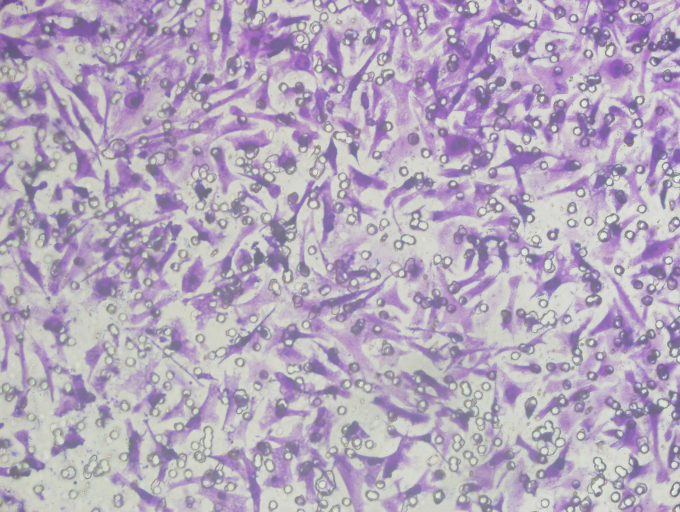

Supplement: Supplementary file 12 [file DataSheet_12.zip › Micrograph Figure 4D/Invasion/LI7/VectorshNC.tif]

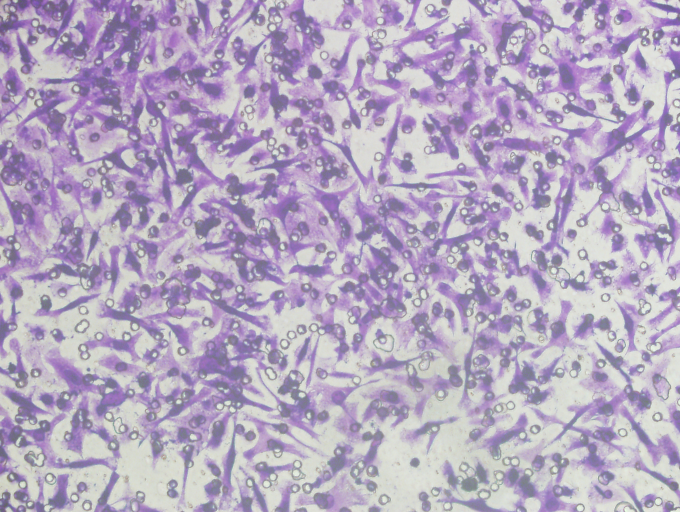

Supplement: Supplementary file 12 [file DataSheet_12.zip › Micrograph Figure 4D/Invasion/LI7/ZNF143shFBXO9#1.tif]

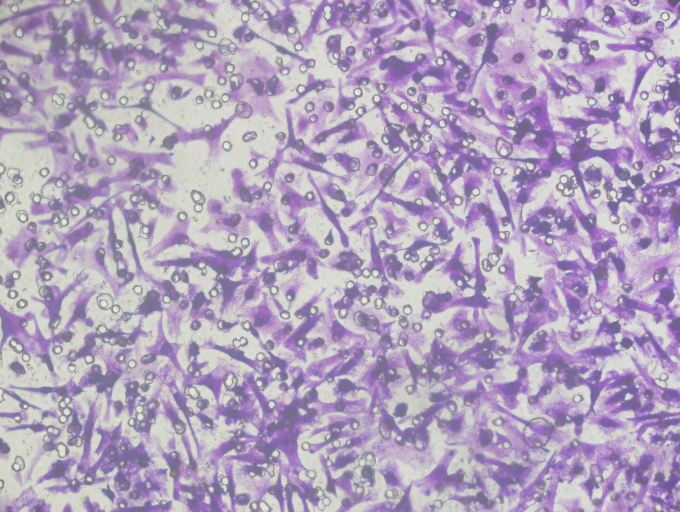

Supplement: Supplementary file 12 [file DataSheet_12.zip › Micrograph Figure 4D/Invasion/LI7/ZNF143shFBXO9#2.tif]

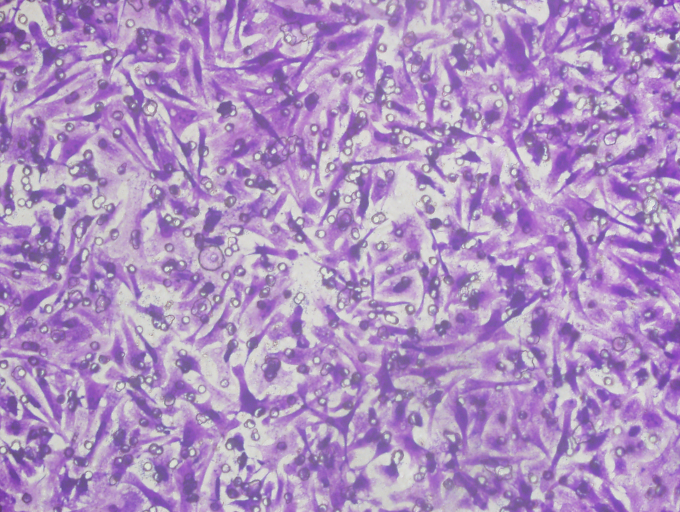

Supplement: Supplementary file 12 [file DataSheet_12.zip › Micrograph Figure 4D/Invasion/LI7/ZNF143shNC.tif]

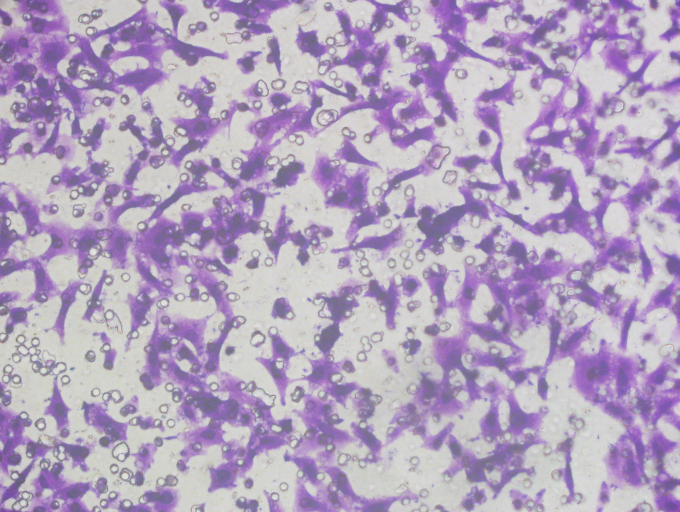

Supplement: Supplementary file 12 [file DataSheet_12.zip › Micrograph Figure 4D/Invasion/LY10/VectorshNC.tif]

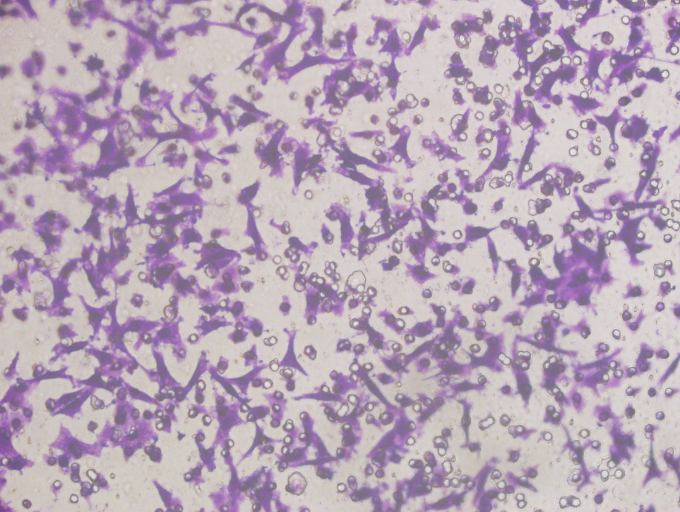

Supplement: Supplementary file 12 [file DataSheet_12.zip › Micrograph Figure 4D/Invasion/LY10/ZNF143shFBXO9#1.tif]

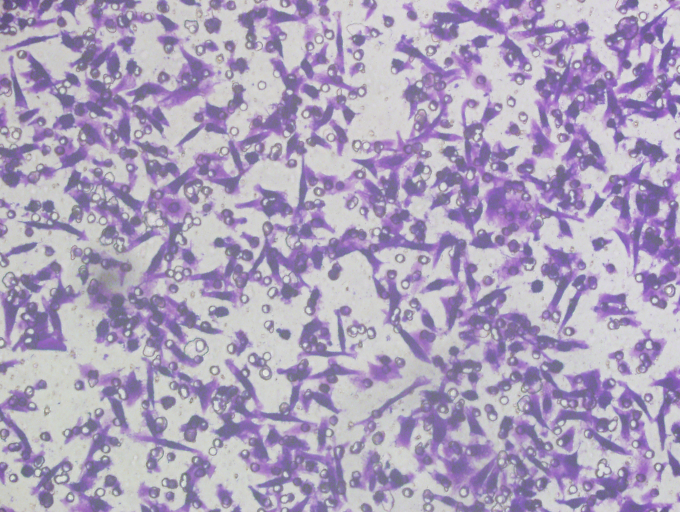

Supplement: Supplementary file 12 [file DataSheet_12.zip › Micrograph Figure 4D/Invasion/LY10/ZNF143shFBXO9#2.tif]

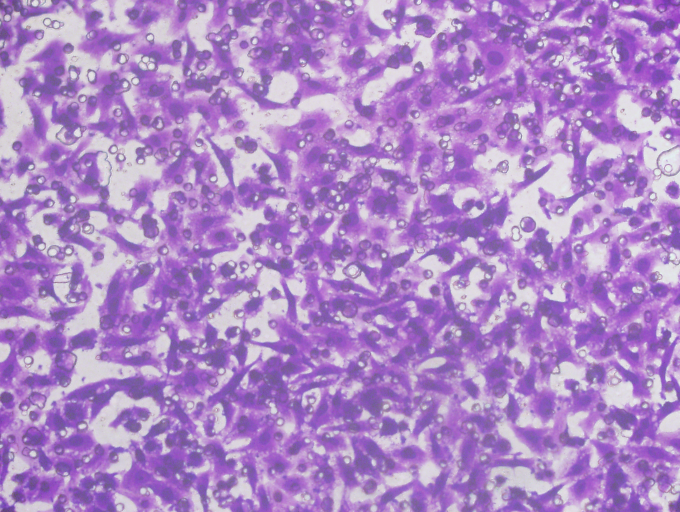

Supplement: Supplementary file 12 [file DataSheet_12.zip › Micrograph Figure 4D/Invasion/LY10/ZNF143shNC.tif]

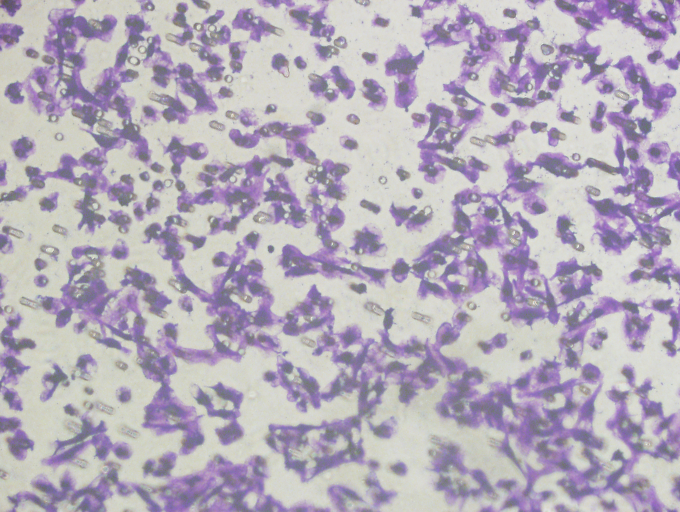

Supplement: Supplementary file 12 [file DataSheet_12.zip › Micrograph Figure 4D/Migration/li7/VectorshNC.tif]

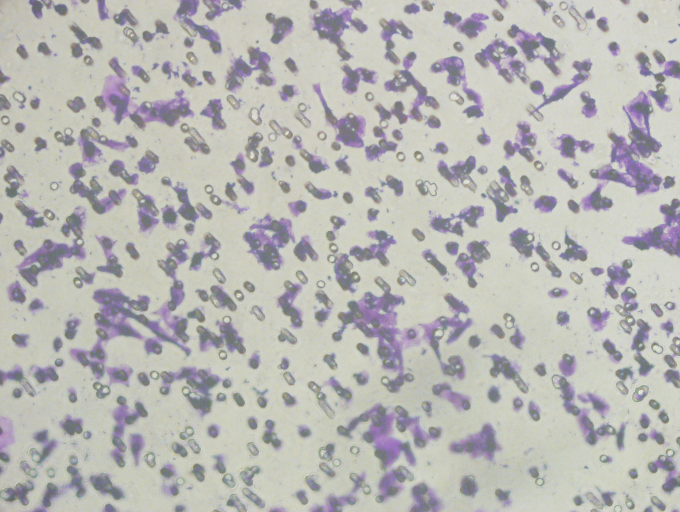

Supplement: Supplementary file 12 [file DataSheet_12.zip › Micrograph Figure 4D/Migration/li7/ZNF143shFBXO9#1.tif]

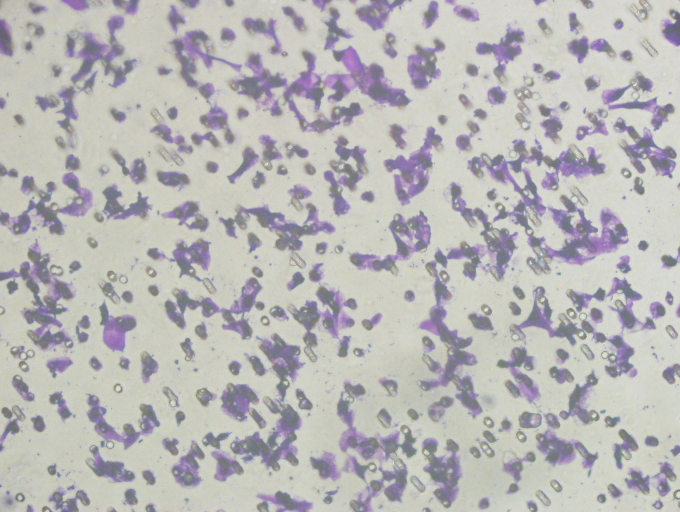

Supplement: Supplementary file 12 [file DataSheet_12.zip › Micrograph Figure 4D/Migration/li7/ZNF143shFBXO9#2.tif]

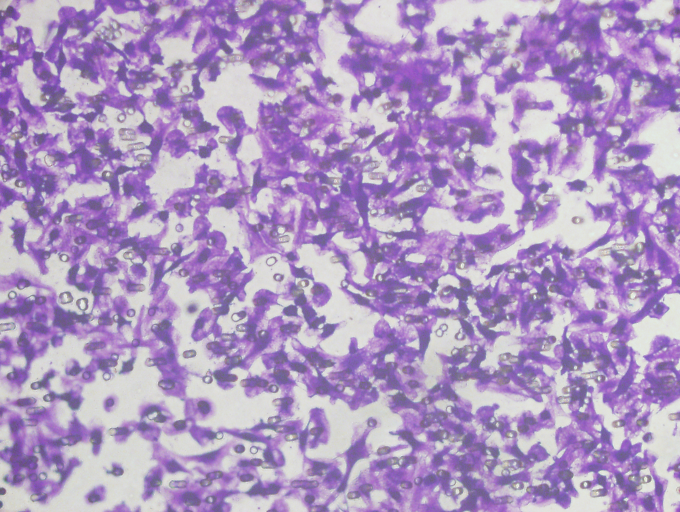

Supplement: Supplementary file 12 [file DataSheet_12.zip › Micrograph Figure 4D/Migration/li7/ZNF143shNC.tif]

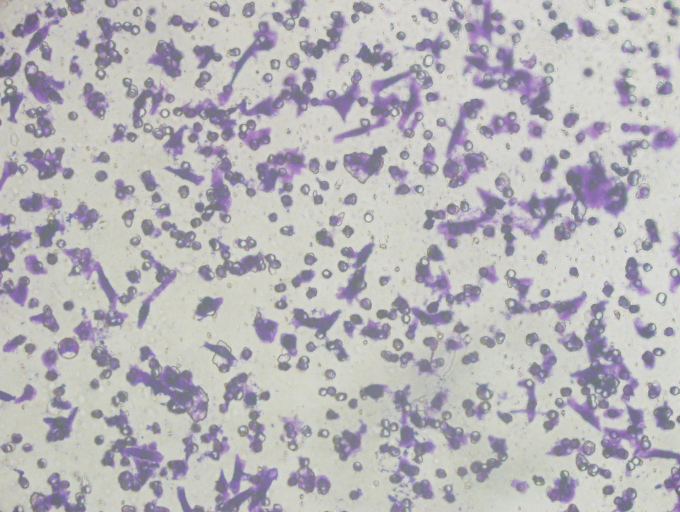

Supplement: Supplementary file 12 [file DataSheet_12.zip › Micrograph Figure 4D/Migration/ly10/VectorshNC.tif]

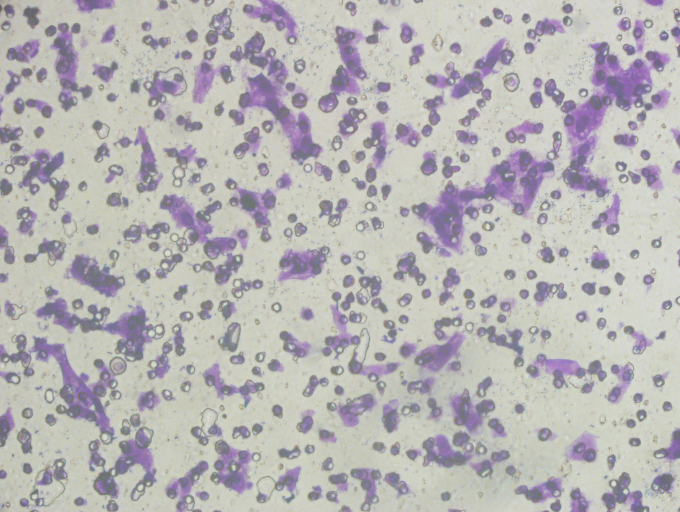

Supplement: Supplementary file 12 [file DataSheet_12.zip › Micrograph Figure 4D/Migration/ly10/ZNF143shFBXO9#1.tif]

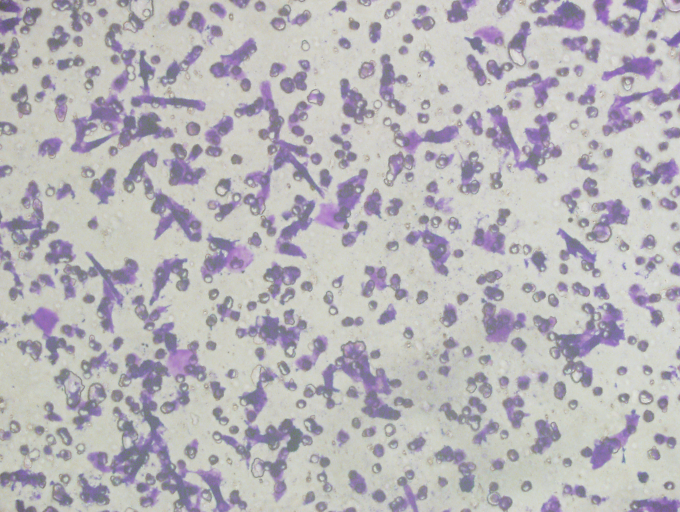

Supplement: Supplementary file 12 [file DataSheet_12.zip › Micrograph Figure 4D/Migration/ly10/ZNF143shFBXO9#2.tif]

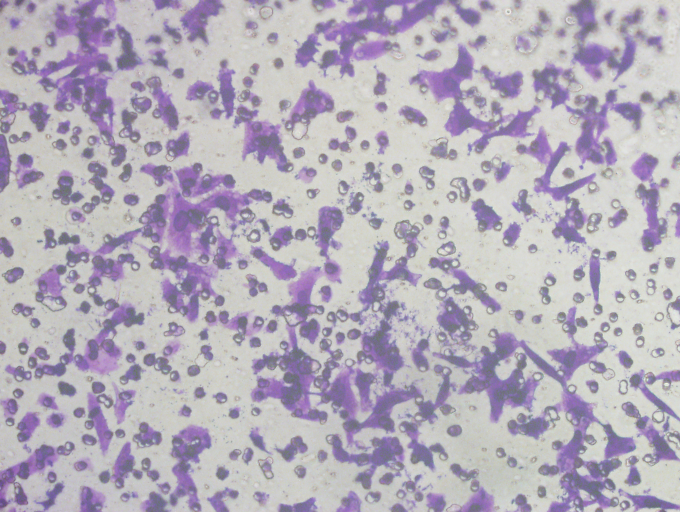

Supplement: Supplementary file 12 [file DataSheet_12.zip › Micrograph Figure 4D/Migration/ly10/ZNF143shNC.tif]

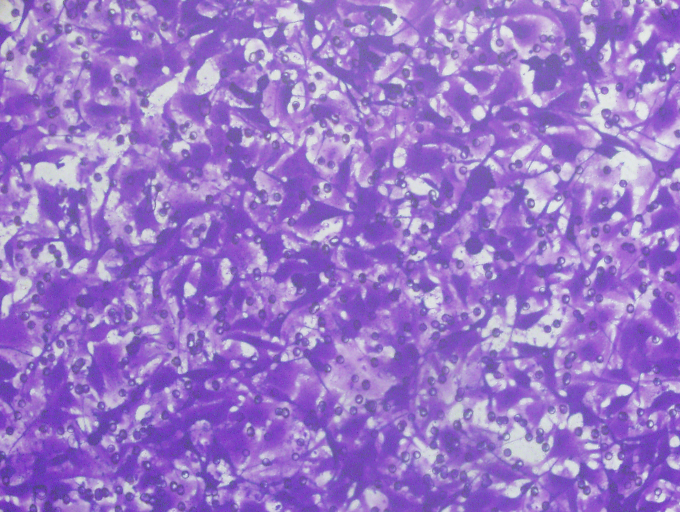

Supplement: Supplementary file 13 [file DataSheet_13.zip › Micrograph Figure 4H/Invasion/MOCK.tif]

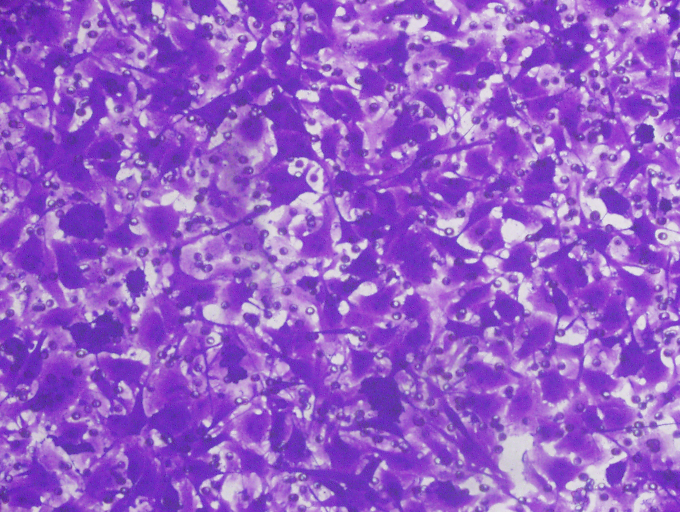

Supplement: Supplementary file 13 [file DataSheet_13.zip › Micrograph Figure 4H/Invasion/NC.tif]

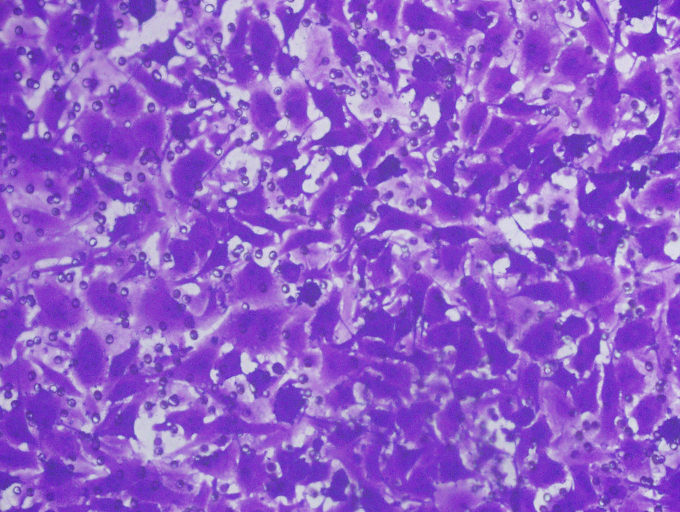

Supplement: Supplementary file 13 [file DataSheet_13.zip › Micrograph Figure 4H/Invasion/shZNF143#1FBXO9.tif]

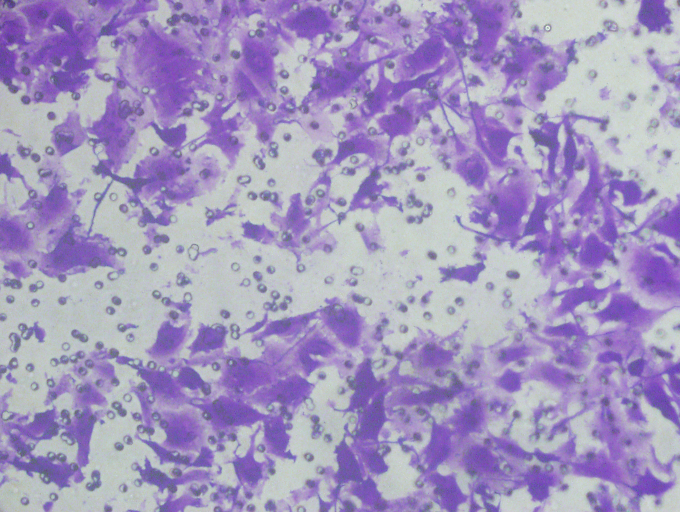

Supplement: Supplementary file 13 [file DataSheet_13.zip › Micrograph Figure 4H/Invasion/shZNF143#1Vector.tif]

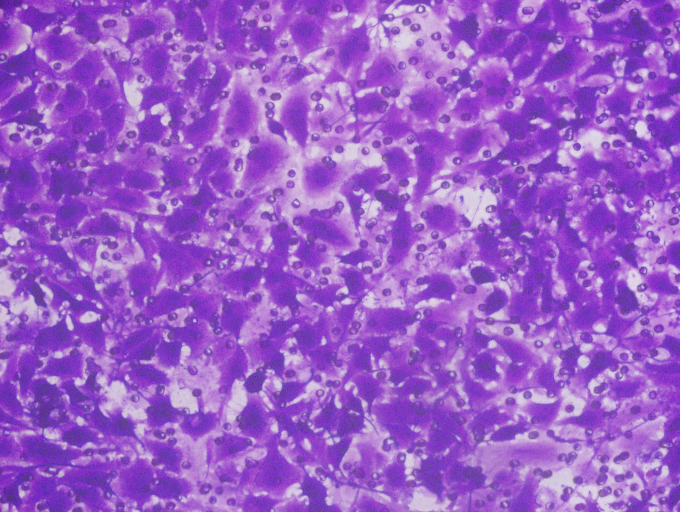

Supplement: Supplementary file 13 [file DataSheet_13.zip › Micrograph Figure 4H/Invasion/shZNF143#2FBXO9.tif]

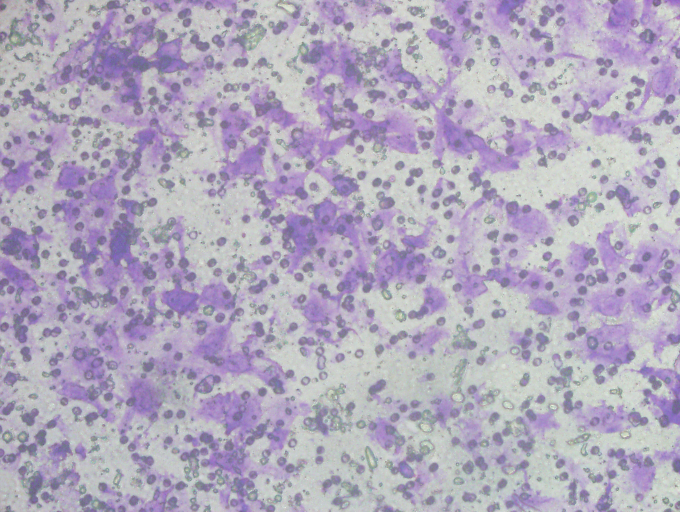

Supplement: Supplementary file 13 [file DataSheet_13.zip › Micrograph Figure 4H/Invasion/shZNF143#2Vector.tif]

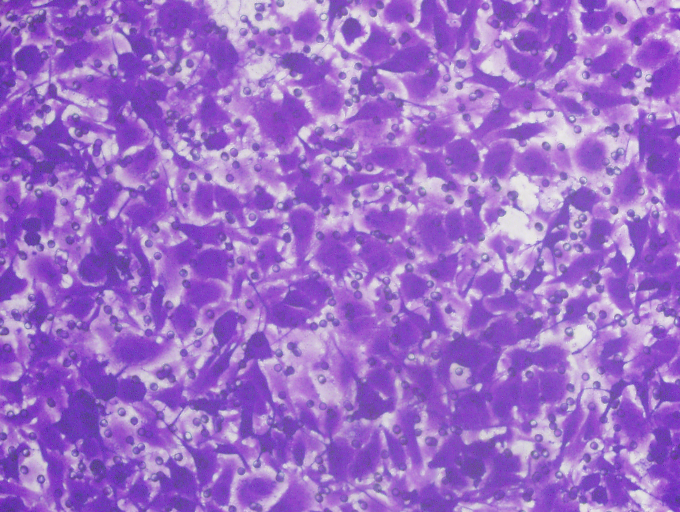

Supplement: Supplementary file 13 [file DataSheet_13.zip › Micrograph Figure 4H/Migration/MOCK.tif]

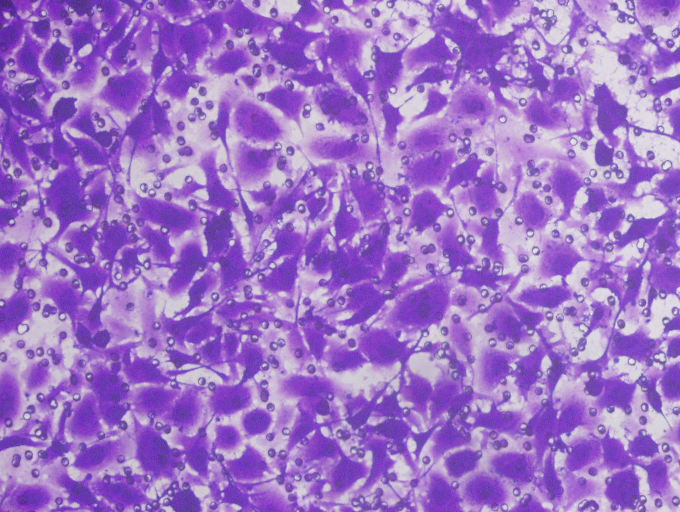

Supplement: Supplementary file 13 [file DataSheet_13.zip › Micrograph Figure 4H/Migration/NC.tif]

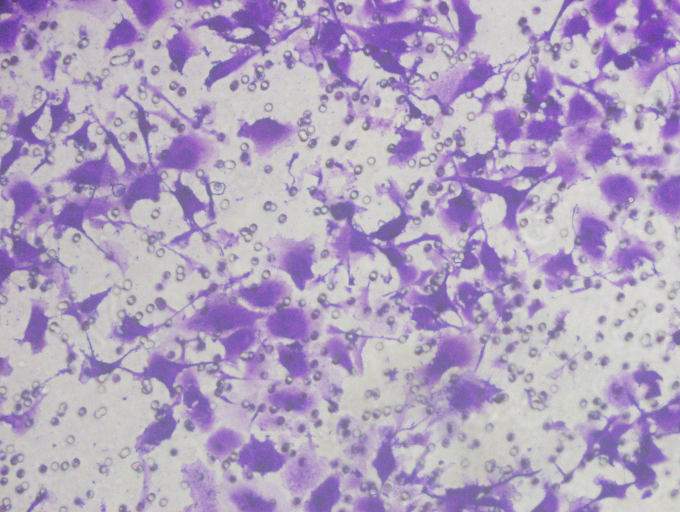

Supplement: Supplementary file 13 [file DataSheet_13.zip › Micrograph Figure 4H/Migration/SHZNF143#2VECTOR.tif]

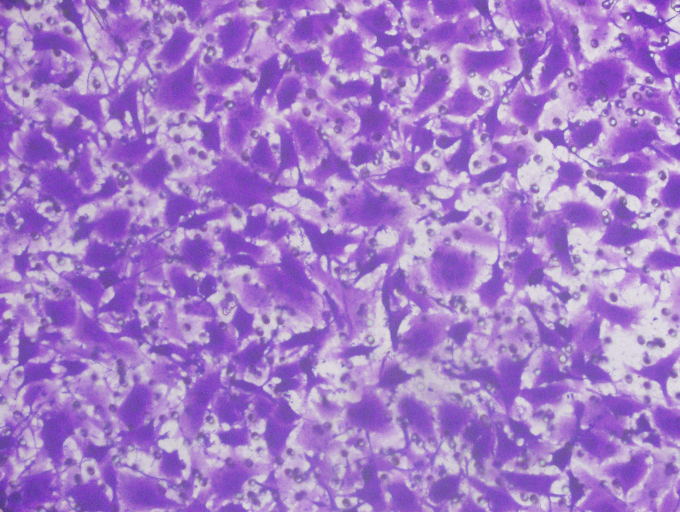

Supplement: Supplementary file 13 [file DataSheet_13.zip › Micrograph Figure 4H/Migration/shZNF143#1FBXO9.tif]

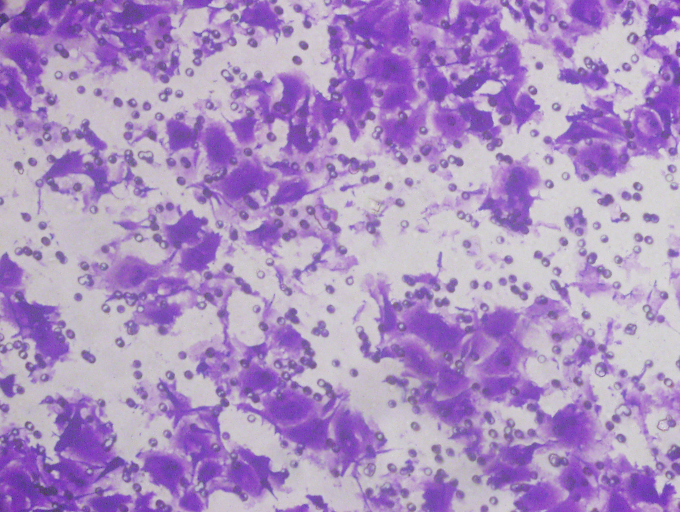

Supplement: Supplementary file 13 [file DataSheet_13.zip › Micrograph Figure 4H/Migration/shZNF143#1VECTOR.tif]

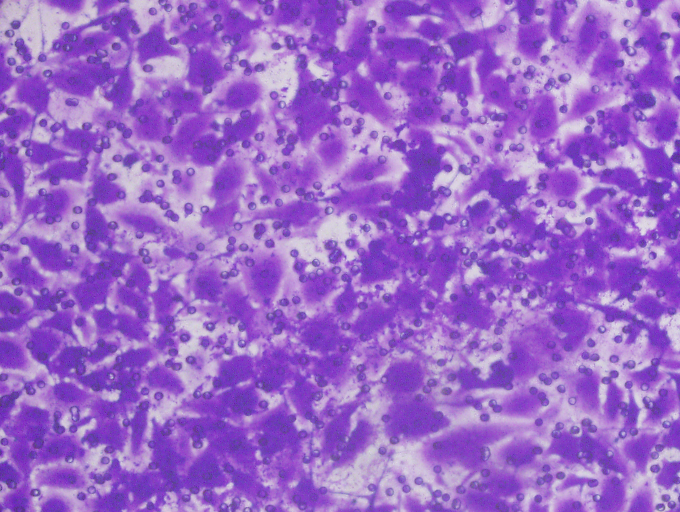

Supplement: Supplementary file 13 [file DataSheet_13.zip › Micrograph Figure 4H/Migration/shZNF143#2FBXO9.tif]

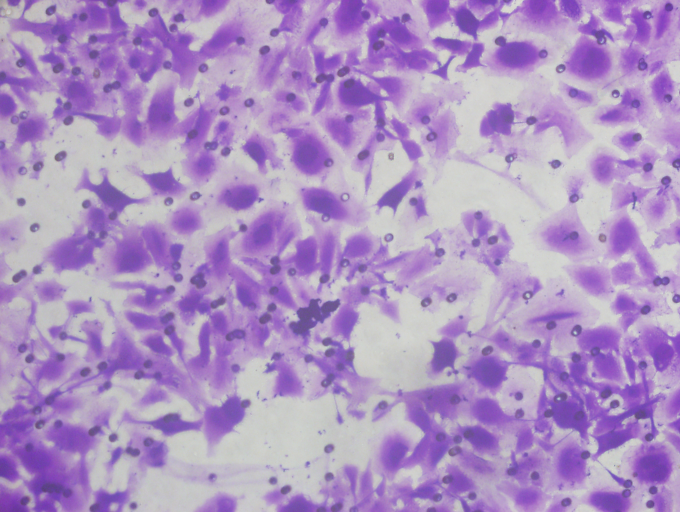

Supplement: Supplementary file 14 [file DataSheet_14.zip › Micrograph Figure S7/D/LM3 FBXW7.tif]

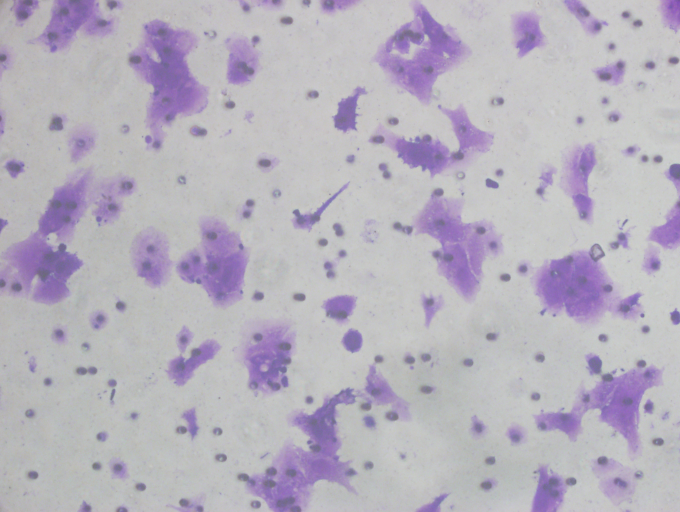

Supplement: Supplementary file 14 [file DataSheet_14.zip › Micrograph Figure S7/D/PLCPRF5FBXW7.tif]

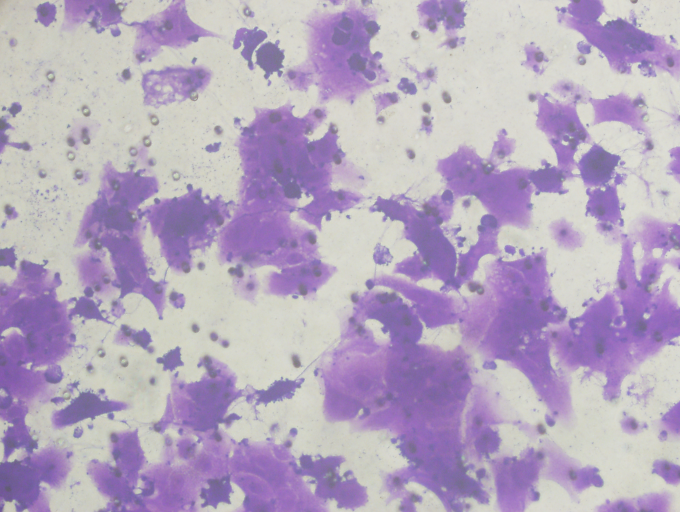

Supplement: Supplementary file 14 [file DataSheet_14.zip › Micrograph Figure S7/D/PLCPRF5Vector.tif]

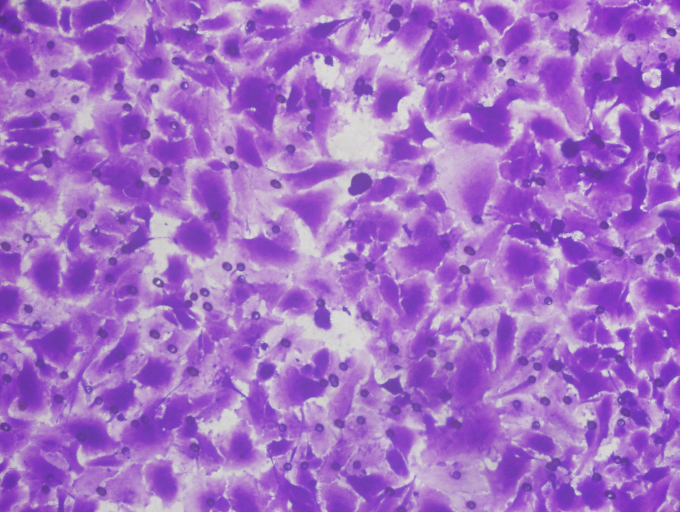

Supplement: Supplementary file 14 [file DataSheet_14.zip › Micrograph Figure S7/D/lm3 vector.tif]

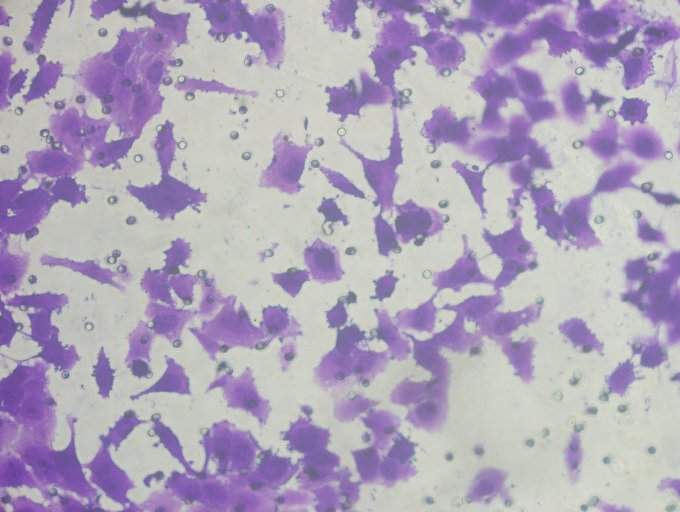

Supplement: Supplementary file 14 [file DataSheet_14.zip › Micrograph Figure S7/E/lm3 fbxw7.tif]

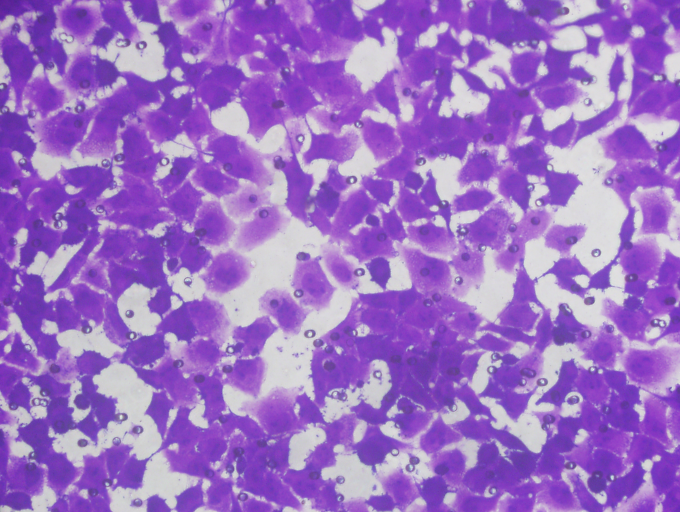

Supplement: Supplementary file 14 [file DataSheet_14.zip › Micrograph Figure S7/E/lm3 vector.tif]

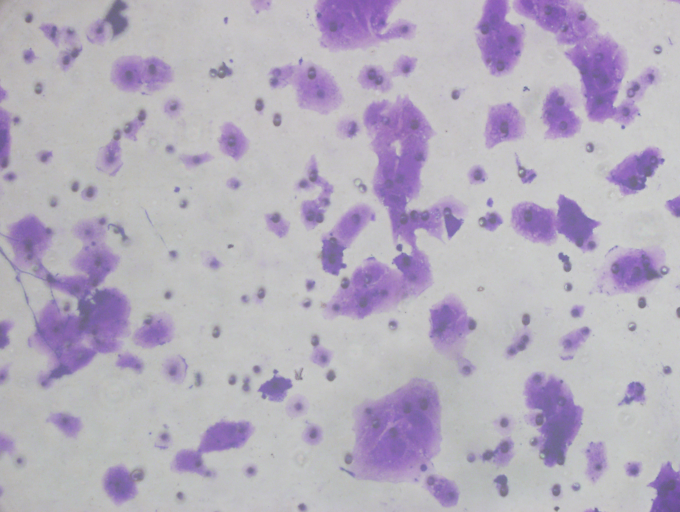

Supplement: Supplementary file 14 [file DataSheet_14.zip › Micrograph Figure S7/E/plc fbxw7.tif]

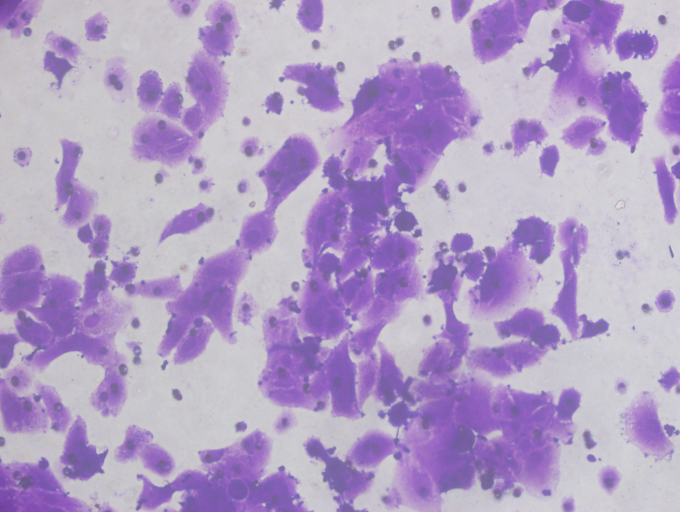

Supplement: Supplementary file 14 [file DataSheet_14.zip › Micrograph Figure S7/E/plc vector.tif]
