# Supplementary material for: FBXO9 Mediates the Cancer-Promoting Effects of ZNF143 by Degrading FBXW7 and Facilitates Drug Resistance in Hepatocellular Carcinoma
Source: Front Oncol. 2022 Jun 30;12:930220. doi: 10.3389/fonc.2022.930220 (PMC9280481; doi:10.3389/fonc.2022.930220)
Supplement: Supplementary file 15 [file DataSheet_15.pdf]

FBXO9 mediates the cancer-promoting effects of ZNF143 by degrading FBXW7 and facilitates drug resistance in hepatocellular carcinoma

Original gels

Figure 1F

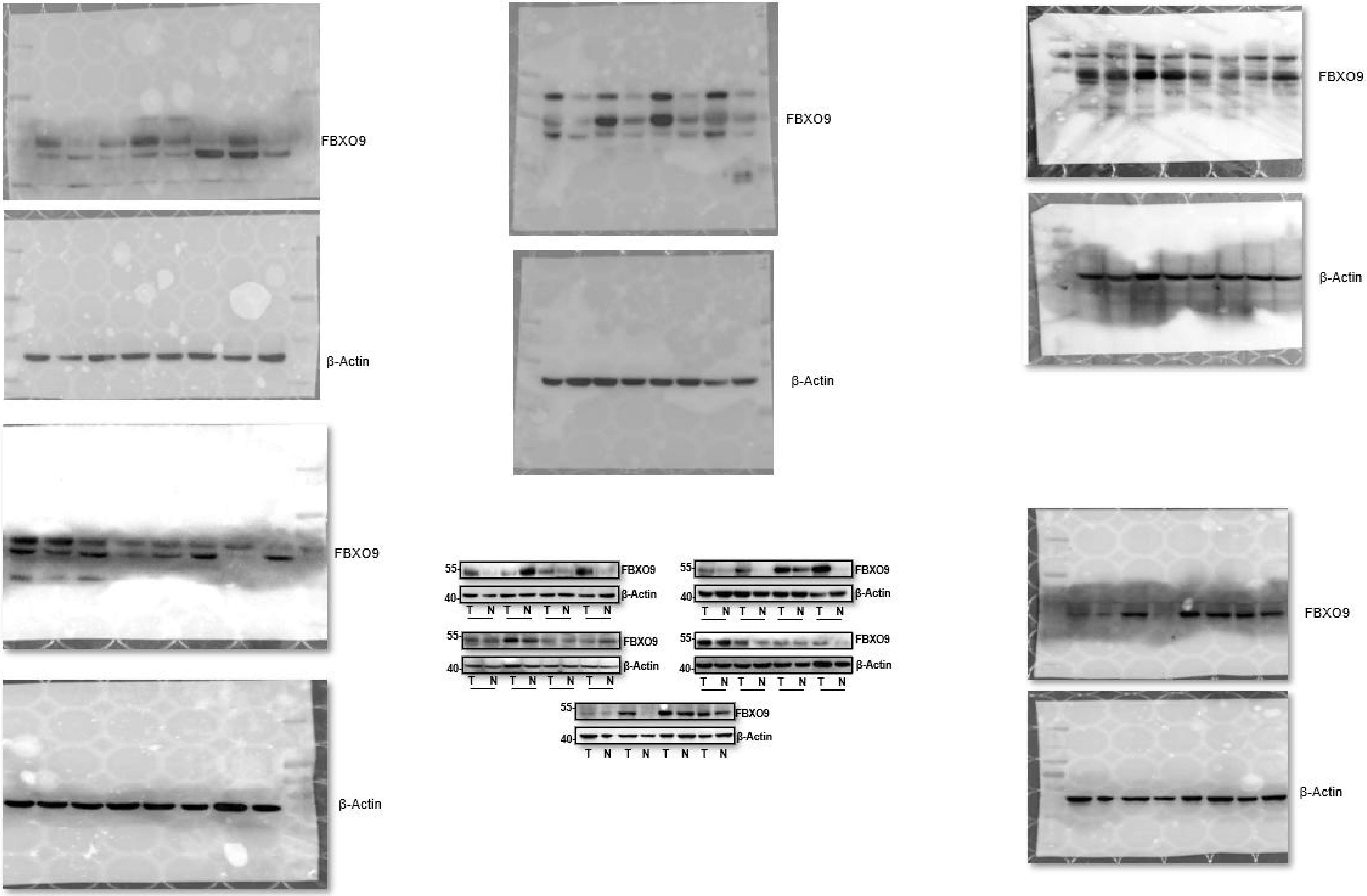
FBXO9
β-Actin
FBXO9
β-Actin
FBXO9
β-Actin
FBXO9
β-Actin
FBXO9
β-Actin
55
FBXO9
40
β-Actin
T N T N T N T N
55
FBXO9
40
β-Actin
T N T N T N T N
55
FBXO9
40
β-Actin
T N T N T N T N
55
FBXO9
40
β-Actin
T N T N T N T N
55
FBXO9
40
β-Actin
T N T N T N T N
FBXO9
β-Actin

Figure S1B

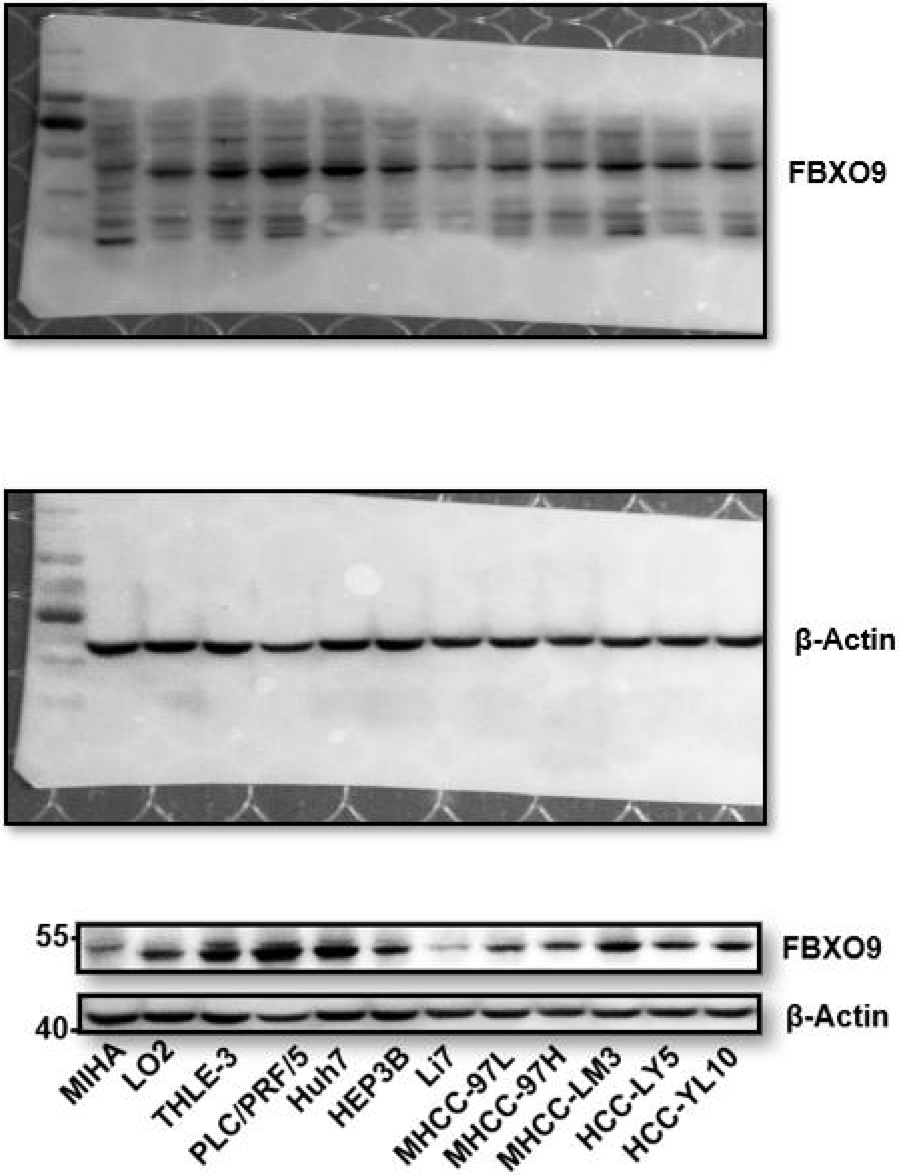

FBXO9
β-Actin
55
40
FBXO9
β-Actin
MIHA
LO2
THLE-3
PLC/PRF/5
Huh7
HEP3B
Li7
MHCC-97L
MHCC-97H
MHCC-LM3
HCC-LY5
HCC-YL10

Figure 2B

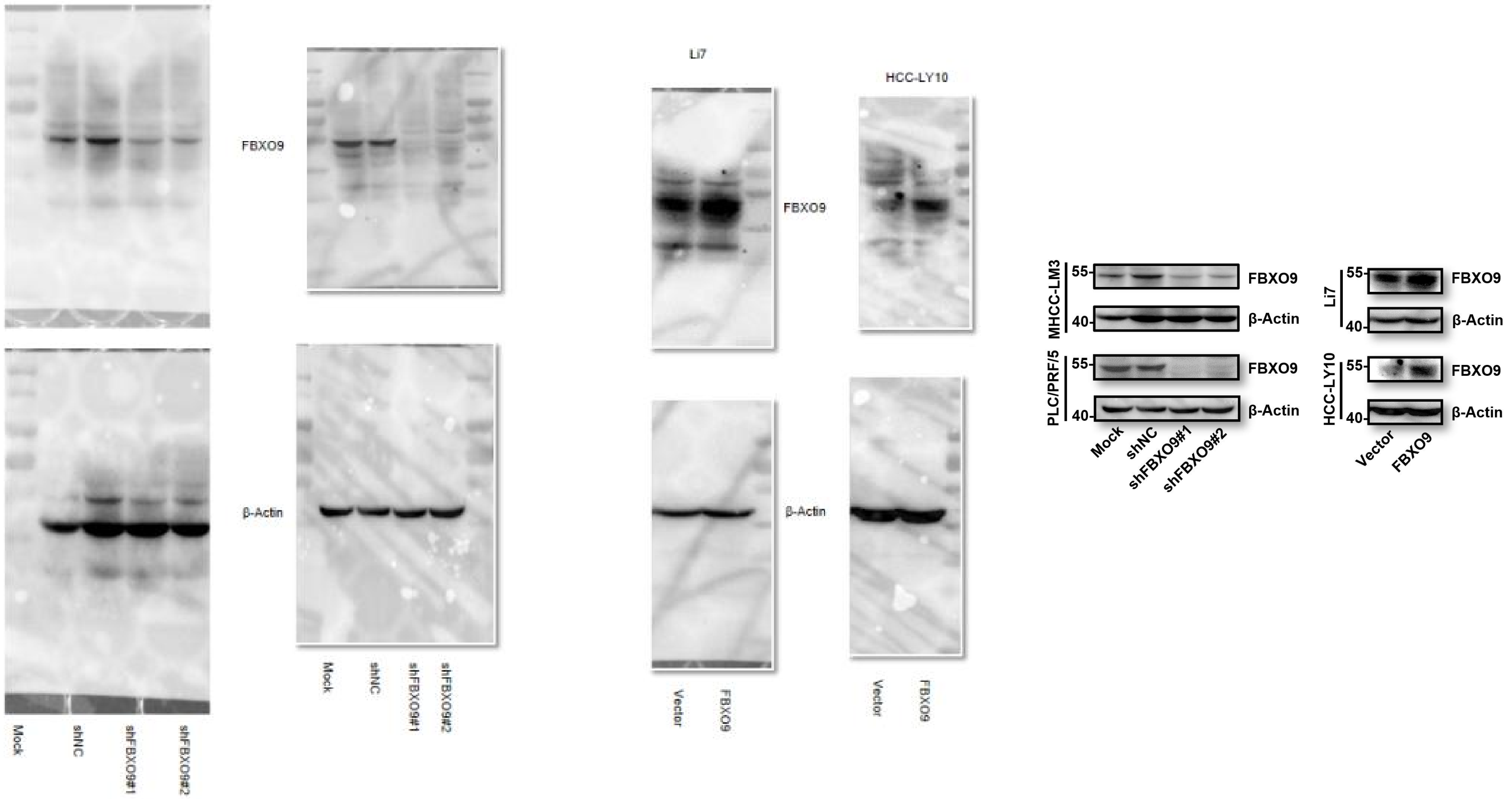

FBXO9
β-Actin
Mock
shNC
shFBXO9#1
shFBXO9#2
Li7
HCC-LY10
Vector
FBXO9
MHCC-LM3
PLC/PRF/5
55
40

Figure3C

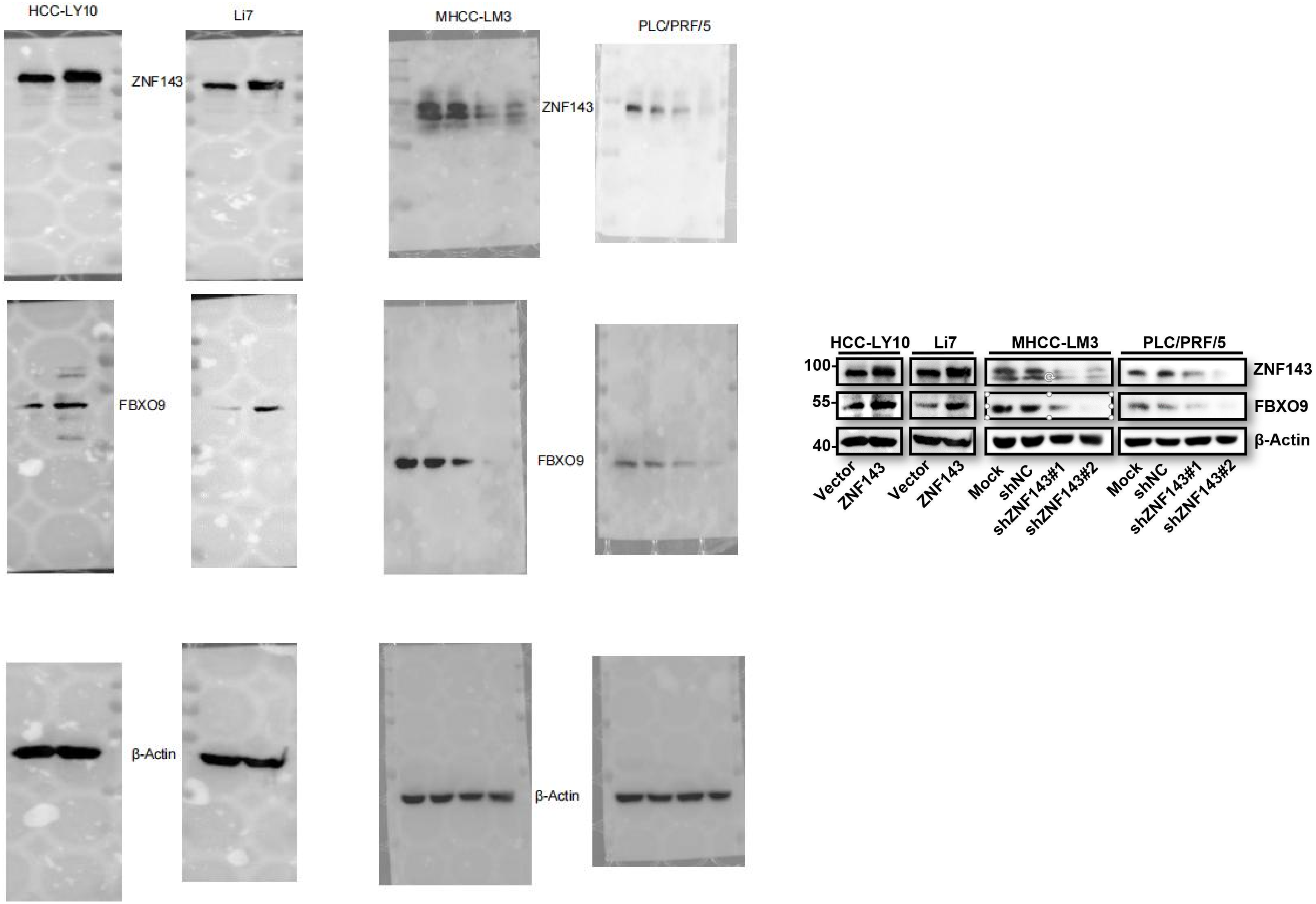

HCC-LY10
Li7
MHCC-LM3
PLC/PRF/5
ZNF143
ZNF143
FBXO9
FBXO9
β-Actin
β-Actin
HCC-LY10
Li7
MHCC-LM3
PLC/PRF/5
100
55
40
ZNF143
FBXO9
β-Actin
Vector
ZNF143
Vector
ZNF143
Mock
shNC
shZNF143#1
shZNF143#2
Mock
shNC
shZNF143#1
shZNF143#2

Figure 3E

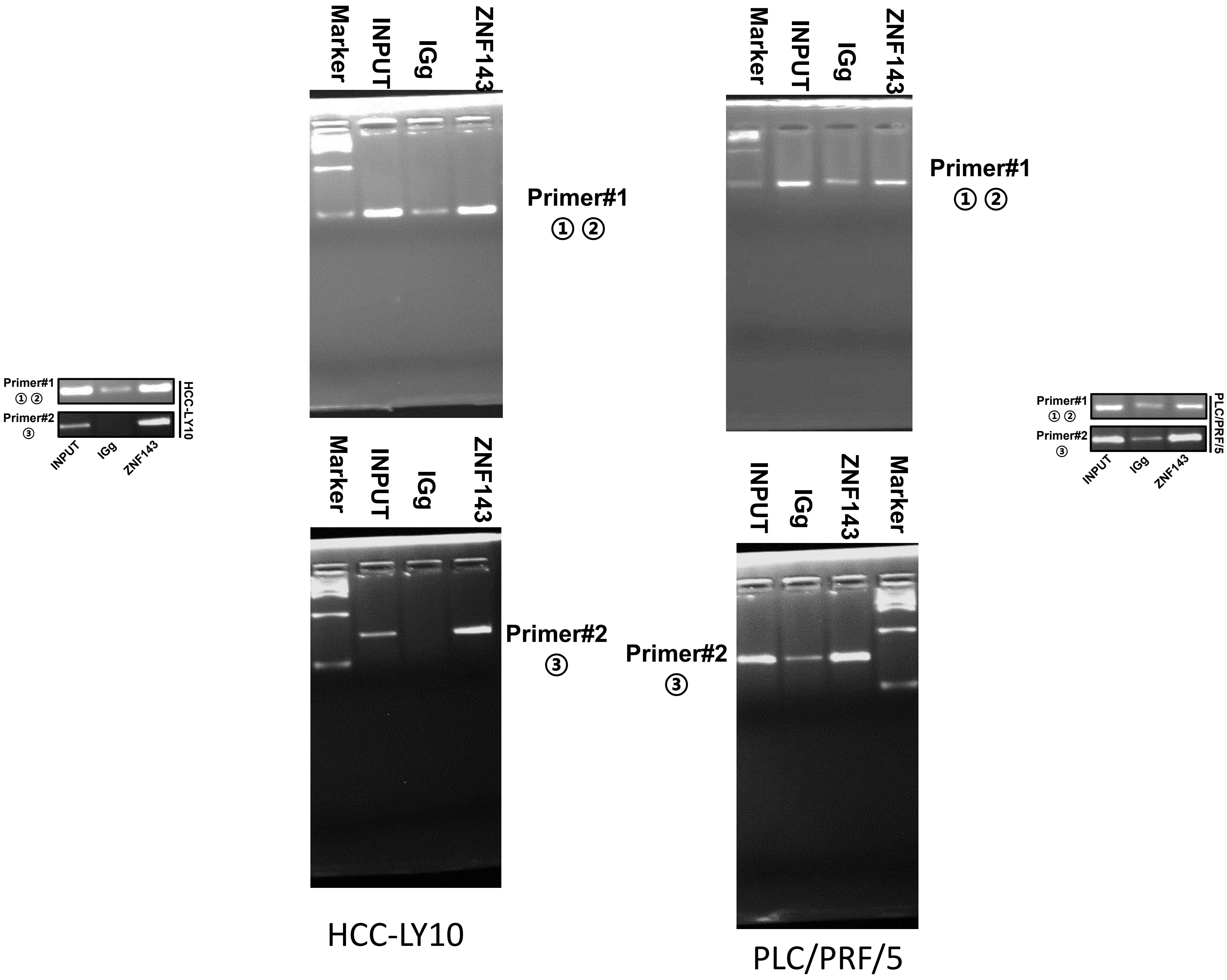

Primer#1
① ②
Primer#2
③
INPUT
IGg
ZNF143
HCC-LY10
Marker
INPUT
IGg
ZNF143
Primer#1
① ②
Marker
INPUT
IGg
ZNF143
Primer#2
③
Marker
INPUT
IGg
ZNF143
Primer#1
① ②
INPUT
IGg
ZNF143
Marker
Primer#2
③
Primer#1
① ②
Primer#2
③
INPUT
IGg
ZNF143
PLC/PRF/5
HCC-LY10
PLC/PRF/5

Figure 4A

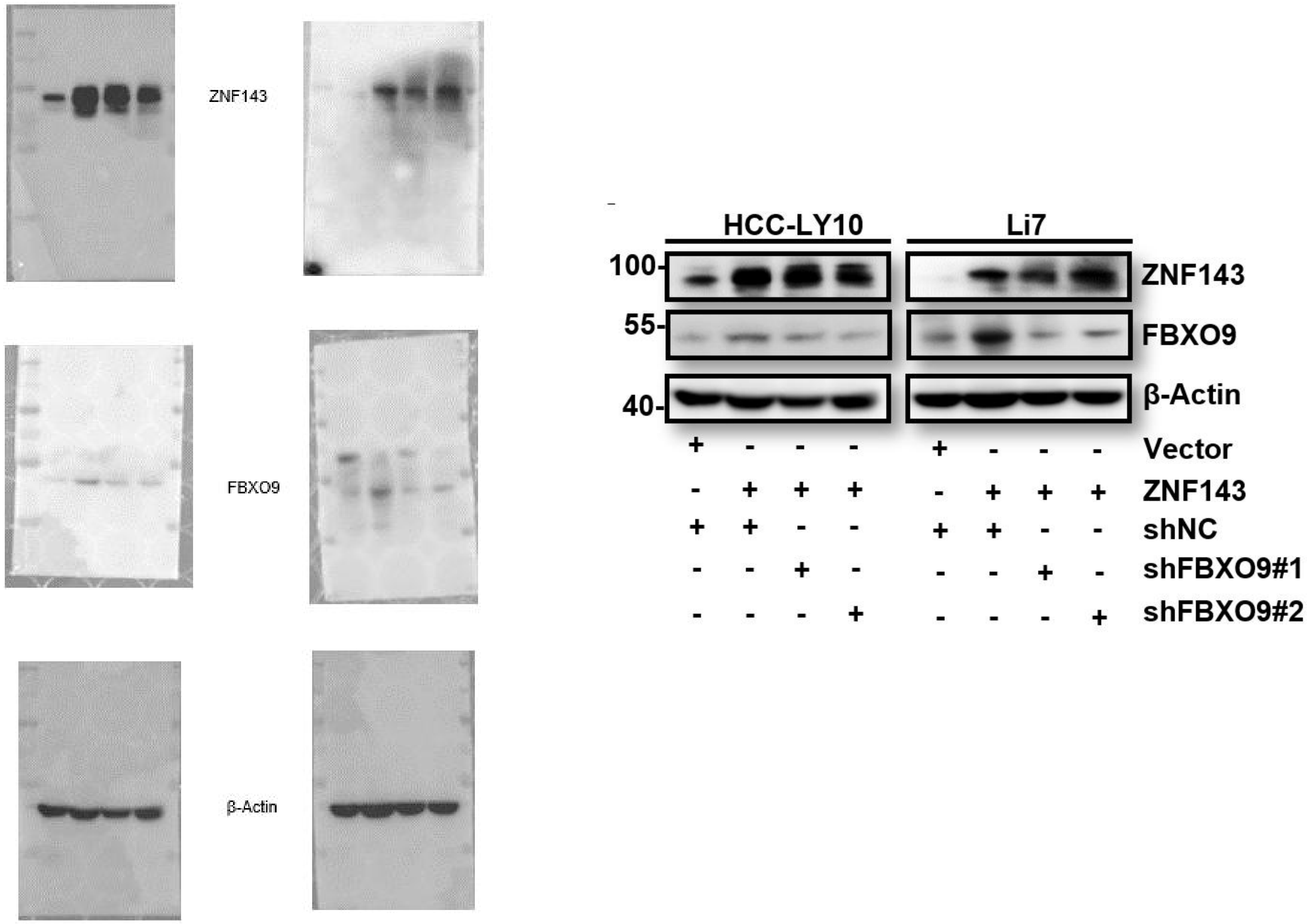
ZNF143
FBXO9
β-Actin
HCC-LY10
Li7
100-
55-
40-
ZNF143
FBXO9
β-Actin
+ - - - + - - - Vector
- + + + - + + + ZNF143
+ + - - + + - - shNC
- - + - - - + - shFBXO9#1
- - - + - - - + shFBXO9#2

Figure 4E

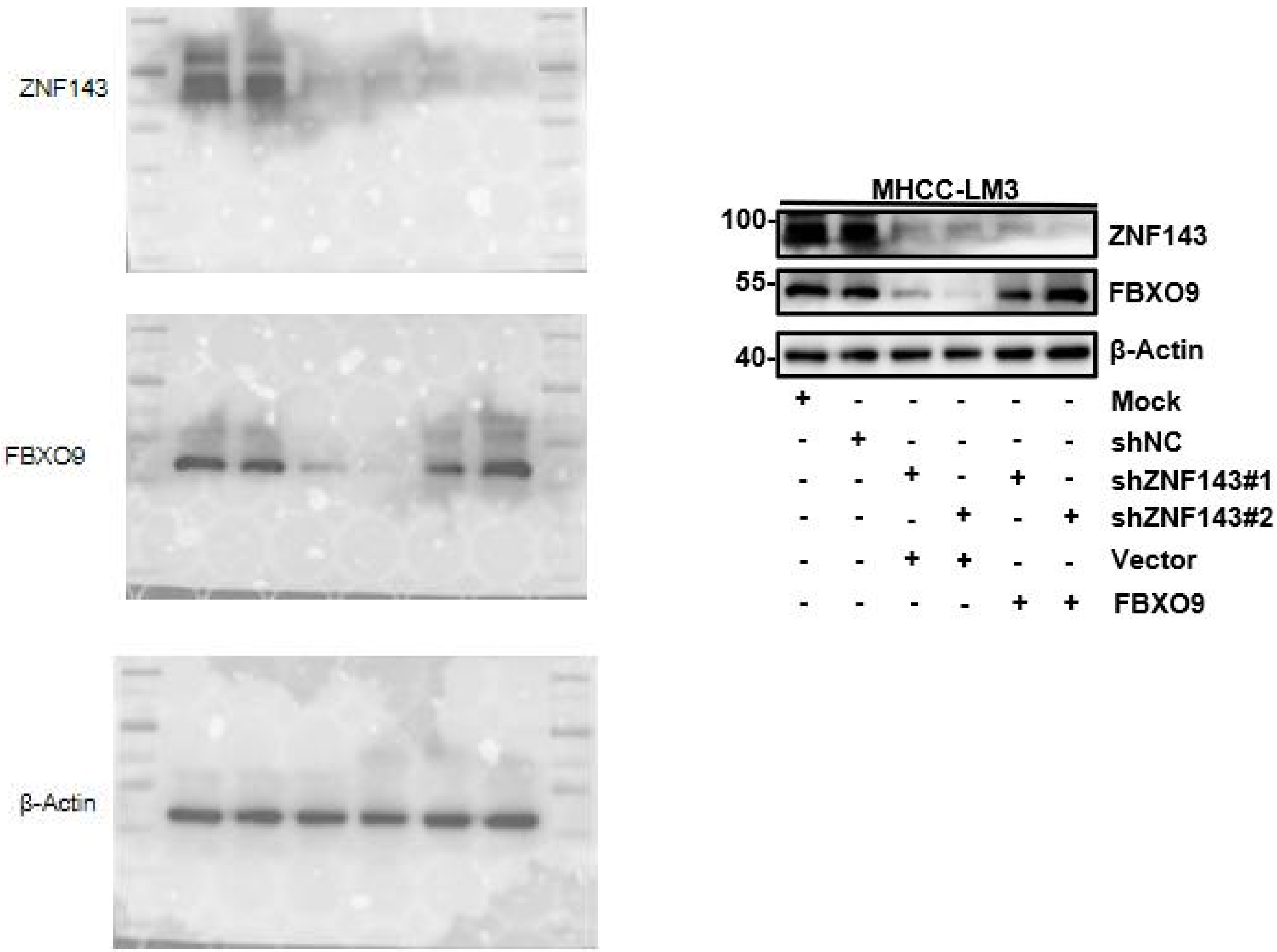
ZNF143
FBXO9
β-Actin
MHCC-LM3
100-
55-
40-
ZNF143
FBXO9
β-Actin
+ - - - - - Mock
- + - - - - shNC
- - + - + - shZNF143#1
- - - + - + shZNF143#2
- - + + - - Vector
- - - - + + FBXO9

Figure 5A

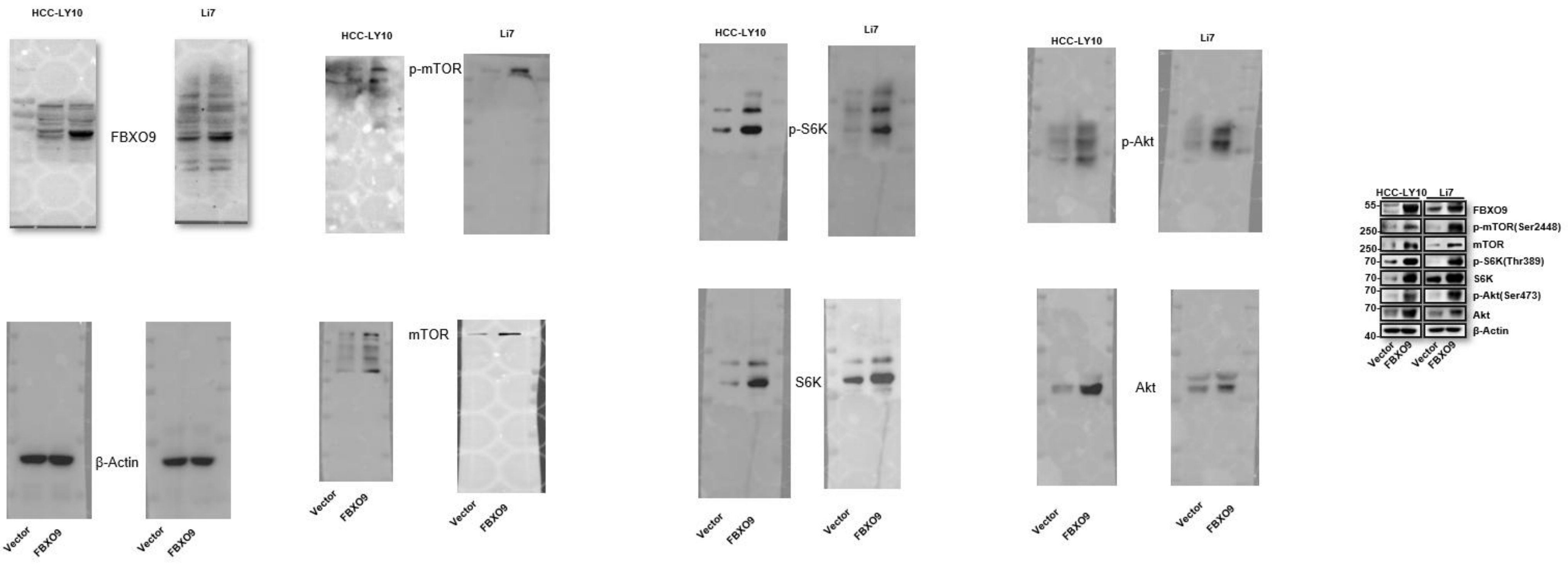

HCC-LY10
Li7
FBXO9
p-mTOR
p-S6K
p-Akt
β-Actin
mTOR
S6K
Akt
Vector
FBXO9
HCC-LY10 Li7
55-
250-
250-
70-
70-
70-
70-
40-
FBXO9
p-mTOR(Ser2448)
mTOR
p-S6K(Thr389)
S6K
p-Akt(Ser473)
Akt
β-Actin

Figure 5A

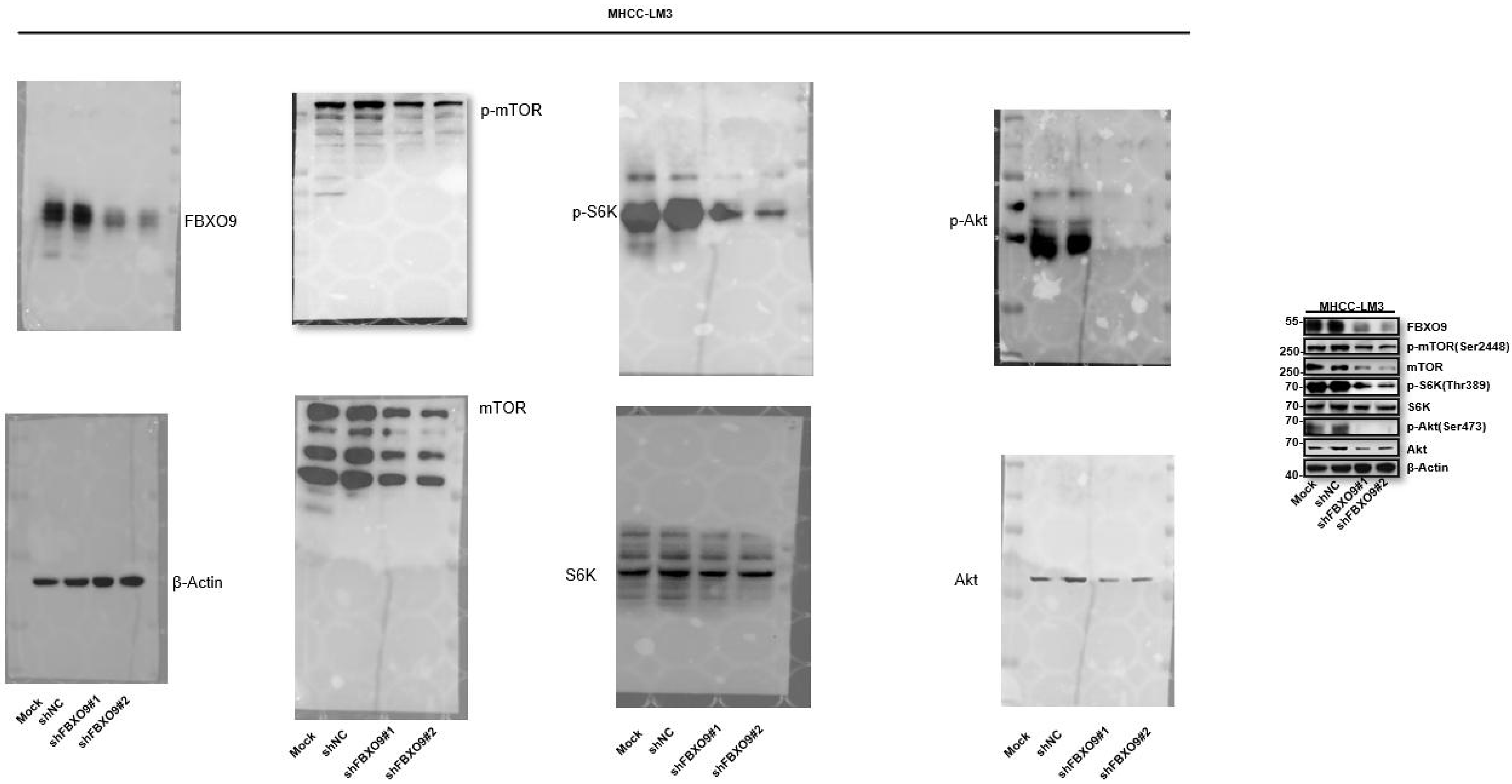
MHCC-LM3
FBXO9
p-mTOR
p-S6K
p-Akt
β-Actin
mTOR
S6K
Akt
Mock
shNC
shFBXO9#1
shFBXO9#2
55
250
250
70
70
70
70
40
FBXO9
p-mTOR(Ser2448)
mTOR
p-S6K(Thr389)
S6K
p-Akt(Ser473)
Akt
β-Actin

Figure 5A

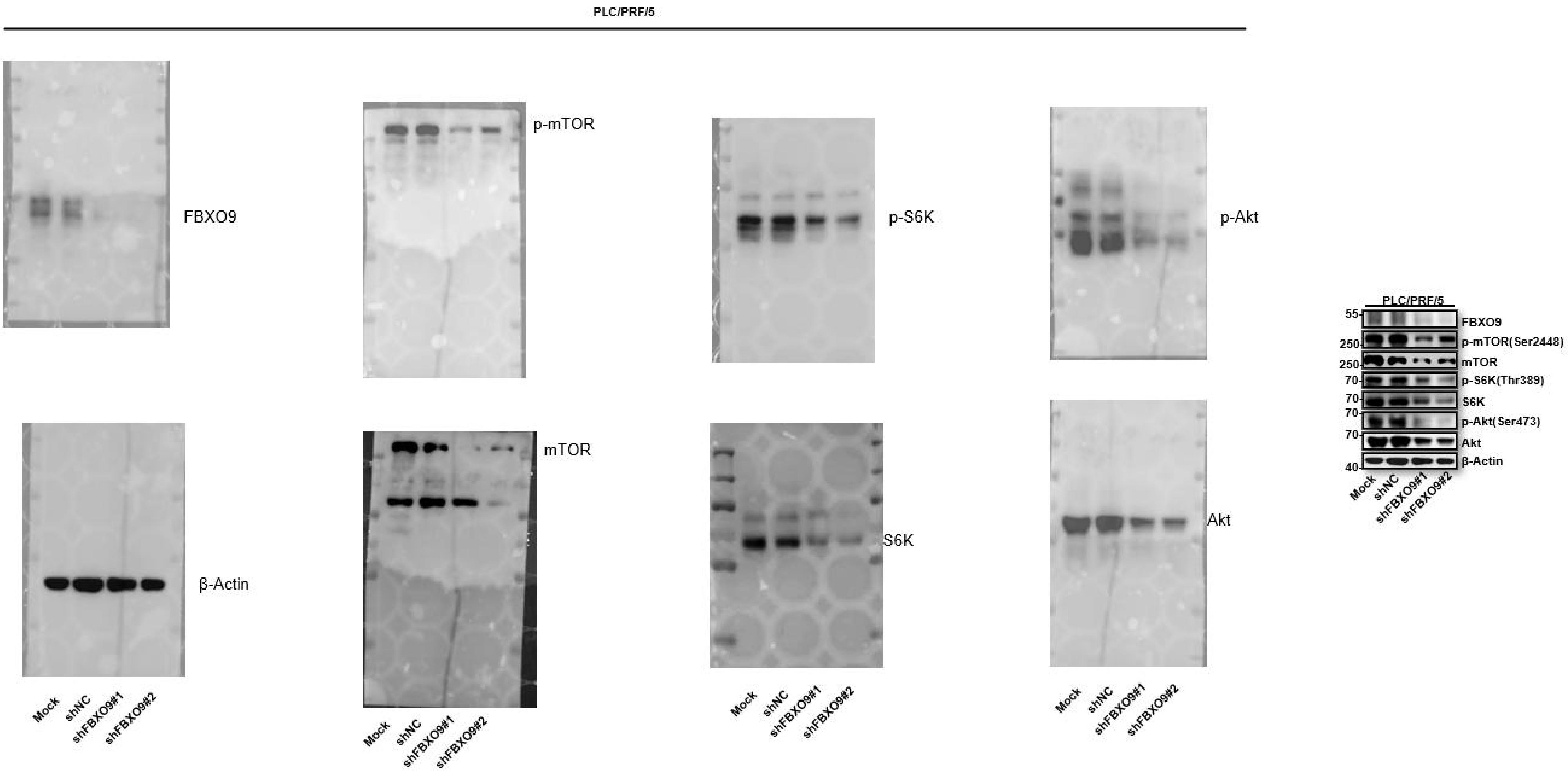

PLC/PRF/5
FBXO9
p-mTOR
p-S6K
p-Akt
β-Actin
mTOR
S6K
Akt
Mock
shNC
shFBXO9#1
shFBXO9#2
PLC/PRF/5
55
250
250
70
70
70
70
40
FBXO9
p-mTOR(Ser2448)
mTOR
p-S6K(Thr389)
S6K
p-Akt(Ser473)
Akt
β-Actin

Figure 5B

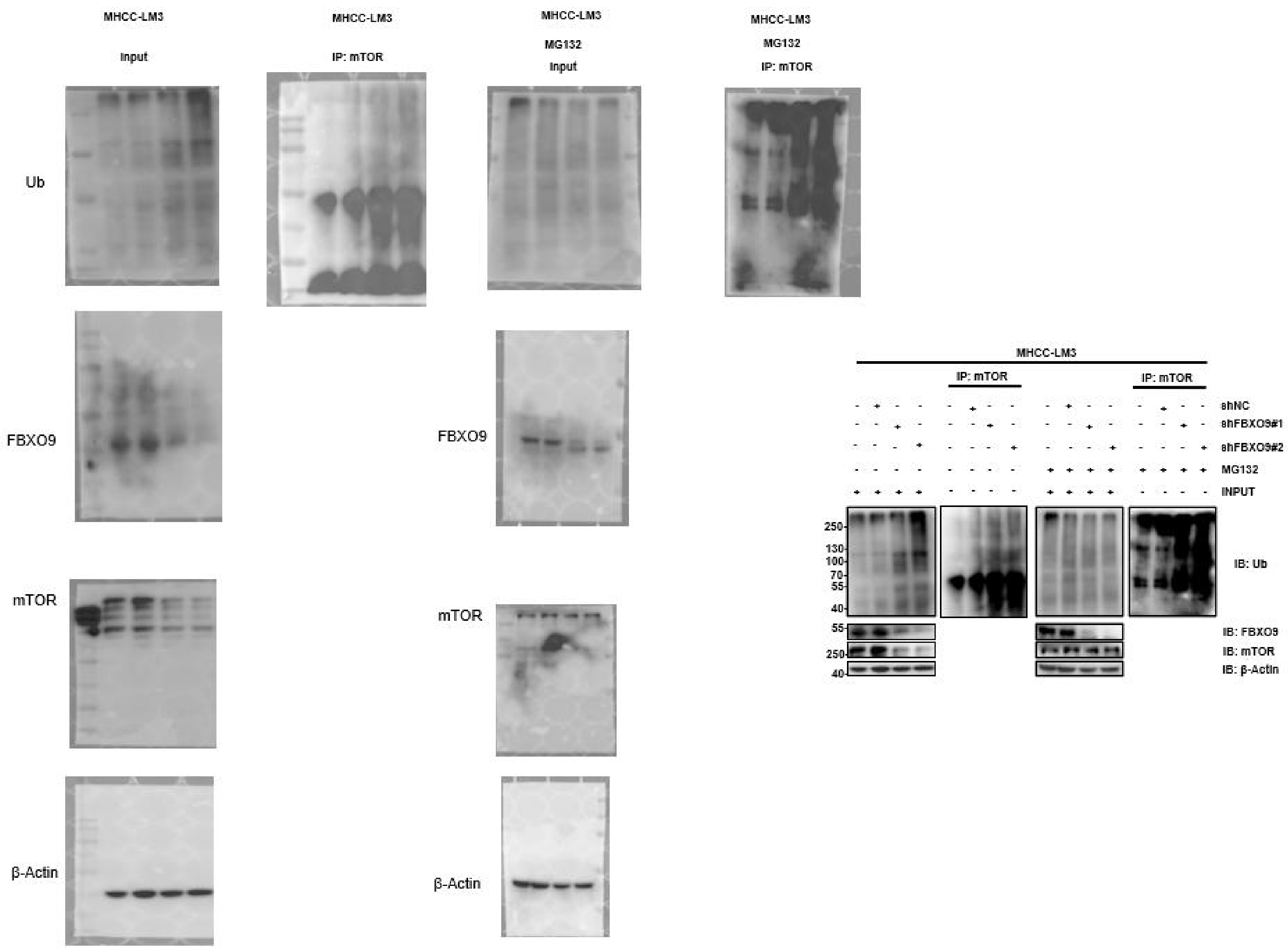
MHCC-LM3
Input
MHCC-LM3
IP: mTOR
MHCC-LM3
MG132
Input
MHCC-LM3
MG132
IP: mTOR
Ub
FBXO9
mTOR
β-Actin
FBXO9
mTOR
β-Actin
MHCC-LM3
IP: mTOR
IP: mTOR
shNC
shFBXO9#1
shFBXO9#2
MG132
INPUT
250
130
100
70
55
40
IB: Ub
55
250
40
IB: FBXO9
IB: mTOR
IB: β-Actin

Figure 5C

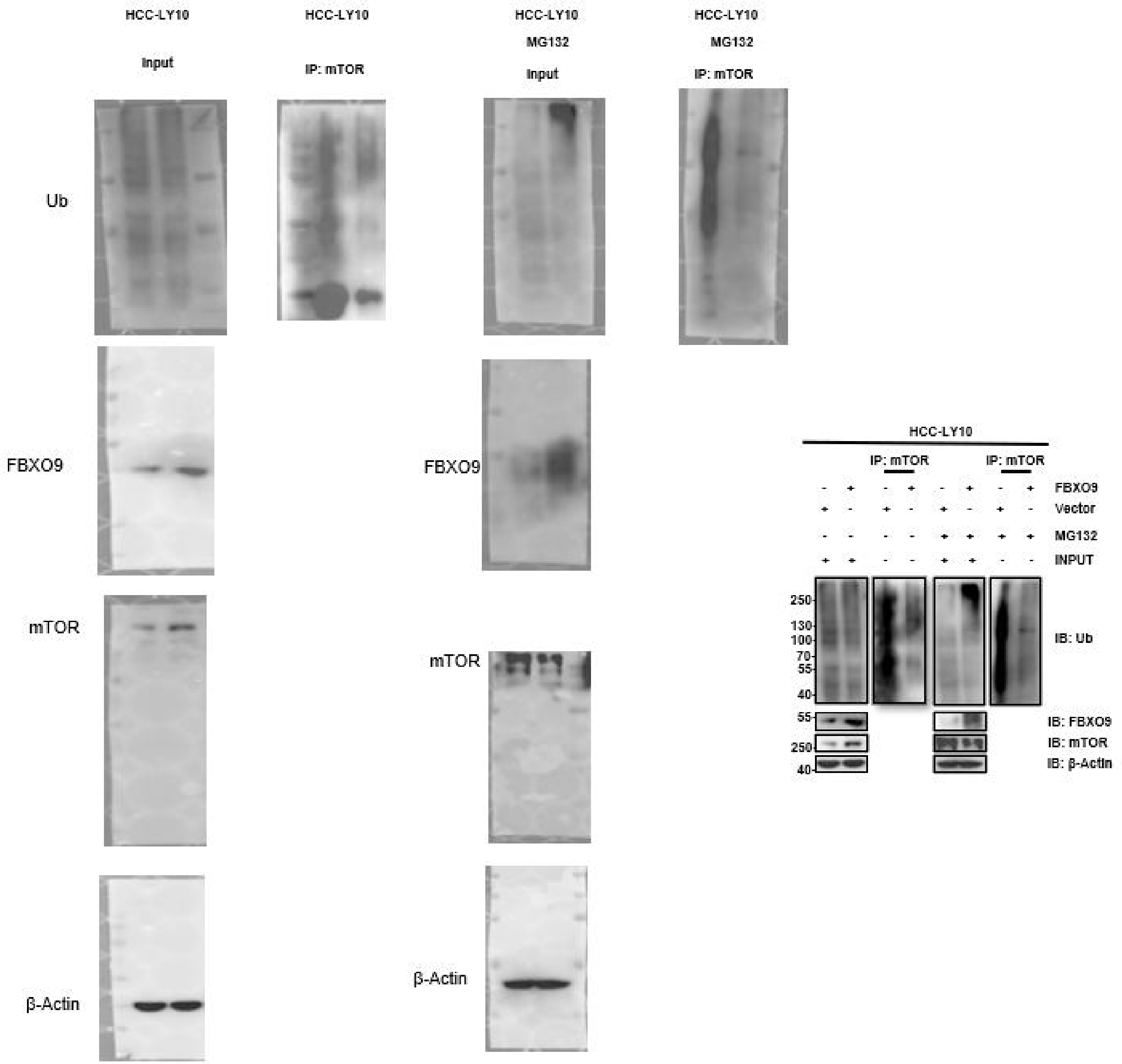
HCC-LY10
Input
HCC-LY10
IP: mTOR
HCC-LY10
MG132
Input
HCC-LY10
MG132
IP: mTOR
Ub
FBXO9
mTOR
β-Actin
FBXO9
mTOR
β-Actin
HCC-LY10
IP: mTOR
IP: mTOR
FBXO9
Vector
MG132
INPUT
250
130
100
70
55
40
IB: Ub
55
250
40
IB: FBXO9
IB: mTOR
IB: β-Actin

Figure 5D-E

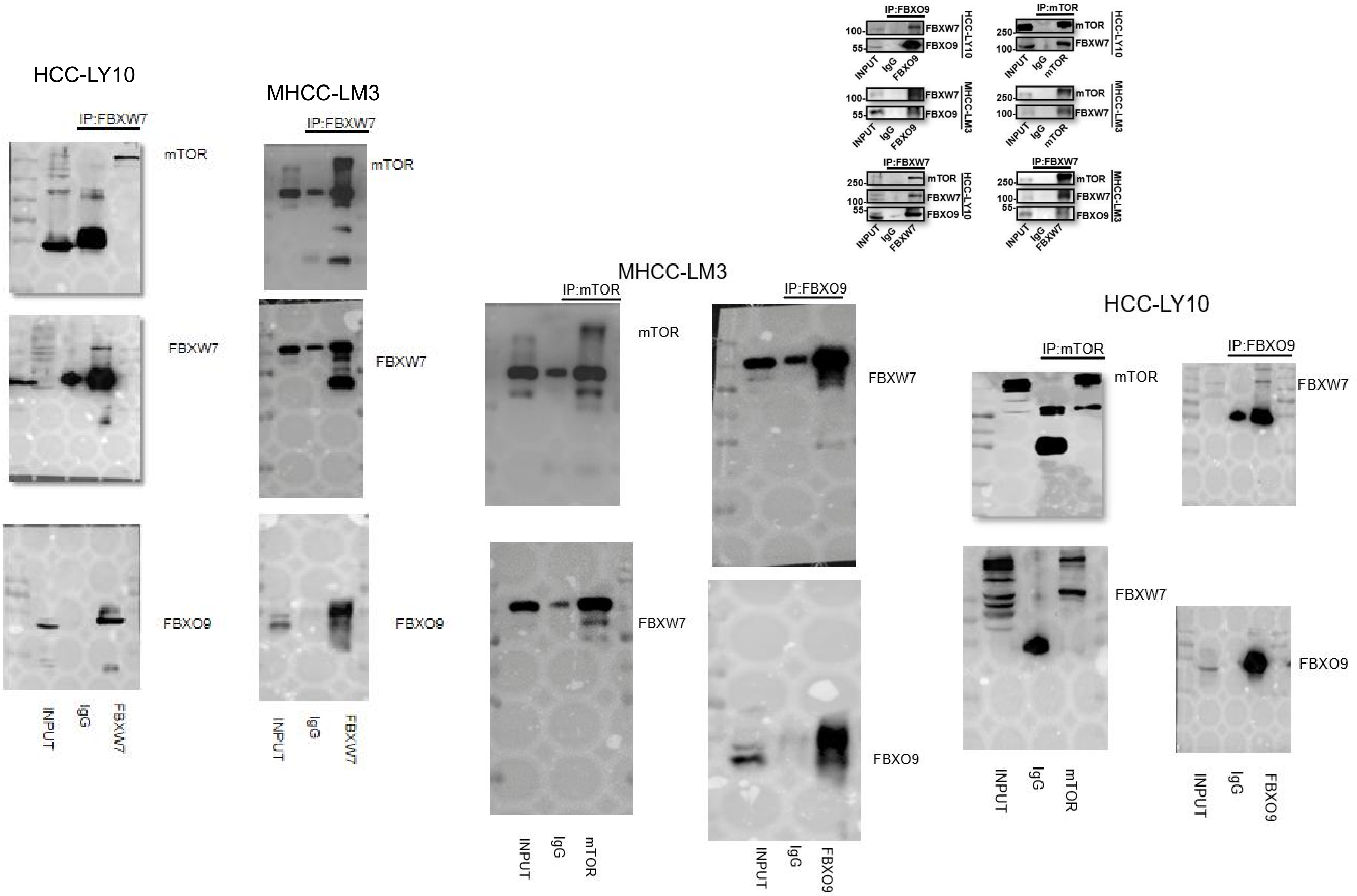

HCC-LY10
IP:FBXW7
mTOR
FBXW7
FBXO9
INPUT
IgG
FBXW7
MHCC-LM3
IP:FBXW7
mTOR
FBXW7
FBXO9
INPUT
IgG
FBXW7
MHCC-LM3
IP:mTOR
mTOR
FBXW7
INPUT
IgG
mTOR
IP:FBXO9
FBXW7
FBXO9
INPUT
IgG
FBXO9
HCC-LY10
IP:mTOR
mTOR
FBXW7
INPUT
IgG
mTOR
IP:FBXO9
FBXW7
FBXO9
INPUT
IgG
FBXO9
IP:FBXO9
100-
55-
FBXW7
FBXO9
HCC-LY10
INPUT
IgG
FBXO9
IP:mTOR
250-
100-
mTOR
FBXW7
HCC-LY10
INPUT
IgG
mTOR
MHCC-LM3
IP:FBXW7
250-
100-
55-

Figure 5F

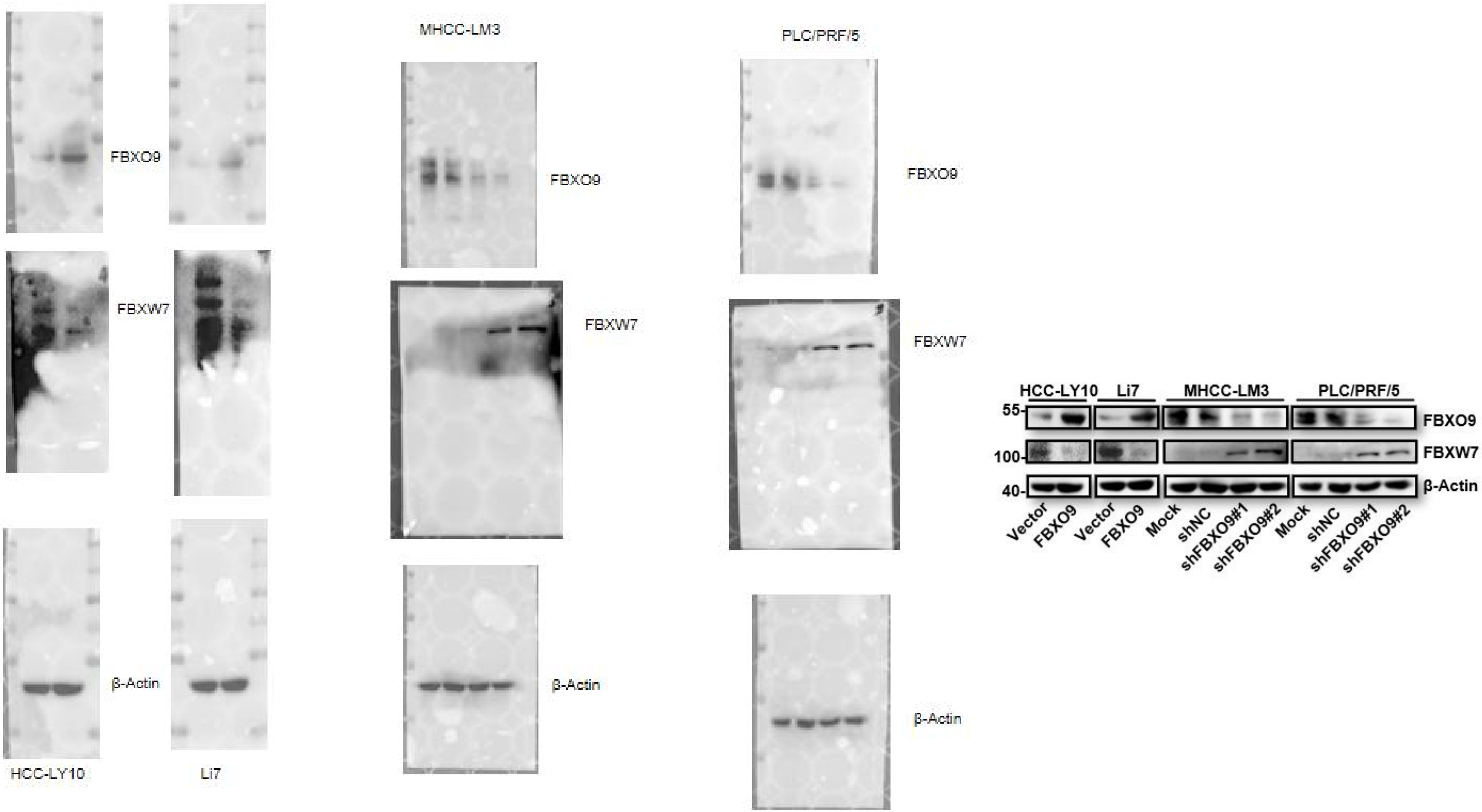

FBXO9
FBXW7
β-Actin
HCC-LY10
Li7
MHCC-LM3
FBXO9
FBXW7
β-Actin
PLC/PRF/5
FBXO9
FBXW7
β-Actin
HCC-LY10
Li7
MHCC-LM3
PLC/PRF/5
55-
100-
40-
FBXO9
FBXW7
β-Actin
Vector
FBXO9
Vector
FBXO9
Mock
shNC
shFBXO9#1
shFBXO9#2
Mock
shNC
shFBXO9#1
shFBXO9#2

Figure 5G

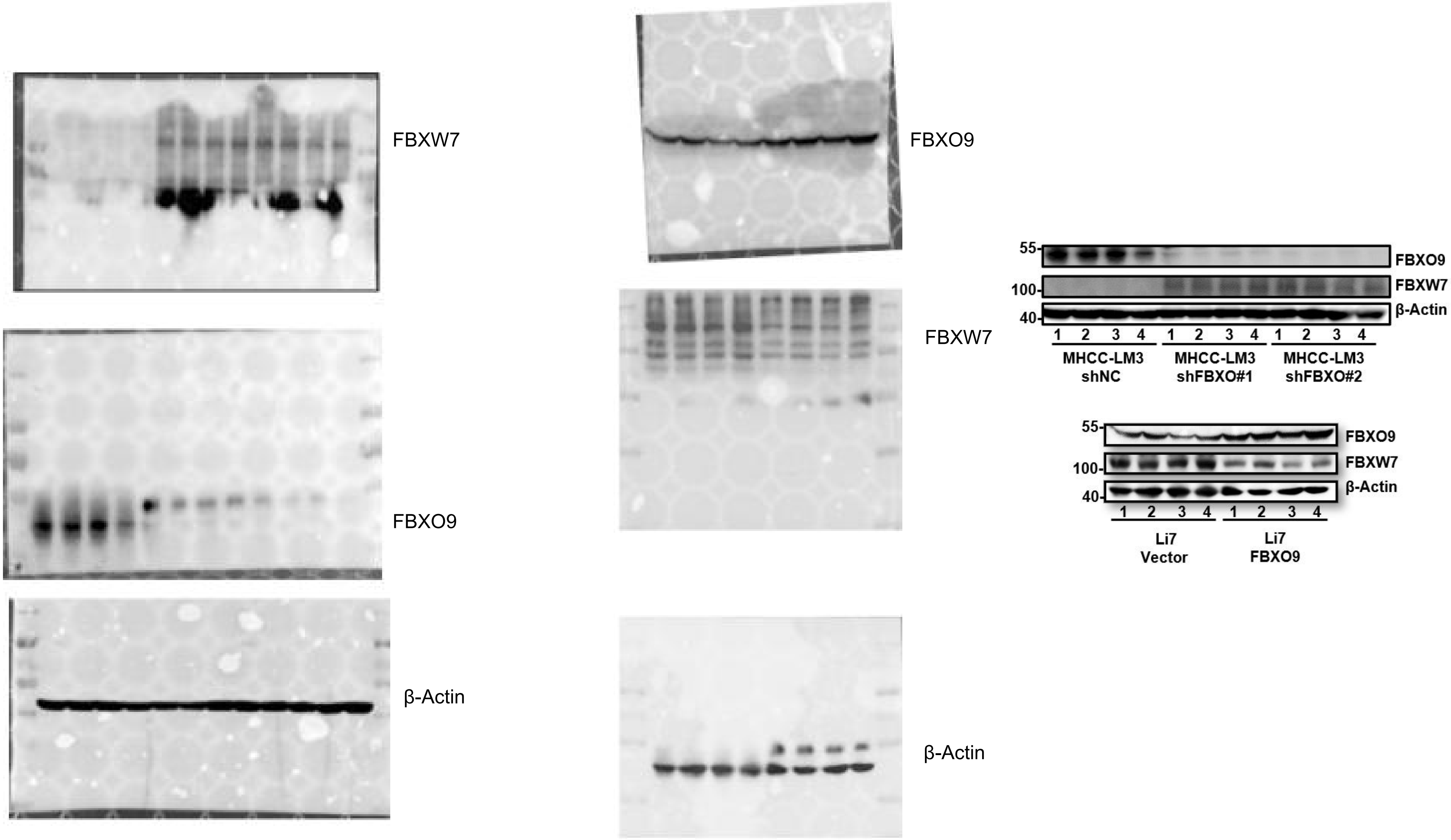
FBXW7
FBXO9
β-Actin
FBXO9
FBXW7
β-Actin
55-
FBXO9
100-
FBXW7
β-Actin
40-
1 2 3 4 1 2 3 4 1 2 3 4
MHCC-LM3 shNC
MHCC-LM3 shFBXO#1
MHCC-LM3 shFBXO#2
55-
FBXO9
100-
FBXW7
β-Actin
40-
1 2 3 4 1 2 3 4
Li7 Vector
Li7 FBXO9

Figure 5H

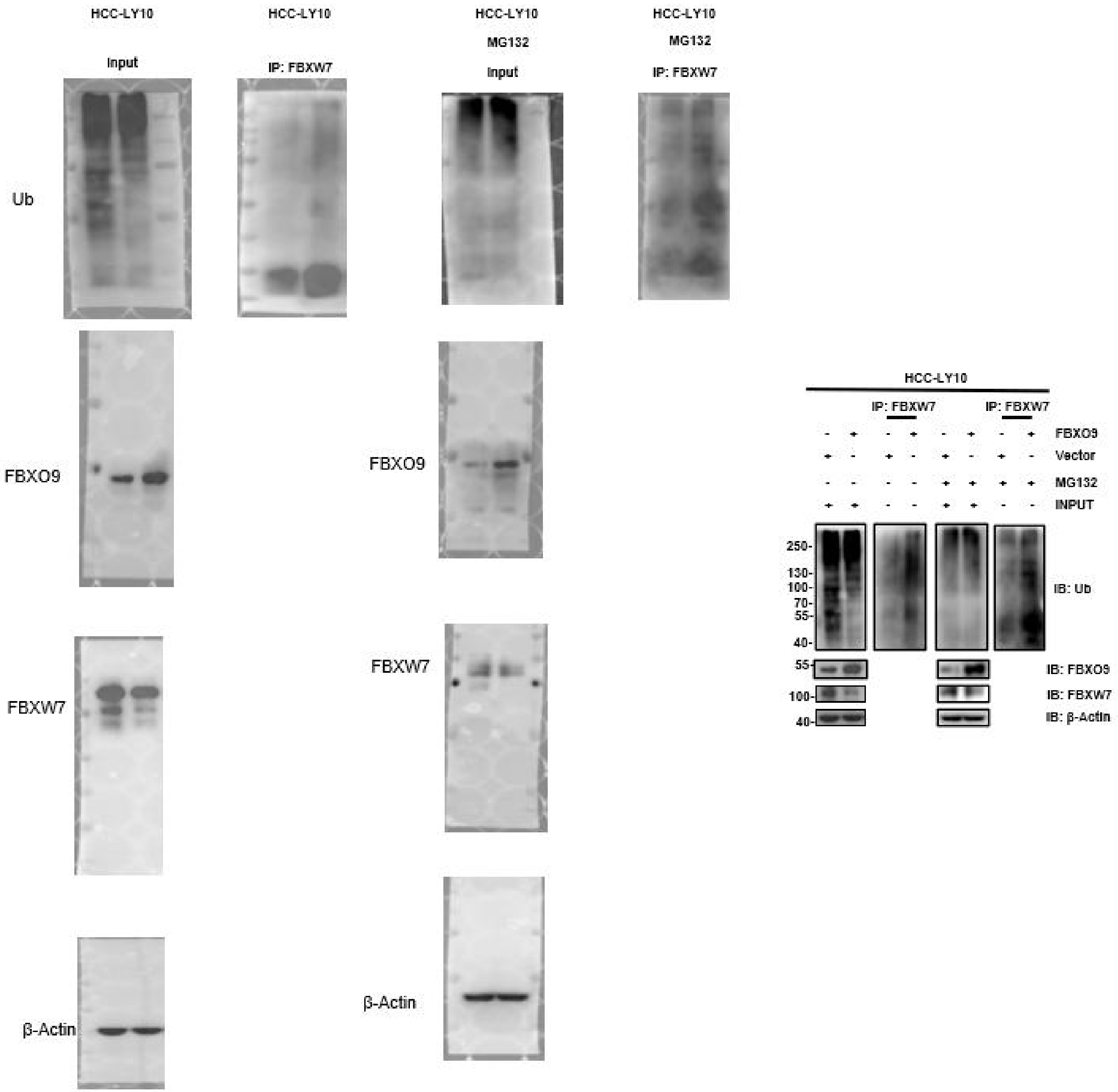
HCC-LY10
Input
HCC-LY10
IP: FBXW7
HCC-LY10
MG132
Input
HCC-LY10
MG132
IP: FBXW7
Ub
FBXO9
FBXW7
β-Actin
FBXO9
FBXW7
β-Actin
HCC-LY10
IP: FBXW7
IP: FBXW7
FBXO9
Vector
MG132
INPUT
250-
130-
100-
70-
55-
40-
IB: Ub
55-
IB: FBXO9
100-
IB: FBXW7
40-
IB: β-Actin

Figure 5I

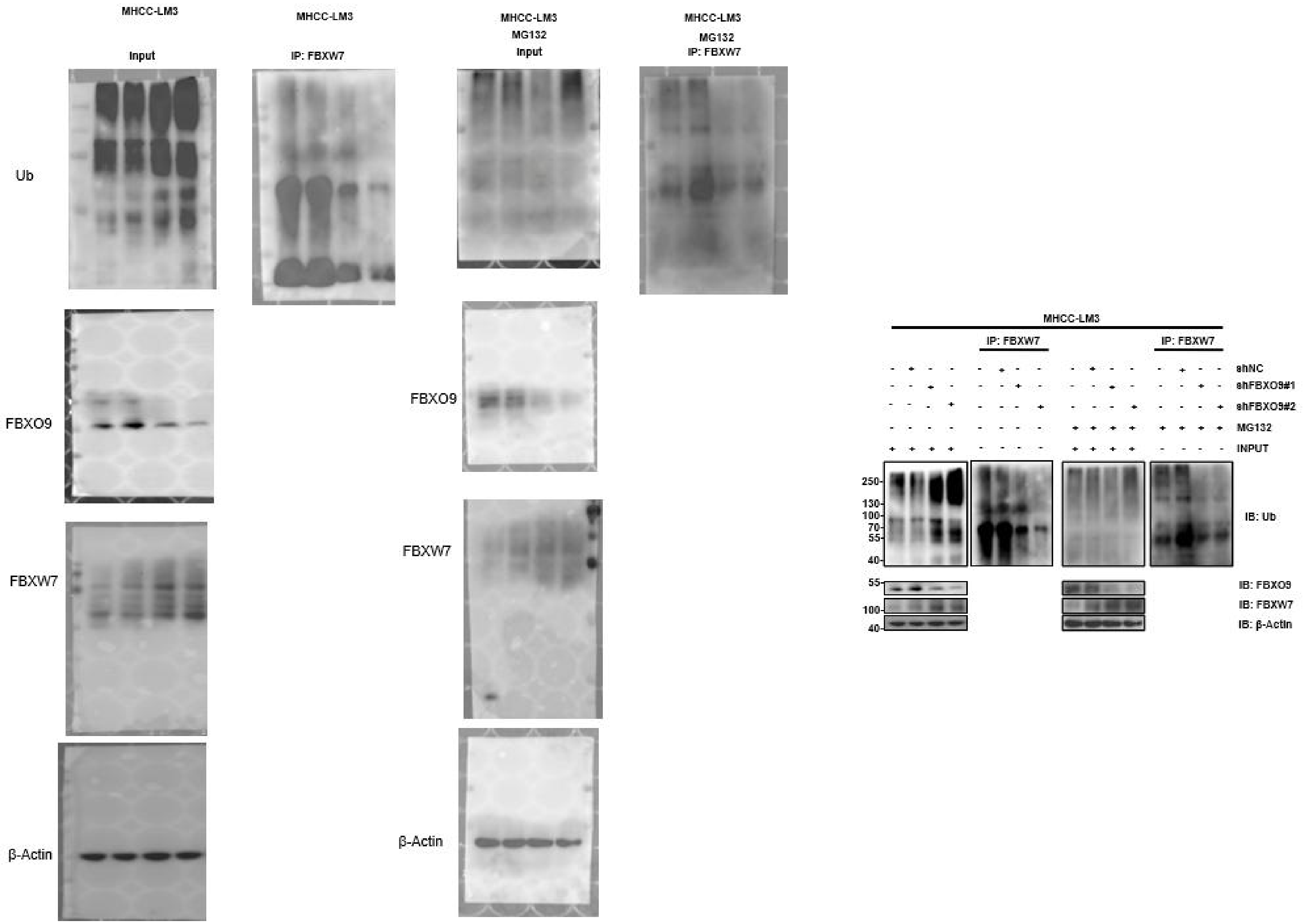

MHCC-LM3
Input
MHCC-LM3
IP: FBXW7
MHCC-LM3
MG132
Input
MHCC-LM3
MG132
IP: FBXW7
Ub
FBXO9
FBXW7
β-Actin
FBXO9
FBXW7
β-Actin
MHCC-LM3
IP: FBXW7
IP: FBXW7
shNC
shFBXO9#1
shFBXO9#2
MG132
INPUT
250
130
100
70
55
40
55
100
40
IB: Ub
IB: FBXO9
IB: FBXW7
IB: β-Actin

Figure 5J

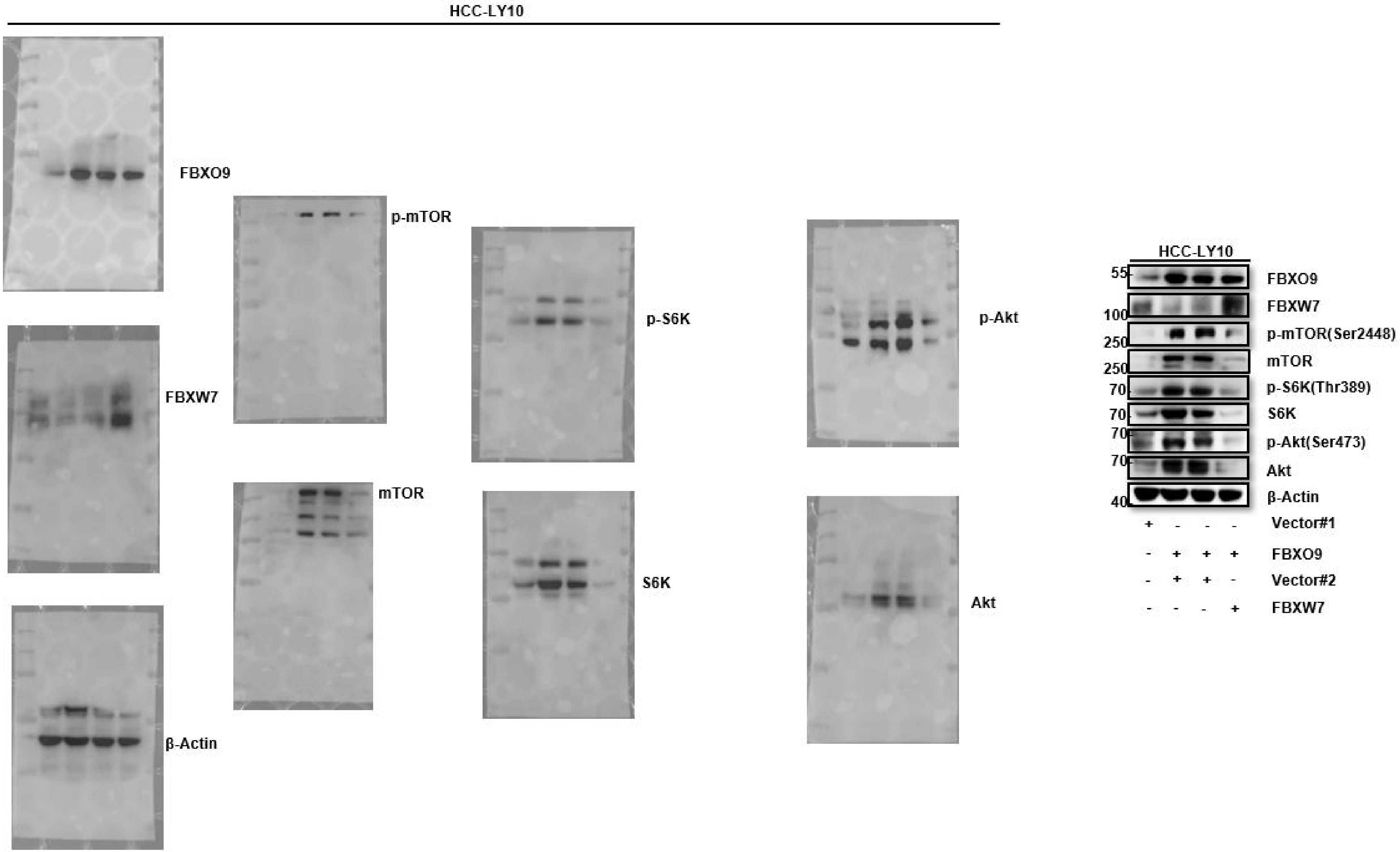
HCC-LY10
FBXO9
p-mTOR
p-S6K
p-Akt
FBXW7
mTOR
S6K
Akt
β-Actin
HCC-LY10
55
100
250
250
70
70
70
70
40
FBXO9
FBXW7
p-mTOR(Ser2448)
mTOR
p-S6K(Thr389)
S6K
p-Akt(Ser473)
Akt
β-Actin
+ - - - Vector#1
- + + + FBXO9
- + + - Vector#2
- - - + FBXW7

Figure 5J

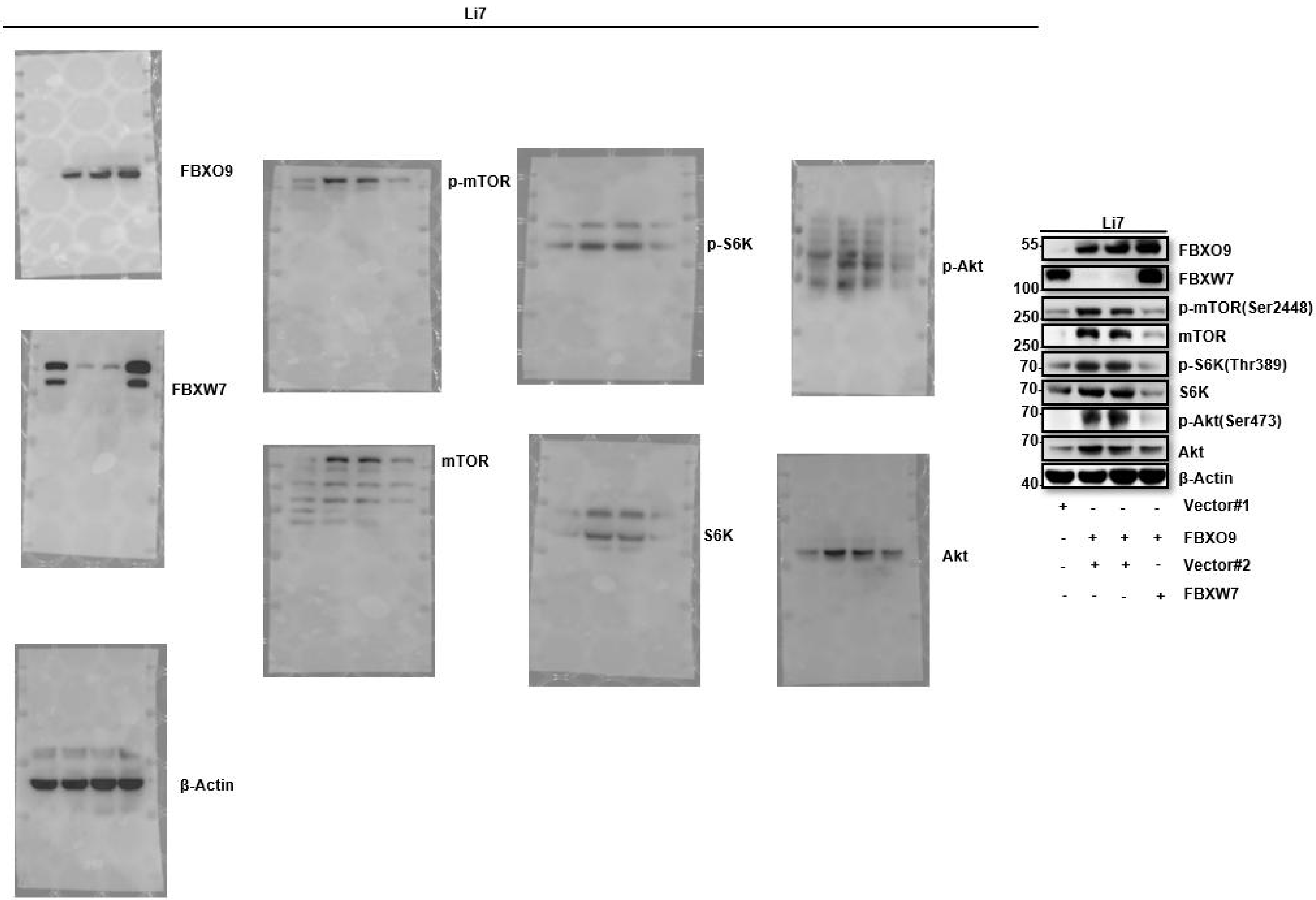
Li7
FBXO9
FBXW7
β-Actin
p-mTOR
mTOR
p-S6K
S6K
p-Akt
Akt
Li7
55
100
250
250
70
70
70
70
40
FBXO9
FBXW7
p-mTOR(Ser2448)
mTOR
p-S6K(Thr389)
S6K
p-Akt(Ser473)
Akt
β-Actin
+ - - - Vector#1
- + + + FBXO9
- + + - Vector#2
- - - + FBXW7

Figure S7

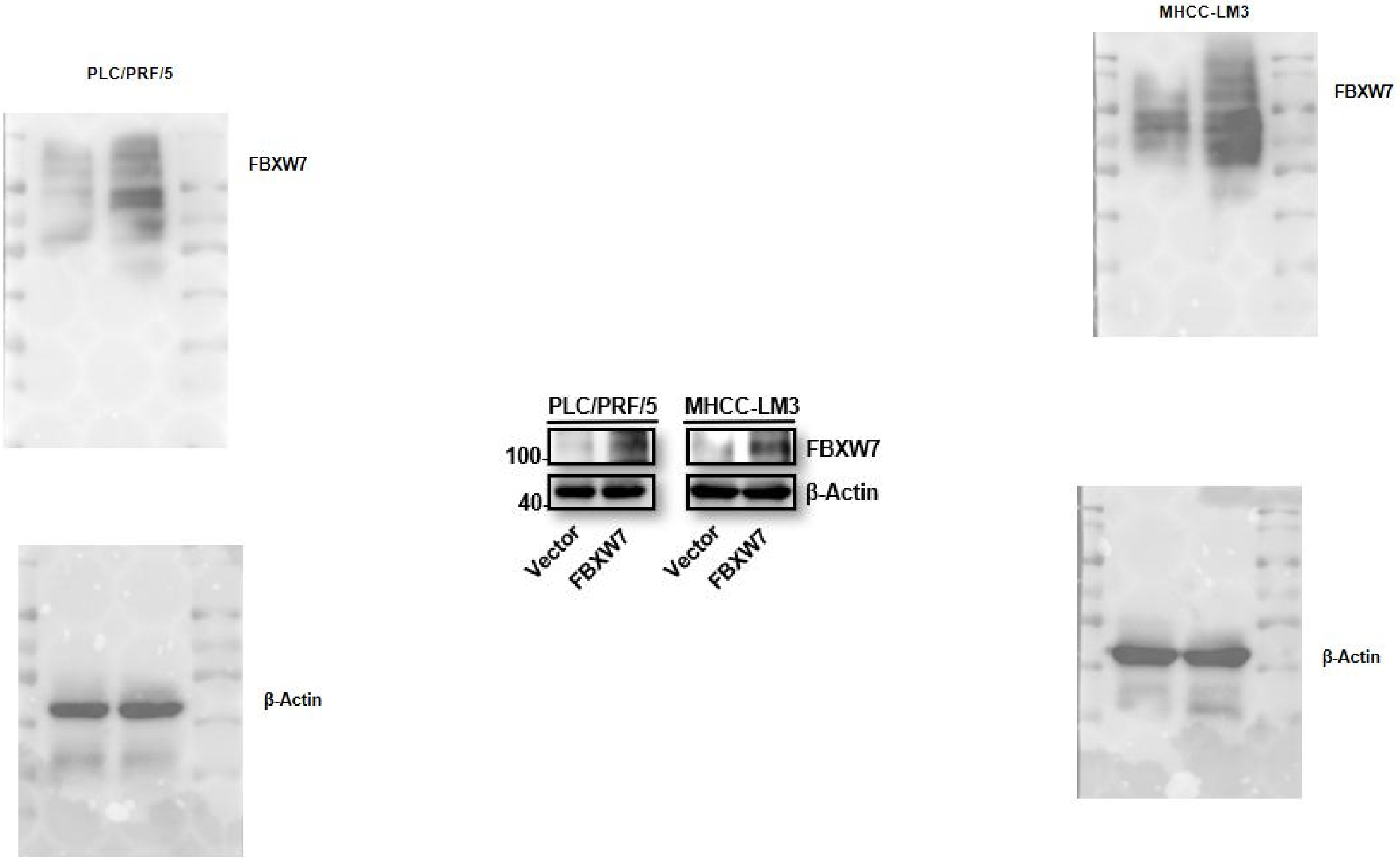
PLC/PRF/5
FBXW7
β-Actin
PLC/PRF/5
MHCC-LM3
100
40
FBXW7
β-Actin
Vector
FBXW7
Vector
FBXW7
MHCC-LM3
FBXW7
β-Actin

Figure S8

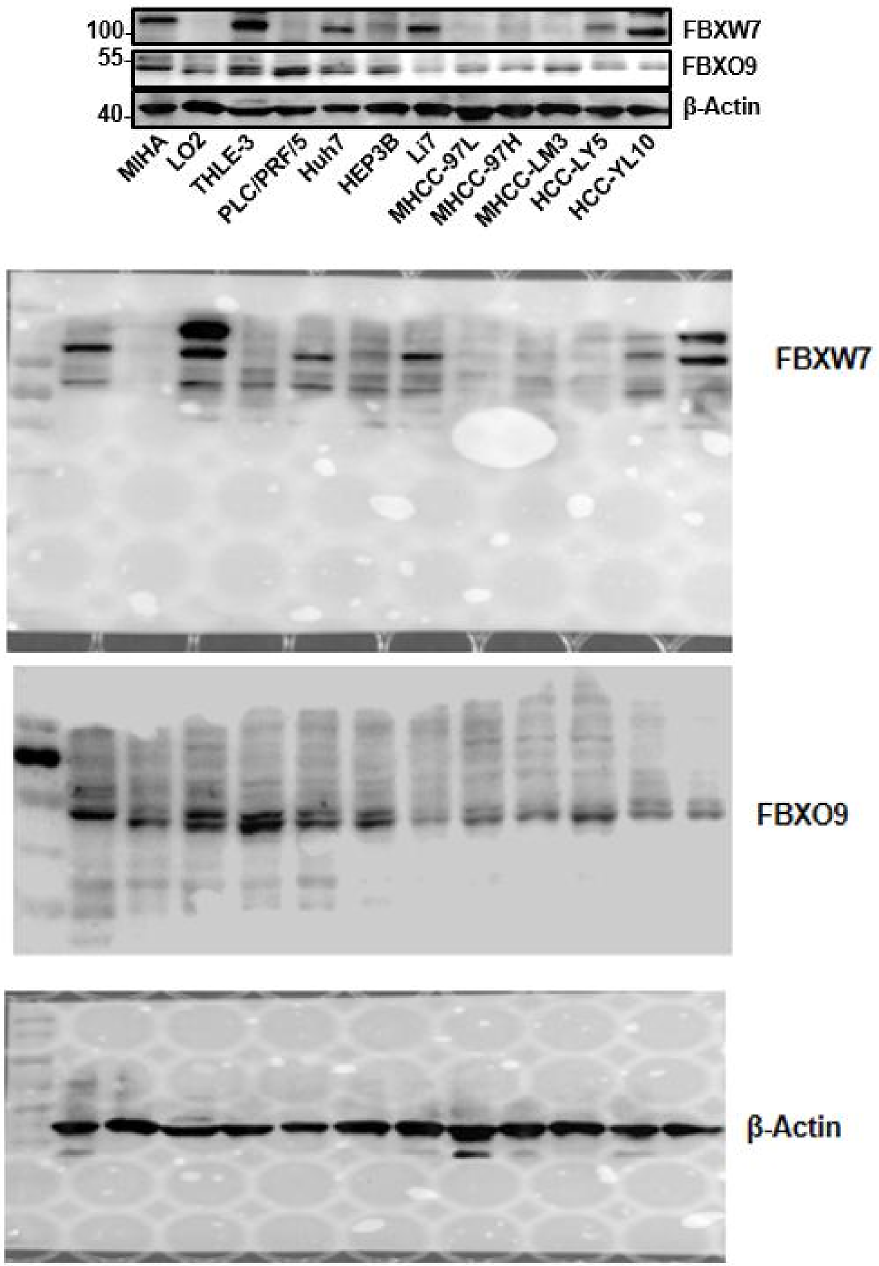
100
55
40
FBXW7
FBXO9
β-Actin
MIHA
LO2
THLE-3
PLC/PRF/5
Huh7
HEP3B
Li7
MHCC-97L
MHCC-97H
MHCC-LM3
HCC-LY5
HCC-YL10
FBXW7
FBXO9
β-Actin

Figure 7A

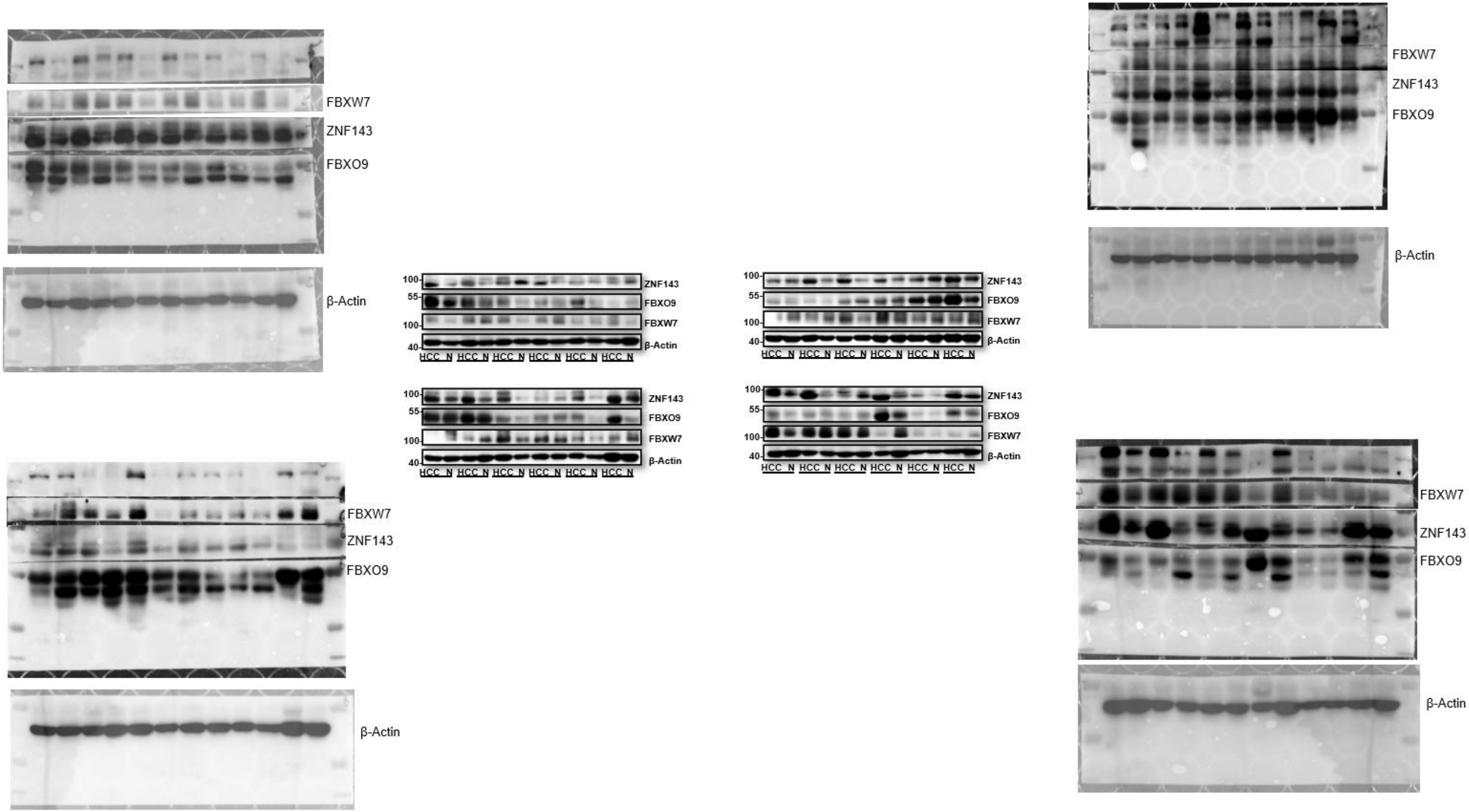

Figure 7A

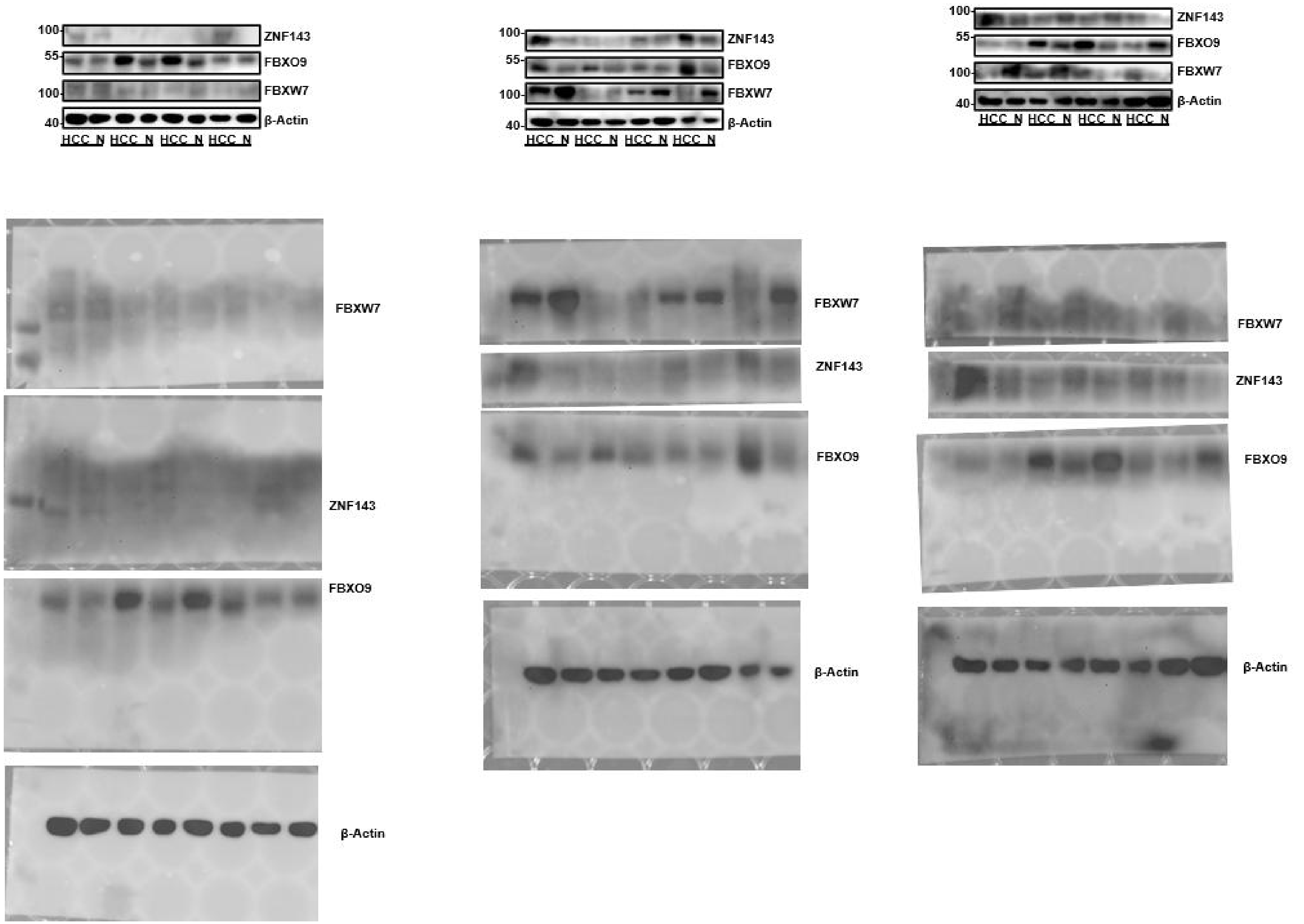

100
55
100
40
ZNF143
FBXO9
FBXW7
β-Actin
HCC N HCC N HCC N HCC N
FBXW7
ZNF143
FBXO9
β-Actin

Figure 7A

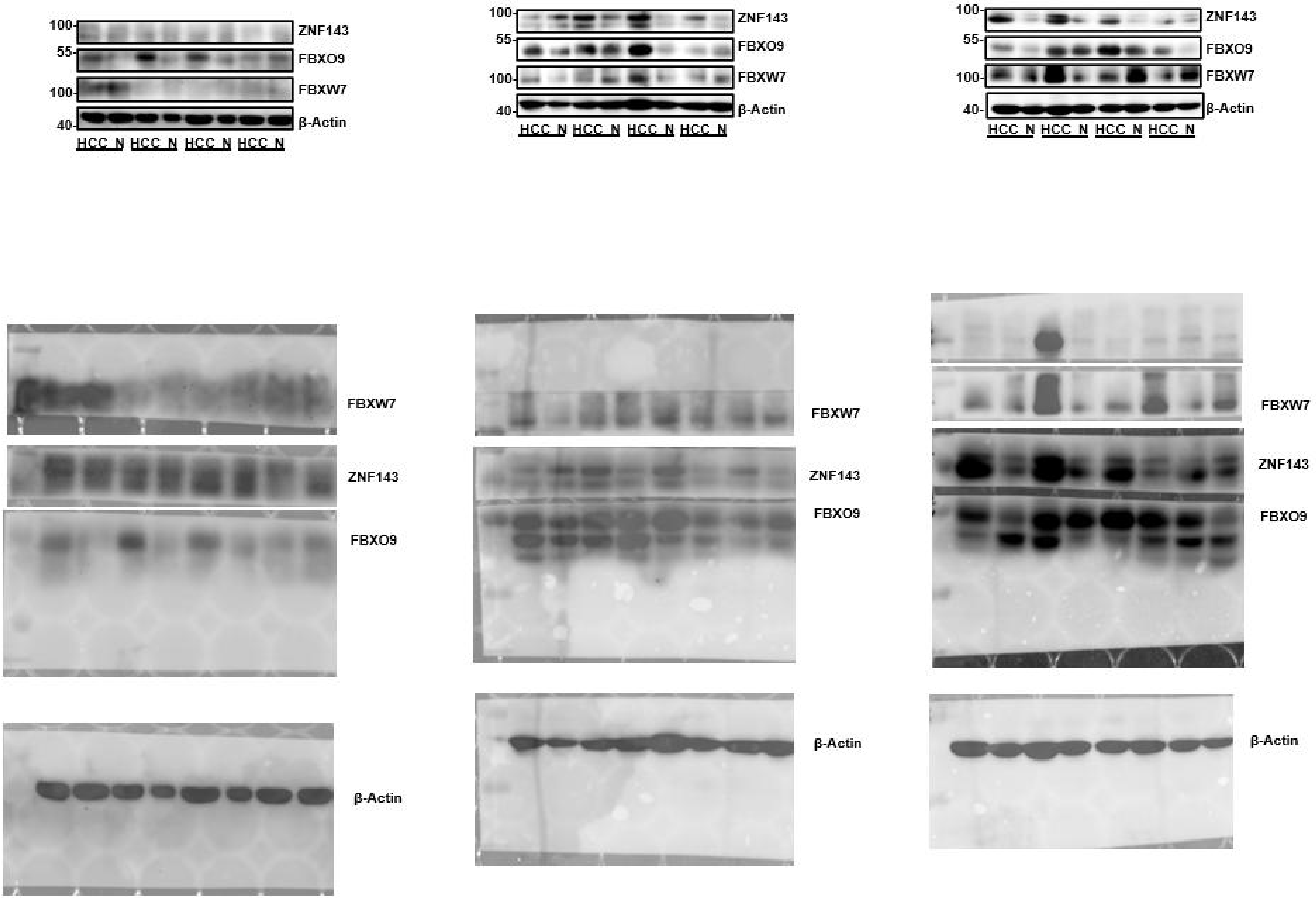
100
55
100
40
ZNF143
FBXO9
FBXW7
β-Actin
HCC N HCC N HCC N HCC N
FBXW7
ZNF143
FBXO9
β-Actin
